# Can a decision support system accelerate rare disease diagnosis? Evaluating the potential impact of Ada DX in a retrospective study

**DOI:** 10.1186/s13023-019-1040-6

**Published:** 2019-03-21

**Authors:** Simon Ronicke, Martin C. Hirsch, Ewelina Türk, Katharina Larionov, Daphne Tientcheu, Annette D. Wagner

**Affiliations:** 1 Outpatient clinic for rare inflammatory systemic diseases, Department of Nephrology, Hannover Medical School, Carl-Neuberg-Straße 1, Hannover, 30625 Germany; 2 Ada Health GmbH, Adalbertstraße 20, Berlin, 10997 Germany

**Keywords:** Rare disease diagnosis, Diagnostic decision support system, Time to diagnosis, Ada DX, Artificial intelligence, Probabilistic reasoning

## Abstract

**Background:**

Rare disease diagnosis is often delayed by years. A primary factor for this delay is a lack of knowledge and awareness regarding rare diseases. Probabilistic diagnostic decision support systems (DDSSs) have the potential to accelerate rare disease diagnosis by suggesting differential diagnoses for physicians based on case input and incorporated medical knowledge. We examine the DDSS prototype Ada DX and assess its potential to provide accurate rare disease suggestions early in the course of rare disease cases.

**Results:**

Ada DX suggested the correct disease *earlier* than the time of clinical diagnosis among the top five fit disease suggestions in 53.8% of cases (50 of 93), and as the top fit disease suggestion in 37.6% of cases (35 of 93). The median advantage of correct disease suggestions compared to the time of clinical diagnosis was 3 months or 50% for top five fit and 1 month or 21% for top fit. The correct diagnosis was suggested at the *first* documented patient visit in 33.3% (top 5 fit), and 16.1% of cases (top fit), respectively. Wilcoxon signed-rank test shows a significant difference between the time to clinical diagnosis and the time to correct disease suggestion for both top five fit and top fit (z-score -6.68, respective -5.71, *α*=0.05, p-value <0.001).

**Conclusion:**

Ada DX provided accurate rare disease suggestions in most rare disease cases. In many cases, Ada DX provided correct rare disease suggestions early in the course of the disease, sometimes at the very beginning of a patient journey. The interpretation of these results indicates that Ada DX has the potential to suggest rare diseases to physicians early in the course of a case. Limitations of this study derive from its retrospective and unblinded design, data input by a single user, and the optimization of the knowledge base during the course of the study. Results pertaining to the system’s accuracy should be interpreted cautiously. Whether the use of Ada DX reduces the time to diagnosis in rare diseases in a clinical setting should be validated in prospective studies.

**Electronic supplementary material:**

The online version of this article (10.1186/s13023-019-1040-6) contains supplementary material, which is available to authorized users.

## Background

By definition, every rare disease is rare. However, together rare diseases are common. Globally, about 350 million people are affected [1]. One in 17 people will be affected by a rare disease in their lifetime [2]. Rare disease diagnosis remains a challenge for patients, doctors, and healthcare systems. Rare disease patients often have diagnostic odysseys, waiting an average of 6 years from onset of symptoms for an accurate diagnosis [3]. Misdiagnosis and incorrect treatment are frequent in rare diseases [4]. During the diagnostic odyssey patients suffer from loss of quality of life, disease progression, incorrect treatment and complications that are sometimes irreversible [5]. People living with rare diseases suffer an even greater loss of quality of life than people with common chronic diseases [5]. Common examples of personal consequences are anxiety, frustration, and impacted relationships [3, 6, 7]. At the same time, unnecessary consultations cause substantial costs for the individual and for healthcare systems. Before the correct diagnosis is made, patients see an average of 7.3 physicians [7]. Therefore, there is an urgent need to improve rare disease diagnosis [3].

### The challenge of diagnosing rare diseases

Reasons for delayed diagnosis and frequent misdiagnosis of rare diseases are not well understood. Insufficient knowledge and lack of awareness are considered to be the main factors, particularly in primary care [4]. Overall, rare disease diagnosis presents itself as a cognitive challenge due to a combination of factors that characterize rare diseases and limitations of the human brain. There are approximately 7000 rare diseases [1, 2]. Due to the limited capacity of the human brain, primary care physiciansPCPs) cannot know about every rare disease. PCPs will only infrequently encounter rare diseases in practice. Even rare disease specialists will not have in-depth knowledge of every rare disease. The heterogeneity of rare diseases is an additional complicating factor [4]. Low incidence in combination with a large number of possible rare conditions almost inevitably leads to insufficient disease knowledge and diagnostic errors. For example, lack of condition-specific knowledge contributes to errors and faulty verification when alternative diagnoses are not taken into account after a first diagnosis has been established – a phenomenon known as premature closure [8]. Premature closure appears to be among the most common single types of error in medicine [8] and can be assumed to be of major importance in rare diseases. Insufficient knowledge about rare diseases subsequently causes error, for example via fragmentary assessment of history and examination or incomplete diagnostic testing.

The exponential growth of knowledge in the medical domain further contributes to the cognitive overload. Not only is the number of known diseases increasing, but the available diagnostic methods and possible interpretations in the medical domain are expanding continuously [9]. In the near future, rapid developments in genetics are likely to lead to an even higher complexity of rare disease diagnoses. Physicians will require support to overcome this cognitive challenge.

### New technology on the horizon

With new technological developments and artificial intelligence (AI) on the horizon it is expected that new tools will become available that are able to incorporate large amounts of medical knowledge. These tools can empower physicians in their clinical work by effectively enhancing their cognitive performance. In view of the challenge of rare disease diagnosis, such technology could help PCPs correctly identify and refer rare disease patients. Institutions such as the UK Department of Health and the German National Action League for People with Rare Diseases (NAMSE) recommend the development of appropriate rare disease research tools that indicate potential rare diseases based on a given constellation of symptoms [10, 11]. This would require capable support systems for rare diseases.

### Diagnostic decision support systems

Diagnostic decision support systems (DDSSs) are expert systems that support physicians by facilitating the diagnostic reasoning process. DDSSs have the potential to enhance clinical diagnosis by assessing case data based on incorporated medical knowledge [12–14], compiling lists of differential diagnoses appropriate for a given sample of evidence [15, 16]. Some systems are already able to provide accurate disease suggestions [17]. DDSSs suggesting possible rare diseases early in the course of disease could increase diagnostic accuracy and thereby reduce time to diagnosis (TD) [18, 19]. NAMSE expects that rare disease suspicion or diagnosis can be accelerated by such support systems that facilitate the detection of rare diseases in primary care [11].

### Pre-existing DDSSs for rare diseases

There are several DDSSs specifically designed for rare diseases, most of which are available for free [20]. Their knowledge bases commonly include publicly available data sets such as the Human Phenotype Ontology (HPO) [21], the Orphanet database of rare diseases [22], and the Online Mendelian Inheritance in Man (OMIM) [23]. Examples of rare disease DDSSs include FindZebra [24, 25], Phenomizer [26], PhenoTips [27, 28], and Rare Disease Discovery [29]. Examples of licence-based DDSSs that are not specialized for rare diseases, but cover them to some extent, are Isabel [30] and DXplain [17].

The accuracy of DDSSs has only rarely been evaluated and compared in rare diseases cases [31]. The potential of DDSSs to reduce TD in rare disease cases remains to be evaluated.

### DDSS Ada DX

**Fig. 1:**
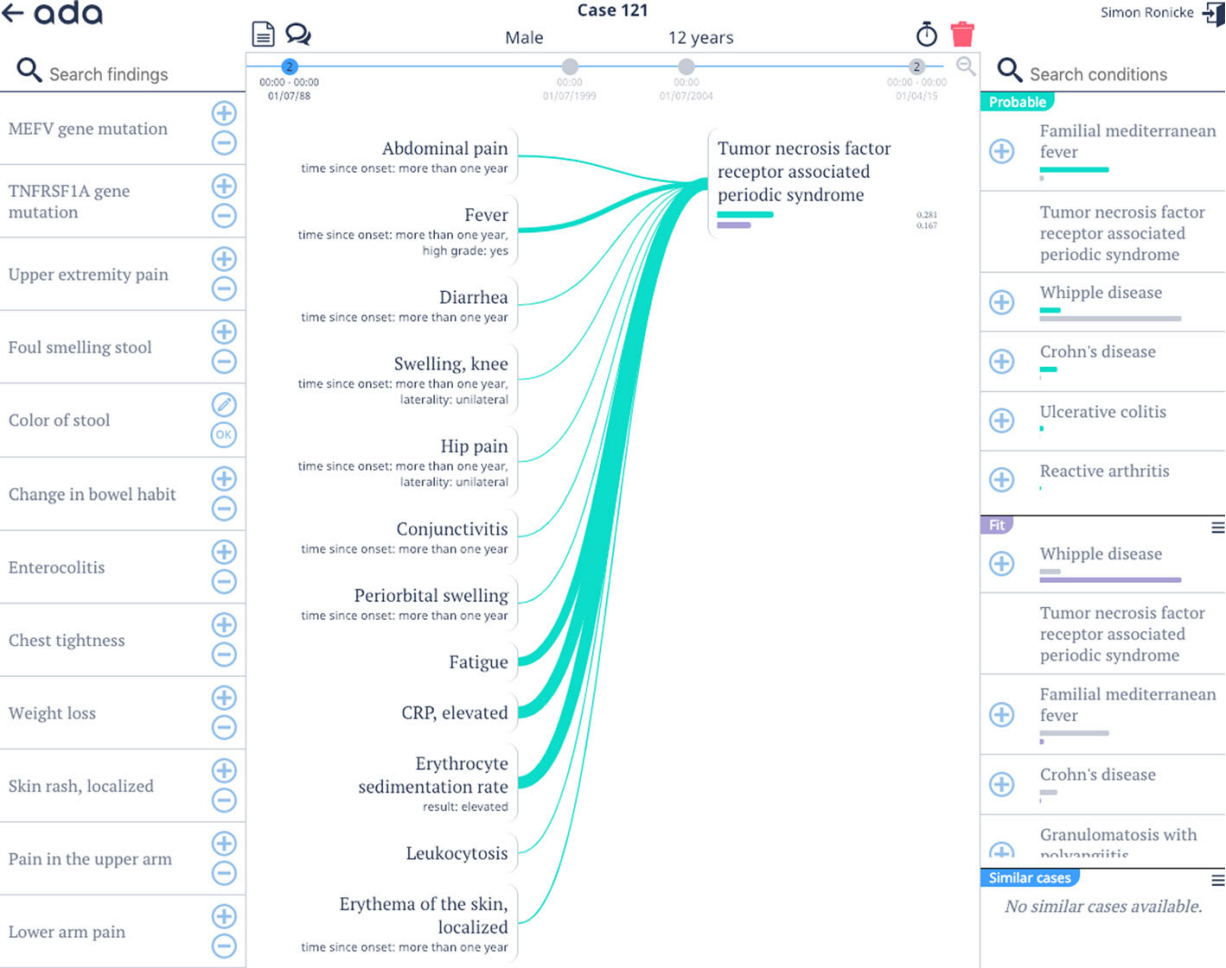
Ada DX. Screenshot of the DDSS research prototype Ada DX showing a case of a patient with TRAPS. The later confirmed diagnosis was suggested by Ada DX in an early visit. Top: Basic patient data and case timeline. Left: Symptom search and suggested symptoms. Center: Entered symptoms with their attributes, green contribution lines, selected diseases, bars visualizing disease probability (green) and fit (purple). Right: Lists of differential diagnoses ranked by ’probability’ and ’fit’ and links to similar cases

Ada DX is a professional DDSS that is currently being developed as a research prototype by Ada Health. Figure 1 shows a screenshot of Ada DX. Ada DX allows physicians to build cases over time, containing multiple visits. Symptoms can be searched and added to a case in Ada DX as present or absent findings. The system does not rely on a standard taxonomy or ontology to enter symptoms. Findings are easily found via several synonyms and terms. Alternatively, the system allows physicians to enter findings from a list of suggestions that are ranked by their estimated relevance based on the current symptom constellation, which changes in real time with the addition of each symptom. Attributes can be added to refine symptoms.

Based on the input of patient symptoms and supported by the Ada knowledge base and reasoning engine, Ada DX generates two ranked lists of differential diagnoses including both common and rare diseases: the ‘probability’ list is ranked by the disease probability estimations, and the ‘fit’ list is ranked by the estimated fit of the symptom constellation to the disease - regardless of its probability. Disease probability and fit estimations are visualized via corresponding bars. Contributions of symptoms to disease probabilities are visualized and made transparent via weighted contribution lines. Links to similar cases in the Ada DX database are presented to the physician.

The Ada DX prototype is currently available in English and German. It is easily usable in its current form as a research prototype, however, it has not yet been optimized for everyday use as a product and is therefore not yet publicly available.

#### Ada DX reasoning engine and knowledge base

Ada DX is based on a probabilistic reasoning engine that is used to infer disease probability estimations based on a representation of medical knowledge. The knowledge base was built and reviewed by medical doctors in a curated process of knowledge integration from medical literature. Disease models and their related symptoms are added to the knowledge base and modeled according to evidence from peer-reviewed medical literature. The knowledge base is being expanded continuously following this standardized process. It consists of disease models of all common conditions and several hundred rare diseases as well as their corresponding clinical findings. Clinical findings can be further refined with additional attributes, for example intensity or temporality. Epidemiological data is used to derive the prior probabilities of diseases to allow for correct disease probability estimations. The knowledge base is not based on a pre-existing database or publicly available ontology of medical content. Instead, the knowledge base has been specifically designed with the goal of diagnostic accuracy. Therefore, Ada DX has an advantage compared with databases designed for other purposes than diagnosis when used as DDSSs.

A set of several thousand internal test cases is used to continuously validate the system’s reasoning engine and knowledge base. These test cases comprise all types of diseases from different specialties, including common and rare diseases alike. The set includes cases based on medical literature (published case reports, for example) as well as typical clinical case scenarios that reflect different levels of diagnostic certainty. A team of medical doctors constantly reviews the system’s inherent medical knowledge based on these quality assurance measures. The system’s accuracy is also tested by external academic and clinical institutions and experts.

Although Ada DX primarily covers common conditions, it already comprises several hundred rare diseases. However, a future integration of HPO, Orphanet and OMIM data covering a great number of rare diseases is possible.

### The aim of this study

This is the first scientific paper on Ada DX. This study aims to evaluate the potential accuracy of Ada DX as a DDSS in rare disease cases and its potential impact on TD. The study focuses on cases in a scenario in which the system’s rare disease knowledge base was optimized for the specific medical domain: rare inflammatory systemic diseases. A secondary aim is to identify key reasons for inaccuracy and current technical limitations.

## Methods

A retrospective study was conducted at the outpatient clinic for rare inflammatory systemic diseases at the Hannover Medical School in Hannover, Germany.

### Case selection

Patients were selected from the pool of patients at the outpatient clinic. Only cases with a confirmed rare disease diagnosis and a documented date of diagnosis in their medical record were included. Cases with diagnostic odysseys were prioritized (compared to typical TD for that disease) according to a subjective evaluation by the head of the clinic for rare inflammatory systemic diseases. A strict specific timeframe definition was not applied. Cases with a low level of diagnostic certainty (with regard to respective diagnostic criteria or missing information about the diagnosis visit) were excluded.

### Case processing and input

The confirmed diagnosis was assigned to each case. In cases with multiple confirmed diagnoses all diagnoses were assigned. The assignment of diagnoses was based on the most recently specified and validated confirmatory diagnostic information from the medical record.

For each patient, all documented visits at healthcare providers were identified in the medical record. This included all visits at general practitioners, specialists and clinics. Only officially written and dated documentation was taken into account, including doctor’s discharge letters, referral notes, and documented test results from laboratory and pathology departments. The time of the visit of the first documented symptoms relatable to the confirmed diagnosis was identified, as was the time of diagnosis. TD was calculated as the period between the visit of first documented symptoms and the diagnosis visit. All dates were extracted based on year and month.

Clinical evidence including symptoms, examination findings, test results, risk factors and dates of visit were extracted from the medical record for every visit between first documented symptoms and the diagnosis. Information was assigned to the time of the respective visit, but not to earlier visits based on anamnestic information. Previously collected evidence was retained at future visits if not contradicted by other documented information. All pathological evidence (present symptoms and findings) was extracted, while non-pathological evidence (absent symptoms and findings) was only extracted if mentioned in the evaluation section of documents or when relevant for the exclusion of differential diagnoses. Extracted information was transcribed to a summary file for each case. Case summary files were pseudonymized, encrypted, and transferred to Ada Health. Case information from case summary files was entered as DDSS input data to build DDSS cases. For each case and each visit, all evidence was entered individually from the transcribed case summary. The input was selected by a single user based on the transcribed case summary files. Because information was gathered from German case files, input was performed without translation in the German version of Ada DX. Information that could not be entered because it was not found as content in Ada DX was skipped during input and noted if it related to missing confirmatory symptoms and findings. Missing disease models for confirmed diagnoses were noted as well.

### DDSS output

The DDSS displayed ranked disease suggestions in two different lists:

1. A ‘fit’ (rare) disease list ranked by the fit of the symptom constellation to the specific disease phenotypes that are represented in the disease models. The fit does not consider the prior probability of disease.
2. A ‘probability’ disease list ranked by the probabilities of the diseases being the cause of the constellation of symptoms, taking into account epidemiological information from the disease models.

Following input of all included cases, DDSS output was assessed and evaluated for each visit of each case.

### Disease suggestion accuracy

To assess the disease suggestion accuracy of Ada DX, the correctness of disease suggestions was determined for all visits. Ada DX disease suggestions were considered correct if they exactly matched the case’s confirmed diagnosis. In ambiguous cases, correctness was determined through professional review by the head of the clinic for rare inflammatory systemic diseases. In cases with multiple assigned diagnoses, correctness was defined as the presence of all assigned conditions.

### Early correct disease suggestion

To evaluate the potential impact on TD, the accuracy was assessed for all available visits in the course of the case. For this purpose, the disease suggestions in the “fit” disease list were taken into account. The fit disease list was chosen because without specific confirmatory information, rare diseases are not expected to be the most probable disease. Rather, they are expected to be considered only as a suspect diagnosis that is suggested based on the fit of the finding constellation.

The time to first correct top fit disease suggestion (TF) and the time to first correct top 5 fit disease suggestion (T5F) was determined. Correct disease suggestions were considered *early suggestions* if the period between the correct suggestion and diagnosis visit was greater than 1 month. The consistency of correct top fit and top 5 fit suggestions throughout the remaining case visits was evaluated after a correct suggestion was reached. TF/TD, as well as T5F/TD, were calculated to allow for comparison of TF and T5F normalized to TD. The disease suggestion accuracy in the first documented visits was specifically assessed. The means, 25th, 50th, and 75th percentile intervals were calculated for TD, TF, T5F, TF/TD and T5F/TD. For each case the time difference between TD and TF (TD-TF) as well as the time difference between TD and T5F (TD-T5F) were calculated. Wilcoxon signed-rank test was conducted for TD-TF and TD-T5F.

### Accuracy at the time of diagnosis

In addition to the evaluation of early correct disease suggestions, the accuracy of Ada DX at the time of diagnosis was evaluated. To assess the accuracy of Ada DX at the time of diagnosis, the correctness of the number one disease suggestion from the probability disease list was determined for the diagnosis visit. The probability disease list was chosen because with all information that led to sufficient confirmation of clinical diagnosis entered into Ada DX the highest probability suggested condition was expected for that case. In cases with multiple assigned diagnoses, the correctness was defined as the presence of all assigned conditions as consecutive top suggestions in the probability disease list. Mean accuracy and 95% confidence intervals were calculated. Reasons for incorrect disease suggestions were identified and categorized.

### False positive estimation

There was no control group of patients. Instead, the internal set of test cases was used in order to estimate the magnitude of false positive suggestions of the included rare diseases. Out of all the internal test cases, all cases with an internal medicine category that were either cases from medical literature or idealized cases relating to the typical presentation at diagnosis were selected. The cases included common and rare disease cases alike. Selected test cases were evaluated for false positive results of the included rare diseases in top fit and among top 5 fit disease suggestions.

### DDSS optimisation

Ada DX does not yet cover an extensive domain of rare diseases. The purpose of the study was to evaluate Ada DX’s potential for early disease suggestion for future large-scale optimization. Therefore, the evaluation of early correct disease suggestion in a partially optimized system was chosen as the main outcome of this study.

During the course of the study, the Ada DX medical knowledge base and reasoning engine were optimized in several ways. For the cases included in the study, noted missing disease models were created, missing entities to represent confirmatory clinical case evidence were added, and missing medical information was modeled in the medical knowledge base. In addition, independently of this study, changes were implemented due to the general development of the system. All optimizations of the medical knowledge base were performed by medical doctors modeling validated information from peer-reviewed medical literature, in accordance with internal workflows. The extended medical content was regularly updated to Ada DX. Cases for this study were updated in Ada DX after content updates.

### Ethical approval and data processing

Ethical approval was obtained from the ethics committee at the Hannover Medical School. Written consent was obtained from all selected patients. All data was stored and transferred in pseudonymized form. Data processing and transfer were performed in accordance with national and local guidelines. An order data processing agreement was made between the Hannover Medical School and Ada Health.

## Results

113 cases were originally sampled, of which 93 cases were included and 20 were excluded. Exclusion criteria included lack of confirmed diagnosis (4 cases), lack of information about the visit of diagnosis (4), lack of information about course prior to the diagnosis (10), non-rare main diagnosis (1), and missing consent (1). The 93 included cases contain in total 42 different diagnoses. Table 1 shows a summary of the included cases. Table 2 shows a summary of the diagnoses in the included cases. Additional file 1 contains the selected findings for each case.

**Table 1:** Characteristics of included cases

	Female	Male	Total
Number of included cases	58	35	93
Cases with multiple diagnoses	7	5	12
Mean months to clinical diagnosis	24 (5 to 58)	11 (3 to 68)	17 (4 to 65)
Mean number of visits	5.90 (3 to 8)	5.40 (2 to 6)	5.70 (3 to 7)
Mean age at diagnosis in years	43.8 (32 to 56)	45.2 (37 to 56)	44.3 (33 to 56)
Mean age at symptom onset in years	39 (27 to 50)	40 (32 to 50)	40 (27 to 50)

Interquartile ranges in brackets

**Table 2:** Summary of confirmed diagnoses in included cases

Confirmed diagnosis	No. of cases	New disease model
Antiphospholipid syndrome (APS)	2	
Antisynthetase syndrome	5	yes
Behcet’s disease	5	
Chronic hepatitis C	1	
Chronic polyarthritis	2	
CREST syndrome	1	
Cryoglobulinemia	4	
Cryopyrin-associated periodic syndrome (CAPS)	3	yes
Eosinophilic granulomatosis with polyangiitis (EGPA)	2	
Fabry disease	2	
Familial Mediterranean fever (FMF)	4	
Felty syndrome	1	
Focal segmental glomerulosclerosis (FSGS)	1	
Giant cell arteritis	1	
Gout arthritis	2	
Granulomatosis with polyangiitis (GPA)	11	
Henoch-Schonlein purpura (HSP)	3	
Hypophosphatasia	2	yes
IgG4-related disease	4	yes
Kimura disease	1	yes
Mixed amyloidosis	1	
Mixed connective tissue disease (MCTD)	1	
Panarteritis nodosa	2	
Polymyositis/dermatomyositis	2	
Polymyositis/scleroderma overlap	1	yes
Primary sclerosing cholangitis	1	
Relapsing polychondritis	2	
Retroperitoneal fibrosis	1	yes
SAPHO syndrome	3	yes
Sarcoidosis	4	
Sjogren’s syndrome	4	
Small fiber neuropathy	1	yes
Spondyloarthritis	7	
Stickler syndrome	1	yes
Systemic lupus erythematosus (SLE)	5	
Systemic sclerosis	1	
Takayasu’s arteritis	4	
Thromboangiitis obliterans	1	
Thrombotic thrombocytopenic purpura (TTP)	1	
TNF receptor associated periodic syndrome (TRAPS)	1	yes
Tubulointerstitial nephritis and uveitis syndrome (TINU)	2	yes
Whipple disease	1	
Total	93	12

Cases with multiple diagnoses appearing multiple times

### Early correct disease suggestion

Ada DX suggested the correct disease *earlier* than the time of clinical diagnosis among the top 5 fit disease suggestions in 53.8% of cases (50 of 93), and as the top fit disease suggestion in 37.6% of cases (35 of 93). Correct suggestions were consistent throughout the remaining case visits in 46 of 50 (92.0%) correct top 5 fit suggestions and 26 of 35 (74.3%) correct top fit suggestions. The median time advantage of correct disease suggestions compared to the time point of clinical diagnosis was 3 months or 50% for T5F, and 1 month or 21% for TF. The correct diagnosis was suggested at the *first* documented patient visit among the top 5 fit disease suggestions in 33.3% of cases (31 of 93), and as the top fit disease suggestion in 16.1% of cases (15 of 93).

Table 3 shows a comparison of time to diagnosis without using Ada DX and the time to correct disease suggestion using Ada DX.

**Table 3:** Comparison of the original TD without the use of Ada DX and the time to correct disease suggestions with the use of Ada DX

	Percentiles			
	25th	50th	75th	Max
Among all included cases				
Time to clinical diagnosis (TD)	4.0	17.0	65.0	383
Among cases with any correct top suggestion respective top 5 suggestion				
Time to correct top suggestion (TF)	0.3	6.0	31.3	215
Time to correct top 5 suggestion (T5F)	0.0	1.0	14.0	215
TF normalised to TD (TF/TD)	10.2%	79.0%	100%	100%
T5F normalised to TD (T5F/TD)	0.0%	50.0%	100%	100%

All times are expressed in months

**Fig. 2:**
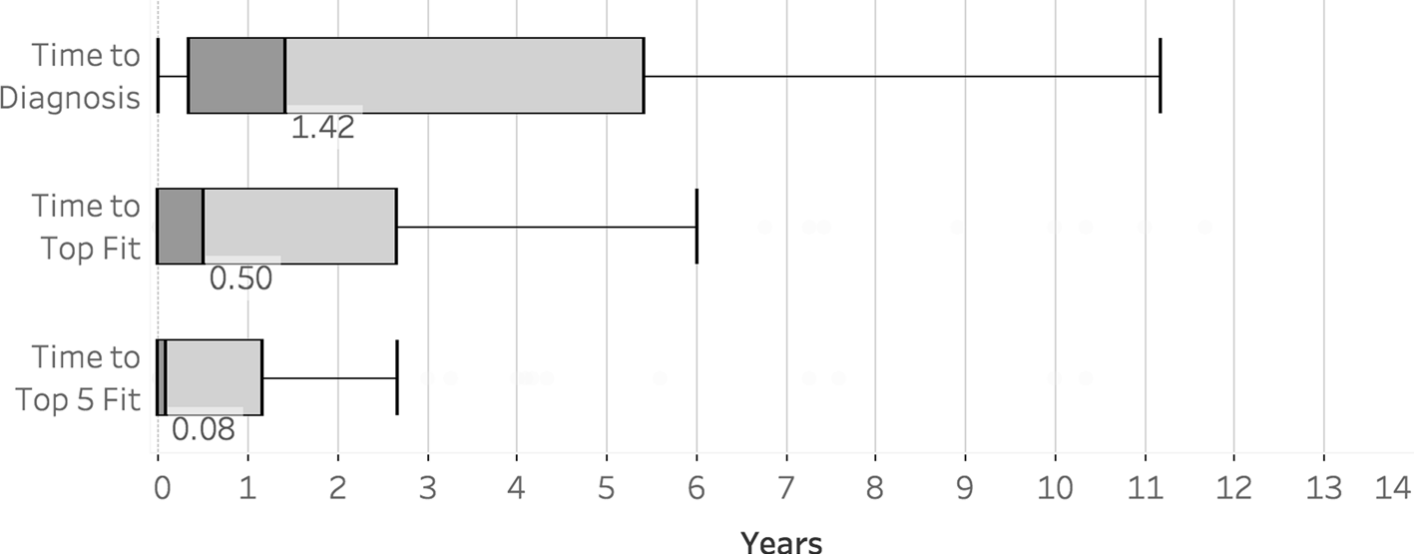
Distribution of TD, TF and T5F. Boxplots for time to clinical diagnosis (TD), time to correct top fit suggestion (TF) and time to correct top 5 fit suggestion (T5F). Outliers outside the whiskers were cut out. Additional information is provided in Table 3

**Fig. 3:**
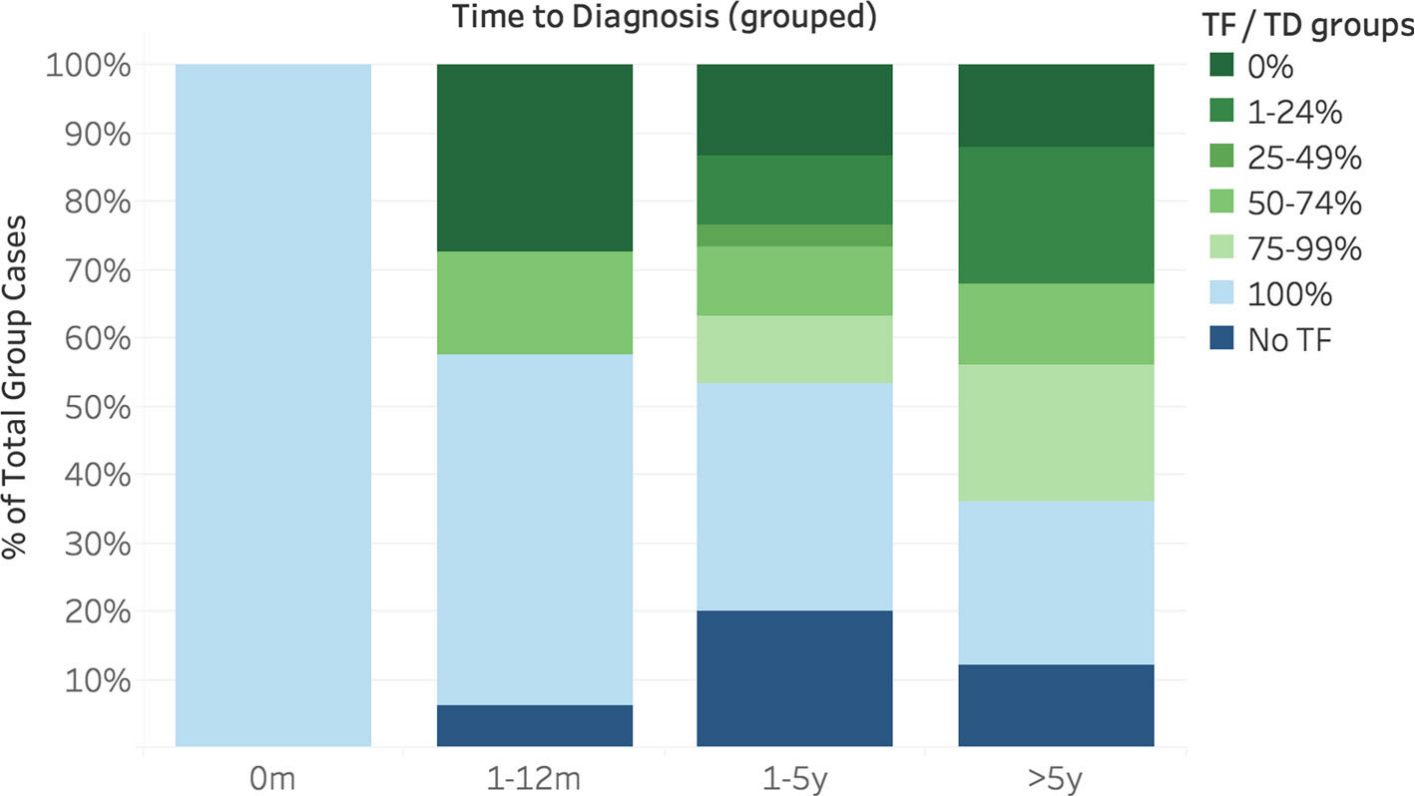
Distribution of TF/TD by TD. Visualisation of TF relative to TD, grouped by TD. Number of cases per group: 0m: 5; 1-12m: 33; 1-5y: 30; >5y: 25

**Fig. 4:**
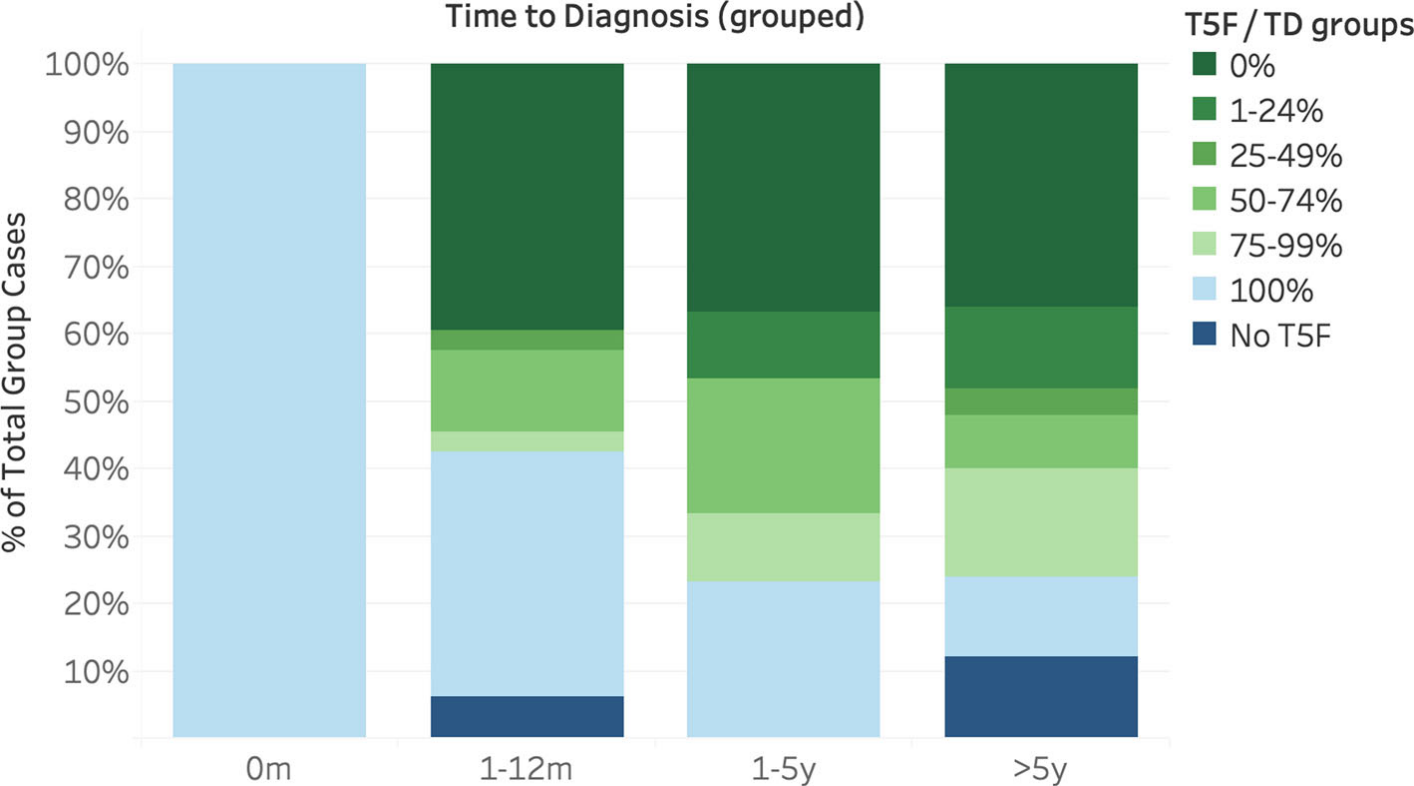
Distribution of T5F/TD by TD. Visualisation of T5F relative to TD, grouped by TD. Number of cases per group: 0m: 5; 1-12m: 33; 1-5y: 30; >5y: 25

Figure 2 shows the distribution of TD, TF and T5F. Figures 3 and 4 show the distribution of TF and T5F normalized to TD and grouped by TD.

Wilcoxon signed-rank test cannot reject the null hypothesis, which means that there is a difference between TD in the medical record and the time to correct disease suggestion for TD-T5F (z-score -6.68, *α*=0.05, *p*-value <0.001) and TD-TF (z-score -5.71, *α*=0.05, *p*-value <0.001).

### Accuracy at the time of diagnosis

The accuracy of top probability suggestions of Ada DX at the time of diagnosis was 89.25% (83 of 93 cases; 95% CI: 82.92 to 95.58%).

### False positive estimation

1,246 internal test cases from the internal medicine category were selected, including 693 cases from medical literature and 553 idealized cases relating to typical presentation at diagnosis. False positive suggestions of the included rare diseases were found in 1.61% for top fit and in 16.7% among top 5 fit disease suggestions.

### Reasons for incorrect suggestion at the time of diagnosis

In the 10 cases with incorrect disease suggestion at the time of diagnosis, a number of characteristics were identified, which are highlighted below.

#### Multiple diagnoses (multimorbidity)

Seven cases with incorrect disease suggestion at the time of diagnosis were characterized by the presence of several diseases in parallel, either as preexisting known diagnoses (2), or as unknown but later confirmed diagnoses (5). In 3 of these cases, Ada DX suggested only a subset of the confirmed diagnoses (one out of two and two out of three, respectively). In the remaining 4 cases, Ada DX was entirely incorrect.

#### Atypical presentation

In 3 cases with an incorrect probability disease suggestion during the simulated diagnosis visit, the symptom constellation was identified as atypical with regard to the confirmed condition. Common features of atypical presentation included presence of very uncommon symptoms, absence of very common symptoms, very uncommon primary site of disease involvement, uncommon age of onset, and uncommon disease time course. Atypical presentation was related to low concordance of case information with the disease model, and subsequent low disease probability in all visits.

### Entities created as a result of the study

Twelve disease models were created and added to the knowledge base as a result of this study. Disease modeling included creation of main symptom models if these were not yet part of the knowledge base. The created disease models are listed in Table 2.

## Discussion

The main purpose of our study was to evaluate disease suggestions provided by Ada DX in rare inflammatory systemic disease cases when optimized for this domain. We conducted a retrospective study of rare disease cases with confirmed diagnoses. We optimized the system’s rare disease knowledge base and assessed the correctness and timing of suggestions. The rare disease suggestions were based on the ranked fit of the symptom constellation for the respective disease models.

Our findings suggest that Ada DX could provide accurate rare disease suggestions earlier than the time of clinical diagnosis in many cases based on information from the medical record, thus likely available to non-rare disease specialists. Our findings further show that, at the time of diagnosis, accurate disease suggestions were provided in most cases. Results pertaining to the system’s accuracy should be interpreted cautiously due to methodological limitations. A prerequisite for this study was the extension and optimization of the system’s medical knowledge base of selected rare diseases and related symptoms.

The interpretation of our results suggests that Ada DX has the potential to highlight the possibility of rare disease to physicians early in the course of a case. Effects on the actual TD in the clinical setting cannot be directly concluded. Evaluation of such effects requires a prospective study. However, we believe that early rare disease suggestions can facilitate earlier diagnosis. An early suggestion of diseases may increase awareness among physicians, particularly of those who may be non-rare disease specialists, thereby reducing diagnostic inaccuracy due to insufficient knowledge or premature closure [8, 32]. Suggesting possible rare diseases can increase the level of early suspicion that is necessary for diagnosis. By delivering early diagnostic support, Ada DX could alleviate the challenges of rare disease diagnosis. Ada DX could serve as a prototype of the tool that NAMSE, the UK Department of Health and other stakeholders in the rare disease community have recommended to develop [11].

Such an endeavor would require a structured and comprehensive extension of Ada’s rare disease knowledge base. Moreover, availability of the tool, preferably via a web-based application, is required to scale for widespread use and to support PCPs and specialists. This would give Ada DX the potential to empower PCPs to improve accurate rare disease referral, provide more accurate rare disease diagnosis, and shorten TD in rare disease cases on a larger scale.

Whether Ada DX disease suggestions effectively help physicians make better decisions in a real-world setting must be further investigated. For example, how will physicians know when to seriously consider a rare disease suggestion and when to ignore it? As a support and reminder system that presents a list of diseases ranked by their estimated probability and fit, Ada DX will necessarily suggest diseases that are not ultimately the correct diagnosis. Such false positive suggestions are not necessarily problematic in a reminder system. Our analysis of false positive suggestions based on a large set of common and rare internal medicine test cases revealed a low false positive rate. We do not know how either correct or false positive disease suggestions will affect the diagnostic process, costs, patient safety, and health outcomes. While DDSSs could modestly increase the risk of unnecessary diagnostic procedures [17, 33] it has the potential to improve overall diagnostic quality and reduce costs [20, 34]. These effects need to be evaluated in future studies.

### Potential improvement of Ada DX

The analysis disclosed several reasons for an inaccurate disease suggestion, which might indicate possible areas of future improvement.

#### Multiple diagnoses (multimorbidity)

The presence of multiple diagnoses in single cases appeared to be among the most challenging scenarios for Ada DX in the given case set. Multiple diagnoses led to a lower accuracy and subsequently a lack of early correct disease suggestion. To increase diagnostic accuracy, the possibility to recognize multimorbidity is an ideal target for improvement.

#### Strict diagnostic criteria

Ada DX does not provide the option for excluding specific disease suggestions by assessment of strict diagnostic criteria, such as those provided in diagnostic guidelines and disease classifications. For that reason, prominent disease suggestions that were based on reasonably high probability estimations, but did not match strict diagnostic criteria, could not be excluded. Although the application of strict criteria partly contradicts the concept of probabilistic reasoning, it is of great importance when making or excluding diagnoses. Integration of strict diagnostic criteria separately from or after the probabilistic inference of disease suggestions, or the possibility to manually exclude suggested conditions, should be considered as an additional feature.

#### Consideration of therapy effects

Information concerning therapy cannot be included in cases. Consequently therapy effects are not reflected in the probability estimation although they can be of diagnostic relevance. Examples include factors such as therapy failure, symptom improvement with therapy, and consideration of medication side effects. The information that is conveyed by therapy failure should also be recognizable.

### Compatibility with existing databases

Regarding the Ada knowledge base and its extension to include specific rare disease knowledge, the importance of system interoperability should not be underestimated and future optimization should prioritize compatibility. Knowledge base compatibility with existing rare disease databases like Orphanet [22] should be emphasized and should at least include disease mapping and codification. The existing Orphanet nomenclature (Orpha numbers) should be represented in the Ada knowledge base. Integration of such external databases would increase disease coverage. Integration with external databases also appears necessary for future improvements of Ada DX for rare disease. Ontology mapping facilitates scientific cooperation and follows recommendations from European institutions such as the UK Strategy for Rare Diseases, the French National Plan on Rare Diseases, and the German National Action League for People with Rare Diseases [10, 11, 35]. Database compatibility might also allow for a more efficient and targeted knowledge base extension because it could enable integration of further related databases, such as existing databases of genetic variants. Connection to available genetic information could be achieved through Online Mendelian Inheritance in Man (OMIM) [23] genetic reference numbers. It would facilitate the integration of known disease genotypes, gene-phenotype relations, as well as the appropriate suggestion and handling of genetic tests in Ada. Since most of such databases also rely on the compatibility with medical phenotype ontologies, especially the HPO [21], coverage of HPO terms should be comprehensively extended. HPO terms should be represented in a way that enables HPO users to use the system seamlessly.

### Future knowledge base extension

To increase the usefulness of DDSS, complete coverage of known rare diseases should be desired. In the face of over 7000 known rare diseases and rapidly increasing medical knowledge, the process of disease model creation should be supported by technological means. A strategy for future disease model creation should aim for curated, automated modeling from structured disease databases. Furthermore, unstructured sources should be mined via the application of natural language processing (NLP). Similar technology could be applied to keep the knowledge base up to date. NLP could be used to screen medical publications to facilitate continuous updates in the knowledge base. Although such a process should still be curated by medical editors and follow rigorous quality testing, it could accelerate the process of knowledge base extension and maintenance.

### User input dependency

Apart from the previously mentioned reasons for inaccurate disease suggestions, correct early disease suggestion implies additional challenges. It should be acknowledged that the capability of Ada DX to provide adequate disease suggestions is highly dependent on appropriate user input. Specifically, Ada DX depends on information gathered by the physician’s history assessment, examination, and further tests that are needed to confirm the correct diagnosis. It is therefore determined by the physician’s knowledge and skills in these areas. Nevertheless, Ada DX facilitates correct data gathering. For example, this can be achieved by not only suggesting diseases but also appropriate diagnostic tests and next steps specific to early diagnosis. While possible effects of diagnostic test suggestion and next step recommendation have not been examined in this study, it can be speculated that improvement of such features might further facilitate early diagnosis.

The manual work to enter cases was significant. If Ada DX was to be routinely used in clinical practice, the user experience must be improved to reduce active effort. If possible, collected data in Ada DX should be integrated into the electronic health record to avoid double data input and manual work for clinicians, ideally operating in the background and adapting to clinicians’ workflows [36].

### Study limitations

A retrospective analysis of confirmed rare disease cases is generally suitable to assess the potential accuracy of DDSSs in such cases. However, a drawback of a retrospective approach is that the results can only be interpreted exploratively. A retrospective approach is generally suspect because of selection bias. Even though this study was partially controlled by fixed inclusion and exclusion criteria, there was inherent selection bias due to the focus on cases with a long course of disease and high final diagnostic certainty. The potential effect on TD might be lower in cases with a shorter course of disease or lower diagnostic certainty. A strength of the study is that a wide range of diagnoses from the group of systemic inflammatory diseases were represented (*n*=42), including cases of co-morbidity. However, results are limited to this group of diseases. Generalization of the results to the entire domain of rare diseases is not appropriate, as only a subgroup of rare diseases was studied. Following a monocentric design, a generalization of the study results applying to other institutions or medical domains is limited.

The unblinded case input and subsequent risk of confirmation bias represent a methodological limitation. While case input was not blinded to diagnosis, it was based on written documented information from the medical records to reduce hindsight bias and retrospective misinterpretation. Given that the study was performed retrospectively on the files of confirmed rare disease cases, the cases’ documented evidence often revealed the diagnosis through confirmatory findings at the time of diagnosis. Blinding of case input to the diagnosis might have been feasible if the confirmatory evidence from the diagnosis visit would not have been transcribed to case summaries. However, excluding evidence from the diagnosis visit would have compromised the evaluation of accuracy at the time of diagnosis. Future studies that aim to validate DDSS accuracy should follow a blinded and prospective design.

Data input was performed by a single user, thus no statement can be given regarding user dependency of the input. While this was accepted in this study because it focused on the potential of the reasoning engine and a first prototype of the Ada DX DDSS, it will be highly relevant to test data entry with different users in practice. For this reason, following studies should put an additional focus on user dependency.

The study was not intended as a validation of the system’s initial diagnostic accuracy (which we know was still limited by the relatively low number of rare diseases covered), but as an explorative estimation of the system’s potential to suggest rare diseases early. For this reason, the optimization of the knowledge base during the course of the study was accepted. This optimization enabled the evaluation of TD in an optimized scenario, but limits the evaluation of the system’s holistic accuracy. We recommend future studies use a fixed knowledge base and reasoning system to validate the accuracy of such systems. To properly evaluate false positive suggestions, a suitable control group should be considered.

The purpose of this study was not to perform a validation of the system’s initial accuracy, so a comparison of two different versions of Ada DX to track developmental changes in the system’s accuracy during optimization was dismissed. With the chosen study design it was not possible to calculate the knowledge base improvement achieved through extension and optimization, which would have required the comparison of two fixed knowledge base instances before and after the study. Nevertheless, such comparison should be considered for future studies that might aim to investigate the effect of a knowledge base extension.

Another limitation is that only confirmed conditions from the case set were added to the knowledge base and not a more extensive set of rare diseases. Arguably, a future extended disease knowledge base might lead to a lower disease suggestion accuracy. Early suggestion ranking of conditions could be lower if more diseases were present in the knowledge base. Specific evidence constellations can be expected to consistently lead to a high ranking of correct disease suggestions. The accuracy at the time of diagnosis should be relatively unaffected by the number of diseases, since specific confirmatory evidence is most likely to be present in the case at that time.

Lastly, effects on TD cannot be measured directly with this study, but the results of early suggestion indicate the potential for earlier suggestion of rare diseases to the physician resulting in earlier correct diagnosis. However, it should be considered that this is not always the case, as such diagnosis might only become legitimate with evolving clinical features in the course of a case.

## Conclusion

The Ada DX DDSS provided accurate rare disease suggestions in most rare disease cases. In many cases, Ada DX provided correct rare disease suggestions early in the course of a disease, sometimes at the very beginning of the patient journey. The interpretation of these results suggests that Ada DX has the potential to correctly suggest rare diseases to physicians early in the course of a case. Whether the use of Ada DX leads to reduced TD in rare diseases in a clinical setting cannot be concluded from this study. Impact on TD should be validated in prospective blinded multicentric studies. Major limiting factors of the system’s disease suggestion accuracy were the presence of multiple diagnoses, complex time-course information, and atypical presentations.

Making accurate rare disease decision support publicly accessible to all doctors in an easy-to-use DDSS could have the potential to effectively reduce TD and improve patient outcomes. Further knowledge base extension should be performed and existing databases should be integrated to provide a tool with complete rare disease coverage and enable further integrations, especially of available genetic databases. Limitations of this study derive from its retrospective and unblinded design, data input by a single user, and the optimization of the knowledge base during the course of the study. Results on the system’s accuracy should thus be interpreted cautiously. Future studies should aim to overcome such limitations by following a blinded design and ideally a prospective approach that is performed on a fixed DDSS version.

## Additional file

Additional file 1 Data supplement. The supplementary data file.pdf contains summaries of the selected findings, attributes and factors for each case, including information from the diagnosis visit and from visits that lead to a correct top fit or top 5 fit suggestion. (PDF 188 kb)
