# Supplementary material for: Can a decision support system accelerate rare disease diagnosis? Evaluating the potential impact of Ada DX in a retrospective study
Source: Orphanet J Rare Dis. 2019 Mar 21;14:69. doi: 10.1186/s13023-019-1040-6 (PMC6427854; doi:10.1186/s13023-019-1040-6)
Supplement: Supplementary file 1 — Data supplement. The supplementary data file.pdf contains summaries of the selected findings, attributes and factors for each case, including information from the diagnosis visit and from visits that lead to a correct top fit or top 5 fit suggestion. (PDF 188 kb) [file 13023_2019_1040_MOESM1_ESM.pdf]

single case supplement

**1**							
**Visit**	Sex	Age	Confirmed Diagnoses	Present Risk Factors	Pathological Symptoms/Findings (with attributes)	Absent Risk Factors	Non-pathological Symptoms/Findings
**Top Five Fit**	Male	45			heat intolerance (time since onset: more than one year)<br>reduced exercise tolerance (time since onset: more than one year)<br>fatigue<br>IgG4 level (result: elevated)<br>dyspepsia/indigestion (time since onset: one week to one month repeat consult: yes)<br>diarrhea (time since onset: one week to one month)<br>malaise (time since onset: one week to one month)<br>myalgia<br>dyspnea (repeat consult: yes time since onset: one month to one year severity: on exertion)<br>flatulence (dairy products: no effect)		
**Diagnosis**	Male	56	IgG4-related disease		heat intolerance (time since onset: more than one year)<br>reduced exercise tolerance (time since onset: more than one year)<br>fatigue<br>dyspepsia/indigestion (time since onset: more than one year repeat consult: yes)<br>malaise (time since onset: more than one year)<br>myalgia<br>dyspnea (repeat consult: yes time since onset: more than one year severity: on exertion)<br>flatulence (dairy products: no effect)<br>constipation (time since onset: more than one year repeat consult: yes)<br>headache (laterality: unilateral throbbing quality: yes repeat consult: yes intensity: severe time since onset: more than one year)<br>impaired vision (time since onset: more than one year)<br>polyuria (time since onset: one month to one year)<br>blood pressure<br>tremor (time since onset: one month to one year)<br>dry throat<br>dry mouth (time since onset: more than one year)<br>cough (time since onset: more than one year repeat consult: yes clear or yellow phlegm: no)<br>IgG4 level (result: elevated)<br>hematuria<br>feeling of heavy legs<br>dermatitis<br>dry skin<br>foreign body sensation in the eye<br>predominance of IgG4 positive plasma cells in histopathology		polydipsia<br>airway obstruction<br>wheezing on auscultation
**2**							
**Visit**	Sex	Age	Confirmed Diagnoses	Present Risk Factors	Pathological Symptoms/Findings (with attributes)	Absent Risk Factors	Non-pathological Symptoms/Findings
**Diagnosis**	Female	63	Sarcoidosis		malaise<br>spots on the knee (color: purple)<br>Lymphadenopathy on palpation<br>high blood pressure<br>headache<br>nausea<br>impaired vision (laterality: bilateral)<br>cough (clear or yellow phlegm: no bloody: no)<br>dyspnea<br>peripheral edema (pitting)<br>spots on the lower extremity (color: purple with elevated margin and central clearing: no with black dots on surface: no scaly surface: no)<br>renal insufficiency<br>bacteria in urine<br>microhematuria<br>leukocyturia<br>nitrituria<br>interstitial nephritis<br>nephrosclerosis<br>renal cysts<br>liver cyst<br>steatosis hepatis<br>hilar lymphadenopathy (laterality: bilateral)<br>mediastinal lymphadenopathy<br>pulmonary radiopacity<br>microalbuminuria		hypercalcemia<br>glomerulonephritis<br>restrictive ventilation pattern<br>mixed pattern in spirometry<br>obstructive ventilation pattern
**3**							
**Visit**	Sex	Age	Confirmed Diagnoses	Present Risk Factors	Pathological Symptoms/Findings (with attributes)	Absent Risk Factors	Non-pathological Symptoms/Findings
**Top Fit, Top Five Fit (<= 1 month early)**	Female	59			spots on the lower extremity (color: purple time since onset: one day to one week)<br>groin pain<br>purpura<br>lower extremity pain (laterality: bilateral)<br>swelling of the lower leg (laterality: bilateral time since onset: one day to one week)<br>fever (time since onset: one day to one week high grade: no)<br>chills (time since onset: one day to one week)		
**Diagnosis**	Female	60	Henoch-Schonlein Purpura (HSP)		spots on the lower extremity (color: purple time since onset: one week to one month)<br>groin pain (time since onset: one week to one month)<br>purpura<br>lower extremity pain (laterality: bilateral time since onset: one week to one month)<br>swelling of the lower leg (laterality: bilateral time since onset: one week to one month)<br>urinary tract infection<br>leukocytoclastic vasculitis		antinuclear antibody level, elevated<br>antineutrophil cytoplasmic antibodies present<br>perinuclear anti-neutrophil cytoplasmic antibodies present<br>cytoplasmic antineutrophil cytoplasmic antibodies present<br>complement C4 level<br>complement C3 level<br>serum complement level<br>elevated rheumatoid factor<br>free T3<br>free T4<br>thyroglobulin antibodies<br>thyroid peroxidase antibodies<br>hepatitis C virus<br>deep vein thrombosis
**4**							
**Visit**	Sex	Age	Confirmed Diagnoses	Present Risk Factors	Pathological Symptoms/Findings (with attributes)	Absent Risk Factors	Non-pathological Symptoms/Findings
**Top Five Fit**	Female	42			sinusitis<br>pulmonary nodules		
**Top Fit**	Female	53			nasal polyp<br>sinusitis<br>eosinophilia in blood		
**Diagnosis**	Female	57	Eosinophilic Granulomatosis with Polyangiitis (EGPA)		dyspnea (severity: daily activities)<br>pulmonary nodules<br>eosinophils in bronchoalveolar lavage<br>Total IgE level, elevated<br>eosinophilia in blood<br>anti-cyclic citrullinated protein antibodies		malignant cells in pulmonary tissue<br>perinuclear anti-neutrophil cytoplasmic antibodies present<br>cytoplasmic antineutrophil cytoplasmic antibodies present
**5**							
**Visit**	Sex	Age	Confirmed Diagnoses	Present Risk Factors	Pathological Symptoms/Findings (with attributes)	Absent Risk Factors	Non-pathological Symptoms/Findings
**Diagnosis**	Female	32	Small Fiber Neuropathy<br>ENaC/CFTR channelopathy		dyspepsia/indigestion<br>painful dysesthesia of the hand (distribution: symmetrical)<br>painful dysesthesia of the foot (distribution: symmetrical)<br>arthralgia<br>neutropenia<br>lymphocytosis<br>creatine kinase level, elevated<br>muscular atrophy of the lower limb<br>diminished deep tendon reflexes<br>vitamin B12 level (result: reduced)<br>myopia<br>double vision<br>airway obstruction<br>hyperventilation<br>painful dysesthesia<br>steatorrhea<br>dry skin		erythrocyte sedimentation rate<br>CRP, elevated<br>antinuclear antibody level, elevated<br>anti-cyclic citrullinated protein antibodies<br>IgG level<br>IgA level<br>IgM level<br>pyramidal signs<br>pathological visual evoked potentials<br>pathological acoustic evoked potentials<br>helicobacter pylori in stomach/duodenum<br>alpha-1 antitrypsin level, decreased<br>fecal calprotectin, elevated<br>CFTR gene mutation
**6**							
**Visit**	Sex	Age	Confirmed Diagnoses	Present Risk Factors	Pathological Symptoms/Findings (with attributes)	Absent Risk Factors	Non-pathological Symptoms/Findings
**Diagnosis**	Male	25	Granulomatosis with Polyangiitis (GPA)		swelling, ankle joint (laterality: unilateral time since onset: one month to one year)<br>blue skin, foot (time since onset: one month to one year laterality: unilateral)<br>ear swelling (bilateral distribution: yes)<br>glomerular filtration rate, reduced<br>serum creatinine level (result: elevated)<br>proteinuria<br>hematuria<br>dysmorphic RBCs in urine<br>cytoplasmic antineutrophil cytoplasmic antibodies present<br>proteinase 3 antibody titer, elevated<br>CRP, elevated<br>anemia<br>alanine transaminase level, elevated<br>aspartate transaminase level, elevated<br>swelling of the nasal and sinus mucosa		pulmonary nodules<br>pulmonary infiltrates<br>pulmonary cavitation
**7**							
**Visit**	Sex	Age	Confirmed Diagnoses	Present Risk Factors	Pathological Symptoms/Findings (with attributes)	Absent Risk Factors	Non-pathological Symptoms/Findings
**Diagnosis**	Male	22	Granulomatosis with Polyangiitis (GPA)		sensory deficit, lower leg (distribution: bilateral, symmetrical time since onset: one month to one year)<br>lower extremity pain (laterality: bilateral time since onset: one month to one year)<br>peripheral polyneuropathy<br>lower extremity paresis (laterality: bilateral time since onset: one month to one year)<br>hearing deficit (laterality: unilateral time since onset: one month to one year)<br>rhinitis<br>gait impairment (time since onset: one month to one year)<br>pulmonary cavitation<br>cytoplasmic antineutrophil cytoplasmic antibodies present<br>proteinase 3 antibody titer, elevated<br>steatosis hepatis<br>hepatomegaly<br>liver cyst<br>splenomegaly<br>splenic mass seen on imaging<br>pericardial effusion<br>insomnia (time since onset: one month to one year)		
**8**							
**Visit**	Sex	Age	Confirmed Diagnoses	Present Risk Factors	Pathological Symptoms/Findings (with attributes)	Absent Risk Factors	Non-pathological Symptoms/Findings

**Top Five Fit**	Male	51			cough (time since onset: more than one year repeat consult: yes bloody: yes)<br>pulmonary radiopacity<br>chest pain (position: retrosternal sharp: no burning: no exertion: exacerbates)<br>atrial fibrillation<br>tachycardia on ECG<br>serum cardiac troponin level, elevated<br>CRP, elevated<br>leukocytosis<br>fever (high grade: no)<br>urinary urgency<br>episcleritis<br>pulmonary nodules<br>pleural effusion		mycobacterium tuberculosis in lower respiratory tract<br>legionella pneumophila in lower respiratory tract<br>fungi in lower respiratory tract<br>bronchiectasis
**Top Fit**	Male	53			cough (time since onset: more than one year repeat consult: yes bloody: yes)<br>pulmonary radiopacity<br>atrial fibrillation<br>tachycardia on ECG<br>CRP, elevated<br>leukocytosis<br>fever (high grade: no time since onset: more than one year)<br>urinary urgency<br>episcleritis<br>pulmonary nodules<br>pleural effusion<br>sinusitis		mycobacterium tuberculosis in lower respiratory tract<br>legionella pneumophila in lower respiratory tract<br>fungi in lower respiratory tract<br>bronchiectasis
**Diagnosis**	Male	54	Granulomatosis with Polyangiitis (GPA)		cough (time since onset: more than one year repeat consult: yes bloody: yes)<br>pulmonary radiopacity<br>hemoptysis (time since onset: more than one year)<br>CRP, elevated<br>leukocytosis<br>fever (high grade: no)<br>urinary urgency<br>episcleritis<br>pulmonary nodules<br>hemoglobin level (result: reduced)<br>sinusitis		mycobacterium tuberculosis in lower respiratory tract<br>legionella pneumophila in lower respiratory tract<br>fungi in lower respiratory tract<br>bronchiectasis
**9**							
**Visit**	Sex	Age	Confirmed Diagnoses	Present Risk Factors	Pathological Symptoms/Findings (with attributes)	Absent Risk Factors	Non-pathological Symptoms/Findings
**Diagnosis**	Female	34	Takayasu's arteritis		fatigue<br>CRP, elevated<br>erythrocyte sedimentation rate (result: elevated)<br>circumferential thickening of the carotid artery wall<br>anterior neck pain (position: lateral time since onset: one month to one year)<br>cervical lymphadenopathy<br>vasculitis		diarrhea<br>constipation<br>abdominal pain<br>dyspepsia/indigestion<br>abdominal mass seen on imaging<br>ascites<br>splenomegaly<br>hepatomegaly<br>cholecystitis<br>abnormal bile duct<br>gallstone<br>carotid bruit<br>stenosis of the carotid artery<br>obstruction of subclavian veins<br>aortitis<br>stenosis of the ascending aorta<br>arterial aneurysm of the thoracic aorta<br>arterial aneurysm of the abdominal aorta<br>stenosis of the renal artery
**10**							
**Visit**	Sex	Age	Confirmed Diagnoses	Present Risk Factors	Pathological Symptoms/Findings (with attributes)	Absent Risk Factors	Non-pathological Symptoms/Findings
**Top Five Fit, Top Fit (<= 1 month early)**	Female	70			reduced exercise tolerance (time since onset: one month to one year)<br>headache<br>facial pain<br>erythrocyte sedimentation rate (result: elevated)<br>leukocyte level (result: elevated)<br>jaw claudication		
**Diagnosis**	Female	70	Giant Cell Arteritis		reduced exercise tolerance (time since onset: one month to one year)<br>headache (time since onset: one month to one year intensity: severe)<br>jaw claudication<br>facial pain<br>erythrocyte sedimentation rate (result: elevated)<br>leukocyte level (result: elevated)<br>syncope<br>temporary loss of consciousness<br>nausea<br>vomiting<br>malaise<br>facial pallor<br>weight loss<br>hyperhidrosis, generalized<br>chills<br>multinucleated giant cells		fever
**11**							
**Visit**	Sex	Age	Confirmed Diagnoses	Present Risk Factors	Pathological Symptoms/Findings (with attributes)	Absent Risk Factors	Non-pathological Symptoms/Findings
**Top Five Fit**	Female	27			feeling of heavy legs (time since onset: one month to one year)<br>epistaxis (time since onset: one month to one year)<br>fever (time since onset: one month to one year)<br>malaise (time since onset: one month to one year repeat consult: yes)<br>myalgia (time since onset: one month to one year)<br>hearing deficit (laterality: unilateral time since onset: one month to one year)		
**Top Fit (<= 1 month early)**	Female	27			feeling of heavy legs (time since onset: one month to one year)<br>epistaxis (time since onset: one month to one year)<br>fever (time since onset: one month to one year)<br>malaise (time since onset: one month to one year repeat consult: yes)<br>myalgia (time since onset: one month to one year)<br>hearing deficit (laterality: bilateral time since onset: one month to one year)<br>otalgia<br>lower extremity pain (laterality: bilateral)<br>gait impairment<br>fatigue		
**Diagnosis**	Female	27	Granulomatosis with Polyangiitis (GPA)		feeling of heavy legs (time since onset: one month to one year)<br>epistaxis (time since onset: one month to one year)<br>fever (time since onset: one month to one year)<br>malaise (time since onset: one month to one year repeat consult: yes)<br>myalgia (time since onset: one month to one year)<br>hearing deficit (laterality: bilateral time since onset: one month to one year)<br>otalgia<br>lower extremity pain (laterality: bilateral)<br>gait impairment<br>fatigue<br>CRP, elevated<br>cytoplasmic antineutrophil cytoplasmic antibodies present<br>proteinase 3 antibody titer, elevated		perinuclear anti-neutrophil cytoplasmic antibodies present
**12**							
**Visit**	Sex	Age	Confirmed Diagnoses	Present Risk Factors	Pathological Symptoms/Findings (with attributes)	Absent Risk Factors	Non-pathological Symptoms/Findings
**Top Five Fit, Top Fit**	Female	37			necrosis of the toes<br>peripheral hypoperfusion<br>peripheral cyanosis<br>generalized arthralgia<br>peripheral polyneuropathy<br>otitis media		
**Diagnosis**	Female	37	Granulomatosis with Polyangiitis (GPA)		otitis media<br>necrosis of the toes (time since onset: one month to one year)<br>peripheral hypoperfusion<br>peripheral cyanosis (time since onset: one month to one year)<br>generalized arthralgia (time since onset: one month to one year)<br>peripheral polyneuropathy<br>pleural effusion<br>pericardial effusion		
**13**							
**Visit**	Sex	Age	Confirmed Diagnoses	Present Risk Factors	Pathological Symptoms/Findings (with attributes)	Absent Risk Factors	Non-pathological Symptoms/Findings
**Top Five Fit, Top Fit (<= 1 month early)**	Female	60			ankle pain (laterality: unilateral time since onset: more than one year)<br>hyperpigmentation of the lower extremity<br>CRP, elevated<br>leukocytosis<br>lymphocytosis<br>periosteal thickening<br>fever (time since onset: more than one year)<br>cough (time since onset: more than one year)<br>cytoplasmic antineutrophil cytoplasmic antibodies present<br>pulmonary nodules<br>thrombocytosis<br>normocytic anemia<br>mean corpuscular hemoglobin (result: reduced)<br>unexplained fracture<br>tricuspid valve regurgitation on imaging<br>alkaline phosphatase level (result: elevated)<br>erythrocyte sedimentation rate (result: elevated)<br>gamma gt level, elevated<br>alanine transaminase level, elevated<br>IgA level (result: elevated)	alcohol abuse	pneumonia<br>endocarditis<br>hepatitis C virus<br>Hepatitis B virus<br>Lyme-specific IgM level, elevated<br>Lyme-specific IgG level, elevated<br>antinuclear antibody level, elevated<br>anti-Sm antibodies<br>anti-dsDNA antibodies<br>anticardiolipin antibodies, elevated<br>pulmonary infiltrates<br>aortitis<br>iron level<br>pleural effusion<br>hepatic mass seen on imaging<br>liver cirrhosis<br>hepatomegaly<br>liver abscess

**Diagnosis**	Female	60	Granulomatosis with Polyangiitis (GPA)		ankle pain (laterality: unilateral time since onset: more than one year)<br>hyperpigmentation of the lower extremity<br>CRP, elevated<br>leukocytosis<br>lymphocytosis<br>periosteal thickening<br>fever (time since onset: more than one year)<br>cough (time since onset: more than one year)<br>cytoplasmic antineutrophil cytoplasmic antibodies present<br>pulmonary nodules<br>thrombocytosis<br>normocytic anemia<br>mean corpuscular hemoglobin (result: reduced)<br>unexplained fracture<br>tricuspid valve regurgitation on imaging<br>alkaline phosphatase level (result: elevated)<br>erythrocyte sedimentation rate (result: elevated)<br>gamma gt level, elevated<br>alanine transaminase level, elevated<br>saddle nose deformity<br>myalgia<br>proteinase 3 antibody titer, elevated	alcohol abuse	pneumonia<br>endocarditis<br>hepatitis C virus<br>Hepatitis B virus<br>Lyme-specific IgM level, elevated<br>Lyme-specific IgG level, elevated<br>antinuclear antibody level, elevated<br>anti-Sm antibodies<br>anti-dsDNA antibodies<br>anticardiolipin antibodies, elevated<br>pulmonary infiltrates<br>aortitis<br>iron level<br>pleural effusion<br>hepatic mass seen on imaging<br>liver cirrhosis<br>hepatomegaly<br>liver abscess
**14**							
**Visit**	Sex	Age	Confirmed Diagnoses	Present Risk Factors	Pathological Symptoms/Findings (with attributes)	Absent Risk Factors	Non-pathological Symptoms/Findings
**Diagnosis**	Male	56	Granulomatosis with Polyangiitis (GPA)		localized arthralgia (laterality: bilateral)<br>epistaxis (time since onset: one month to one year)<br>nasal discharge (time since onset: one month to one year appearance: crusty)<br>microhematuria<br>antineutrophil cytoplasmic antibodies present<br>influenza A virus subtype H1N1 in respiratory tract<br>pulmonary infiltrates<br>CRP, elevated<br>glomerular filtration rate, reduced<br>hemoptysis		serum creatinine level
**15**							
**Visit**	Sex	Age	Confirmed Diagnoses	Present Risk Factors	Pathological Symptoms/Findings (with attributes)	Absent Risk Factors	Non-pathological Symptoms/Findings
**Top Five Fit**	Female	18			reduced exercise tolerance<br>sinus pain (time since onset: one month to one year)		
**Top Fit**	Female	19			reduced exercise tolerance<br>sinus pain (time since onset: one month to one year)<br>otitis media		
**Diagnosis**	Female	19	Granulomatosis with Polyangiitis (GPA)		reduced exercise tolerance<br>sinus pain (time since onset: more than one year)<br>otitis media<br>headache (intensity: severe)<br>knee pain (laterality: bilateral)<br>elbow pain (laterality: bilateral)<br>fever		
**16**							
**Visit**	Sex	Age	Confirmed Diagnoses	Present Risk Factors	Pathological Symptoms/Findings (with attributes)	Absent Risk Factors	Non-pathological Symptoms/Findings
**Top Five Fit**	Female	26			elbow pain (intensity: severe time since onset: one month to one year)<br>shoulder pain (intensity: severe time since onset: one month to one year)<br>knee pain (intensity: severe time since onset: one month to one year)<br>nasal discharge (time since onset: one month to one year)<br>sinus pain (time since onset: one month to one year)		epistaxis
**Diagnosis**	Female	26	Granulomatosis with Polyangiitis (GPA)		elbow pain (intensity: severe time since onset: one month to one year)<br>shoulder pain (intensity: severe time since onset: one month to one year)<br>knee pain (intensity: severe time since onset: one month to one year)<br>nasal discharge (time since onset: one month to one year)<br>sinus pain (time since onset: one month to one year)<br>fever (time since onset: one month to one year)<br>reduced exercise tolerance (time since onset: one month to one year)<br>conjunctivitis (distribution pattern: bilateral)<br>cough (bloody: yes clear or yellow phlegm: yes)<br>pulmonary hemorrhage<br>renal insufficiency<br>oliguria<br>glomerulonephritis		epistaxis<br>dyspnea<br>microhematuria
**17**							
**Visit**	Sex	Age	Confirmed Diagnoses	Present Risk Factors	Pathological Symptoms/Findings (with attributes)	Absent Risk Factors	Non-pathological Symptoms/Findings
**Diagnosis**	Male	50	Granulomatosis with Polyangiitis (GPA)		rhinitis<br>hemoptysis<br>headache<br>knee pain (laterality: bilateral)<br>ankle pain (laterality: bilateral)<br>wrist pain (laterality: bilateral)<br>high blood pressure<br>generalized arthralgia<br>hearing deficit (laterality: unilateral)		
**18**							
**Visit**	Sex	Age	Confirmed Diagnoses	Present Risk Factors	Pathological Symptoms/Findings (with attributes)	Absent Risk Factors	Non-pathological Symptoms/Findings
**Diagnosis**	Female	45	Cryoglobulinemia		recurrent respiratory tract infections<br>leukocytoclastic vasculitis<br>pathologically increased cryoglobulin level<br>IgM monoclonal antibody<br>mesangiocapillary glomerulonephritis		axillary lymphadenopathy<br>cervical lymphadenopathy<br>supraclavicular lymphadenopathy<br>lower airway obstruction
**19**							
**Visit**	Sex	Age	Confirmed Diagnoses	Present Risk Factors	Pathological Symptoms/Findings (with attributes)	Absent Risk Factors	Non-pathological Symptoms/Findings
**Diagnosis**	Male	57	Cryoglobulinemia		reduced exercise tolerance<br>dyspnea<br>headache (intensity: severe)<br>nausea<br>abdominal pain<br>high blood pressure<br>pleural effusion<br>generalized lymphadenopathy<br>splenomegaly<br>renal insufficiency<br>proteinuria<br>metabolic acidosis<br>serum creatinine level (result: elevated)<br>serum albumin level (result: reduced)<br>CRP, elevated<br>BNP in serum, elevated<br>d-dimer level in blood, elevated<br>hematuria<br>complement C3 level (result: reduced)<br>complement C4 level (result: reduced)<br>IgG level (result: reduced)<br>beta 2 microglobulin level, elevated<br>vascular markings of the lung (result: elevated)<br>lobar consolidation<br>pulmonary infiltrates (laterality: bilateral)<br>swelling, ankle joint (laterality: bilateral)<br>ankle pain (laterality: bilateral)<br>effusion, ankle<br>purpura<br>nephrotic syndrome<br>mesangiocapillary glomerulonephritis<br>pathologically increased cryoglobulin level		antinuclear antibody level, elevated<br>cytoplasmic antineutrophil cytoplasmic antibodies present<br>perinuclear anti-neutrophil cytoplasmic antibodies present<br>anti-GBM antibodies present<br>Bence-Jones protein
**20**							
**Visit**	Sex	Age	Confirmed Diagnoses	Present Risk Factors	Pathological Symptoms/Findings (with attributes)	Absent Risk Factors	Non-pathological Symptoms/Findings
**Top Five Fit, Top Fit**	Female	50			reduced exercise tolerance<br>fatigue<br>fever<br>knee pain (laterality: unilateral)<br>CRP, elevated<br>general muscle weakness<br>toe pain (laterality: bilateral)		
**Diagnosis**	Female	52	Antisynthetase Syndrome		reduced exercise tolerance (time since onset: more than one year)<br>fatigue<br>fever (time since onset: more than one year)<br>knee pain (laterality: unilateral time since onset: more than one year)<br>CRP, elevated<br>lymphocytosis<br>swelling of the foot (laterality: unilateral time since onset: more than one year)<br>dyspnea (severity: on exertion time since onset: more than one year repeat consult: yes)<br>air-fluid levels in the sinus<br>cough (clear or yellow phlegm: no time since onset: more than one year repeat consult: yes)<br>weight loss<br>malaise (time since onset: more than one year)<br>sinus pain (time since onset: more than one year)<br>swelling, ankle joint (laterality: unilateral time since onset: more than one year)<br>peripheral edema (pitting) (time since onset: more than one year)<br>lymphocytosis in bronchoalveolar lavage<br>mitral valve regurgitation on imaging<br>high blood pressure (time since onset: more than one year)<br>enlarged thyroid gland<br>CD4+ to CD8+ ratio (result: elevated)<br>ground-glass opacities<br>vascular markings of the lung (result: elevated)<br>swelling of the nasal and sinus mucosa<br>hyperhidrosis, generalized (night sweats: yes time since onset: more than one year)<br>general muscle weakness (time since onset: more than one year)<br>antinuclear antibody level, elevated<br>anti-SRP antibodies<br>toe pain (laterality: bilateral time since onset: more than one year)		pulmonary infiltrates<br>pulmonary embolism<br>anti-Sm antibodies<br>anti-U1-RNP antibodies<br>anti-SSA autoantibodies<br>anti-La (SS-B)-autoantibodies<br>anti-Scl-70<br>anti-Jo 1 antibodies<br>glomerular filtration rate, reduced<br>pulmonary nodules<br>pleural effusion<br>hilar lymphadenopathy<br>mediastinal lymphadenopathy
**21**							
**Visit**	Sex	Age	Confirmed Diagnoses	Present Risk Factors	Pathological Symptoms/Findings (with attributes)	Absent Risk Factors	Non-pathological Symptoms/Findings

**Top Five Fit**	Female	58		diabetes mellitus	reduced exercise tolerance (time since onset: more than one year)<br>dyspnea (severity: daily activities time since onset: one month to one year)<br>foot pain (laterality: unilateral time since onset: one month to one year)<br>diffusion capacity (result: reduced)<br>leukopenia<br>dizziness (time since onset: one month to one year)<br>weight loss<br>creatine kinase level, elevated		arthralgia<br>headache
**Top Fit**	Female	59		diabetes mellitus	reduced exercise tolerance (time since onset: more than one year)<br>foot pain (laterality: unilateral time since onset: one month to one year)<br>diffusion capacity (result: reduced)<br>leukopenia<br>dizziness (time since onset: one month to one year)<br>weight loss<br>creatine kinase level, elevated<br>ground-glass opacities<br>pulmonary fibrosis<br>dyspnea (time since onset: one month to one year)		arthralgia<br>headache
**Diagnosis**	Female	60	Antisynthetase Syndrome	diabetes mellitus	reduced exercise tolerance (time since onset: more than one year)<br>dyspnea (severity: daily activities time since onset: one month to one year)<br>foot pain (laterality: unilateral time since onset: one month to one year)<br>diffusion capacity (result: reduced)<br>leukopenia<br>dizziness (time since onset: one month to one year)<br>weight loss<br>creatine kinase level, elevated<br>ground-glass opacities<br>pulmonary fibrosis<br>anti-PL 7 antibodies		arthralgia<br>headache
**22**							
**Visit**	Sex	Age	Confirmed Diagnoses	Present Risk Factors	Pathological Symptoms/Findings (with attributes)	Absent Risk Factors	Non-pathological Symptoms/Findings
**Top Five Fit, Top Fit**	Male	41			fever (time since onset: one month to one year high grade: no)<br>CRP, elevated<br>sinusitis<br>leukocytosis<br>leukocyte 'left shift'<br>dyspnea<br>chest pain (respiration: exacerbates)<br>erythema nodosum<br>retinitis<br>aphthous ulcerations of the oral cavity<br>erythrocyte sedimentation rate (result: elevated)<br>gamma gt level, elevated<br>alkaline phosphatase level (result: elevated)<br>alpha 2 globulin level (result: elevated)<br>swelling of the nasal and sinus mucosa<br>cervical lymphadenopathy<br>mediastinal lymphadenopathy		antinuclear antibody level, elevated<br>anti-dsDNA antibodies<br>hilar lymphadenopathy<br>pulmonary radiopacity<br>pulmonary infiltrates
**Diagnosis**	Male	48	Behcet's disease		fever (time since onset: more than one year high grade: no)<br>CRP, elevated<br>sinusitis<br>leukocytosis<br>leukocyte 'left shift'<br>erythema nodosum<br>retinitis<br>aphthous ulcerations of the oral cavity<br>erythrocyte sedimentation rate (result: elevated)<br>gamma gt level, elevated<br>alkaline phosphatase level (result: elevated)<br>alpha 2 globulin level (result: elevated)<br>swelling of the nasal and sinus mucosa<br>cervical lymphadenopathy<br>mediastinal lymphadenopathy<br>thrombocytosis<br>swelling, knee		antinuclear antibody level, elevated<br>anti-dsDNA antibodies<br>hilar lymphadenopathy<br>pulmonary radiopacity<br>pulmonary infiltrates
**23**							
**Visit**	Sex	Age	Confirmed Diagnoses	Present Risk Factors	Pathological Symptoms/Findings (with attributes)	Absent Risk Factors	Non-pathological Symptoms/Findings
**Diagnosis**	Male	67	Polymyositis<br>Primary sclerosing cholangitis		creatine kinase level, elevated<br>myalgia (time since onset: one month to one year)<br>upper extremity paresis (time since onset: one month to one year)<br>proteinuria (intensity: moderate to severe)<br>serum creatinine level (result: elevated)<br>glomerular filtration rate, reduced<br>antinuclear antibody level, elevated<br>atrial fibrillation<br>left axis deviation<br>left fascicular hemiblock<br>serum cardiac troponin level, elevated<br>stenosis of the arteries of the leg<br>stenosis of the coronary artery<br>muscle necrosis<br>steatosis hepatis<br>aspartate transaminase level, elevated<br>alanine transaminase level, elevated<br>gamma gt level, elevated<br>antimitochondrial antibody<br>arrhythmias on ECG<br>BNP in serum, elevated<br>proximal lower extremity paresis (time since onset: one month to one year)	diabetes mellitus	cardiac CK-MB level, elevated<br>anticardiolipin antibodies, elevated<br>anti-dsDNA antibodies
**24**							
**Visit**	Sex	Age	Confirmed Diagnoses	Present Risk Factors	Pathological Symptoms/Findings (with attributes)	Absent Risk Factors	Non-pathological Symptoms/Findings
**Top Five Fit**	Female	28			petechiae<br>headache<br>bad taste in mouth<br>malaise		chills<br>pruritus<br>sinus pain
**Top Fit**	Female	28			petechiae<br>headache<br>bad taste in mouth<br>malaise		chills<br>pruritus<br>hemoglobin level<br>platelet count<br>antinuclear antibody level, elevated<br>elevated rheumatoid factor<br>leukocyte level<br>sinus pain
**Diagnosis**	Female	31	Henoch-Schonlein Purpura (HSP)		petechiae<br>headache<br>bad taste in mouth<br>malaise<br>mesangiocapillary glomerulonephritis		chills<br>pruritus<br>hemoglobin level<br>platelet count<br>antinuclear antibody level, elevated<br>elevated rheumatoid factor<br>leukocyte level<br>sinus pain
**25**							
**Visit**	Sex	Age	Confirmed Diagnoses	Present Risk Factors	Pathological Symptoms/Findings (with attributes)	Absent Risk Factors	Non-pathological Symptoms/Findings
**Diagnosis**	Female	17	Systemic Lupus Erythematosus (SLE)		malaise<br>reduced exercise tolerance<br>antinuclear antibody level, elevated<br>anti-dsDNA antibodies<br>serum complement level (result: reduced)<br>nephrotic syndrome<br>proteinuria (intensity: moderate to severe)<br>serum creatinine level (result: elevated)<br>pericardial effusion<br>polyserositis<br>pericarditis<br>anemia<br>renal insufficiency		
**26**							
**Visit**	Sex	Age	Confirmed Diagnoses	Present Risk Factors	Pathological Symptoms/Findings (with attributes)	Absent Risk Factors	Non-pathological Symptoms/Findings
**Top Five Fit, Top Fit (<= 1 month early)**	Female	21		Hx: recent infection	fever (high grade: yes)<br>myalgia<br>abdominal pain (position: epigastric)<br>fatigue<br>arthralgia<br>reduced exercise tolerance<br>diarrhea<br>proteinuria<br>pancytopenia<br>splenomegaly<br>fluid in recto-uterine pouch<br>peritonism<br>proof of acute pancreatitis<br>volume depletion		
**Diagnosis**	Female	21	Systemic Lupus Erythematosus (SLE)	Hx: recent infection	fever (high grade: yes)<br>myalgia<br>abdominal pain (position: epigastric)<br>fatigue<br>arthralgia<br>reduced exercise tolerance<br>diarrhea<br>proteinuria<br>pancytopenia<br>splenomegaly<br>fluid in recto-uterine pouch<br>peritonism<br>proof of acute pancreatitis<br>volume depletion<br>general muscle weakness<br>ascites<br>anti-dsDNA antibodies<br>anti-Sm antibodies<br>creatine kinase level, elevated<br>lactate dehydrogenase level, elevated		
**27**							
**Visit**	Sex	Age	Confirmed Diagnoses	Present Risk Factors	Pathological Symptoms/Findings (with attributes)	Absent Risk Factors	Non-pathological Symptoms/Findings

**Top Five Fit**	Female	21			tricolor pattern of discoloration of the fingers and toes (time since onset: one month to one year)<br>foamy urine		
**Diagnosis**	Female	24	Systemic Lupus Erythematosus (SLE)<br>Mixed connective tissue disease (MCTD)		tricolor pattern of discoloration of the fingers and toes (time since onset: more than one year)<br>foamy urine (time since onset: one month to one year)<br>peripheral edema (pitting) (time since onset: one month to one year)<br>dyspnea (severity: daily activities)<br>amenorrhea<br>dysphagia<br>glomerular filtration rate, reduced<br>glomerulonephritis<br>anti-U1-RNP antibodies<br>anti-Sm antibodies<br>anti-dsDNA antibodies<br>partial thromboplastin time (result: prolonged)<br>hematuria<br>proteinuria<br>renal insufficiency<br>microcytic anemia<br>arthritis<br>polyserositis<br>hepatomegaly<br>splenomegaly<br>cachexia<br>anti-SSA autoantibodies		
**28**							
**Visit**	Sex	Age	Confirmed Diagnoses	Present Risk Factors	Pathological Symptoms/Findings (with attributes)	Absent Risk Factors	Non-pathological Symptoms/Findings
**Diagnosis**	Female	39	Sjogren's syndrome<br>Systemic Lupus Erythematosus (SLE)		leukopenia<br>hemoglobin level (result: reduced)<br>dry mouth<br>dry eye<br>diarrhea<br>anti-alpha-fodrin antibodies<br>missed menstruation		
**29**							
**Visit**	Sex	Age	Confirmed Diagnoses	Present Risk Factors	Pathological Symptoms/Findings (with attributes)	Absent Risk Factors	Non-pathological Symptoms/Findings
**Top Five Fit**	Male	35			epistaxis (time since onset: one month to one year repeat consult: yes)<br>sneezing (time since onset: one month to one year)<br>swelling of the nasal and sinus mucosa<br>elbow pain (laterality: bilateral)<br>knee pain (laterality: bilateral)<br>ankle pain (laterality: bilateral)		
**Diagnosis**	Male	36	Granulomatosis with Polyangiitis (GPA)		epistaxis (time since onset: more than one year repeat consult: yes)<br>sneezing (time since onset: one month to one year)<br>swelling of the nasal and sinus mucosa<br>elbow pain (time since onset: one month to one year laterality: bilateral)<br>knee pain (time since onset: one month to one year laterality: bilateral)<br>ankle pain (time since onset: one month to one year laterality: bilateral)<br>nasal discharge (appearance: crusty time since onset: one month to one year)<br>hemoptysis		
**30**							
**Visit**	Sex	Age	Confirmed Diagnoses	Present Risk Factors	Pathological Symptoms/Findings (with attributes)	Absent Risk Factors	Non-pathological Symptoms/Findings
**Top Five Fit, Top Fit (<= 1 month early)**	Male	33			spots on the lower extremity (color: purple pruritus: no)<br>spots on the trunk (color: purple pruritus: no)<br>spots on the upper extremity (color: purple pruritus: no)<br>swelling, wrist		
**Diagnosis**	Male	33	Henoch-Schonlein Purpura (HSP)	elevated BMI	spots on the lower extremity (color: purple pruritus: no time since onset: one week to one month)<br>spots on the trunk (color: purple pruritus: no time since onset: one week to one month)<br>spots on the upper extremity (color: purple pruritus: no time since onset: one week to one month)<br>swelling, wrist (time since onset: one week to one month)<br>weight gain<br>swelling of the lower extremity<br>foamy urine<br>dyspnea<br>post-nasal drip<br>mesangiocapillary glomerulonephritis<br>proteinuria (intensity: moderate to severe)<br>cholesterol level (result: elevated)<br>triglyceride level (result: elevated)<br>nephrotic syndrome<br>peripheral edema (pitting)<br>dysmorphic RBCs in urine		epistaxis<br>hemoptysis<br>antinuclear antibody level, elevated<br>antineutrophil cytoplasmic antibodies present<br>anti-dsDNA antibodies<br>Hepatitis B virus<br>confirmed human immunodeficiency virus infection<br>IgG level<br>hyperproteinemia
**31**							
**Visit**	Sex	Age	Confirmed Diagnoses	Present Risk Factors	Pathological Symptoms/Findings (with attributes)	Absent Risk Factors	Non-pathological Symptoms/Findings
**Top Five Fit**	Female	47			facial nerve palsy (position: peripheral time since onset: one month to one year)<br>hearing deficit (time since onset: one month to one year laterality: bilateral)<br>dizziness (time since onset: one week to one month)<br>nystagmus (direction: horizontal)<br>mastoiditis<br>vesicle in the oral cavity (time since onset: one month to one year)<br>swelling, ankle joint (laterality: unilateral)		
**Top Fit**	Female	47			facial nerve palsy (position: peripheral time since onset: one month to one year)<br>hearing deficit (time since onset: one month to one year laterality: bilateral)<br>dizziness (time since onset: one week to one month)<br>mastoiditis<br>rapid pulse<br>palpitation<br>weight loss<br>irregular menstrual bleeding<br>vesicle in the oral cavity (time since onset: one month to one year)<br>swelling, ankle joint (time since onset: one month to one year laterality: unilateral)<br>vertigo (time since onset: one month to one year)<br>complement C3 level (result: reduced)<br>complement C4 level (result: reduced)<br>anticardiolipin antibodies, elevated<br>Melkersson-Rosenthal syndrome		anti-SSA autoantibodies<br>anti-La (SS-B)-autoantibodies<br>anti-U1-RNP antibodies<br>anti-Sm antibodies<br>anti-Scl-70<br>anti-Jo 1 antibodies<br>anti-dsDNA antibodies<br>anti-alpha-fodrin antibodies<br>cytoplasmic antineutrophil cytoplasmic antibodies present<br>perinuclear anti-neutrophil cytoplasmic antibodies present<br>antinuclear antibody level, elevated
**Diagnosis**	Female	48	Sarcoidosis		facial nerve palsy (position: peripheral time since onset: one month to one year)<br>hearing deficit (time since onset: one month to one year laterality: bilateral)<br>dizziness (time since onset: one week to one month)<br>mastoiditis<br>rapid pulse<br>palpitation<br>weight loss<br>irregular menstrual bleeding<br>vesicle in the oral cavity (time since onset: one month to one year)<br>swelling, ankle joint (time since onset: one month to one year laterality: unilateral)<br>vertigo (time since onset: one month to one year)<br>complement C3 level (result: reduced)<br>complement C4 level (result: reduced)<br>anticardiolipin antibodies, elevated<br>Melkersson-Rosenthal syndrome		anti-SSA autoantibodies<br>anti-La (SS-B)-autoantibodies<br>anti-U1-RNP antibodies<br>anti-Sm antibodies<br>anti-Scl-70<br>anti-Jo 1 antibodies<br>anti-dsDNA antibodies<br>anti-alpha-fodrin antibodies<br>cytoplasmic antineutrophil cytoplasmic antibodies present<br>perinuclear anti-neutrophil cytoplasmic antibodies present<br>antinuclear antibody level, elevated
**32**							
**Visit**	Sex	Age	Confirmed Diagnoses	Present Risk Factors	Pathological Symptoms/Findings (with attributes)	Absent Risk Factors	Non-pathological Symptoms/Findings
**Diagnosis**	Male	35	Chronic Polyarthritis<br>Familial Mediterranean Fever (FMF)	Recent travel to tropic<br>elevated BMI	elbow pain (time since onset: more than one year laterality: bilateral)<br>knee pain, patellar (time since onset: more than one year)<br>morning stiffness (time since onset: more than one year)<br>acne<br>swelling, knee (laterality: unilateral time since onset: more than one year)<br>foot pain (laterality: bilateral time since onset: more than one year)<br>tenderness, knee (laterality: unilateral time since onset: more than one year)<br>tenderness, wrist (laterality: unilateral time since onset: more than one year)<br>effusion, knee<br>toe pain (laterality: bilateral time since onset: more than one year)<br>creatine kinase level, elevated<br>polyuria<br>polydipsia<br>fatigue<br>headache<br>IgG4 level (result: elevated)<br>tenderness, MCP joints (time since onset: more than one year laterality: bilateral)<br>MEFV gene mutation		Lymphadenopathy on palpation<br>enlarged thyroid gland<br>erythema of the oral cavity<br>effusion, ankle<br>fever<br>double vision<br>disorientation<br>facial pallor<br>elevated rheumatoid factor<br>anti-cyclic citrullinated protein antibodies<br>antinuclear antibody level, elevated<br>anti-Sm antibodies<br>anti-U1-RNP antibodies<br>anti-SSA autoantibodies<br>anti-La (SS-B)-autoantibodies<br>anti-Scl-70<br>anti-Jo 1 antibodies<br>anti-alpha-fodrin antibodies<br>serum complement level<br>anti-dsDNA antibodies
**33**							
**Visit**	Sex	Age	Confirmed Diagnoses	Present Risk Factors	Pathological Symptoms/Findings (with attributes)	Absent Risk Factors	Non-pathological Symptoms/Findings
**Diagnosis**	Female	55	Familial Mediterranean Fever (FMF)	elevated BMI<br>Hx: hypertension<br>diabetes mellitus	fever (time since onset: more than one year high grade: no)<br>myalgia (time since onset: more than one year)<br>arthralgia (time since onset: more than one year)<br>hyperhidrosis, generalized (night sweats: yes time since onset: more than one year)<br>dyspnea (severity: on exertion)<br>CRP, elevated<br>reduced exercise tolerance<br>diverticula<br>splenomegaly<br>steatosis hepatis<br>ovarian cyst seen on imaging		leukocytosis<br>antinuclear antibody level, elevated<br>antineutrophil cytoplasmic antibodies present<br>antimitochondrial antibody<br>anti-smooth-muscle antibodies<br>vasculitis
**34**							
**Visit**	Sex	Age	Confirmed Diagnoses	Present Risk Factors	Pathological Symptoms/Findings (with attributes)	Absent Risk Factors	Non-pathological Symptoms/Findings
**Top Five Fit, Top Fit**	Male	50			swelling, wrist (time since onset: more than one year)<br>arthritis, wrist (time since onset: more than one year)<br>swelling, ankle joint (laterality: bilateral)<br>arthritis, ankle<br>carpal tunnel syndrome		

**Diagnosis**	Male	59	Whipple disease	smoker<br>elevated BMI	swelling, wrist (time since onset: more than one year)<br>swelling, ankle joint (laterality: bilateral time since onset: more than one year)<br>swelling, finger joints (time since onset: more than one year)<br>swelling, toe joint (time since onset: more than one year)<br>swelling, knee (time since onset: more than one year)<br>wrist pain (time since onset: more than one year laterality: bilateral)<br>ankle pain (time since onset: more than one year laterality: bilateral)<br>finger joint pain (laterality: bilateral time since onset: more than one year)<br>toe pain (laterality: bilateral time since onset: more than one year)<br>knee pain (laterality: bilateral time since onset: more than one year)<br>generalized arthralgia (time since onset: more than one year)<br>effusion, wrist<br>tendinitis, hand and wrist<br>CRP, elevated<br>gamma gt level, elevated<br>uric acid level (result: elevated)<br>peripheral edema (pitting)<br>back pain (time since onset: more than one year)<br>hip pain (time since onset: more than one year)<br>paresthesia of the hand (distribution: asymmetrical time since onset: more than one year)<br>feeling of lump in the throat<br>hyperhidrosis, generalized (night sweats: yes time since onset: more than one year)<br>arthritis (time since onset: more than one year)<br>effusion, knee<br>effusion, ankle<br>right ventricular pressure, elevated<br>pulmonary hypertension<br>erythrocyte sedimentation rate (result: elevated)<br>antinuclear antibody level, elevated<br>helicobacter pylori in stomach/duodenum<br>erosions and ulcerations of the stomach<br>mesenteric lymphadenopathy<br>morning stiffness<br>reduced exercise tolerance<br>abdominal pain (position: upper left quadrant)<br>flank pain (side: left)<br>abdominal tenderness<br>loss of appetite<br>weight loss<br>steatosis hepatis<br>elevated skin temperature of the joint<br>myalgia<br>elevated skin temperature of the ankle<br>anti-SRP antibodies<br>fever<br>Tropheryma whipplei		elevated rheumatoid factor<br>antineutrophil cytoplasmic antibodies present<br>anti-cyclic citrullinated protein antibodies<br>HLA-B27 positive<br>alanine transaminase level, elevated<br>aspartate transaminase level, elevated<br>anti-U1-RNP antibodies<br>anti-Sm antibodies<br>anti-SSA autoantibodies<br>anti-La (SS-B)-autoantibodies<br>anti-Scl-70<br>anti-Jo 1 antibodies
**35**							
**Visit**	Sex	Age	Confirmed Diagnoses	Present Risk Factors	Pathological Symptoms/Findings (with attributes)	Absent Risk Factors	Non-pathological Symptoms/Findings
**Top Five Fit, Top Fit (<= 1 month early)**	Male	28		Recent travel to tropic<br>Hx: hypertension	fatigue<br>pruritus, upper extremity<br>pruritus, lower extremity<br>fever (time since onset: one week to one month)<br>cough (clear or yellow phlegm: yes time since onset: one week to one month)<br>sputum (bloody: no time since onset: one week to one month)<br>nasal discharge (time since onset: one week to one month)<br>dyspnea (time since onset: one week to one month)<br>left ventricular dilatation<br>cardiac ejection fraction (result: reduced)<br>pericardial effusion<br>serum cardiac troponin level, elevated<br>dilated cardiomyopathy<br>renal insufficiency<br>glomerular filtration rate, reduced		stenosis of the coronary artery
**Diagnosis**	Male	28	Takayasu's arteritis	Recent travel to tropic<br>Hx: hypertension	fatigue<br>pruritus, upper extremity (time since onset: one month to one year)<br>pruritus, lower extremity (time since onset: one month to one year)<br>fever (time since onset: one month to one year)<br>cough (clear or yellow phlegm: yes time since onset: one month to one year)<br>sputum (bloody: no time since onset: one month to one year)<br>nasal discharge (time since onset: one month to one year)<br>dyspnea (time since onset: one month to one year)<br>left ventricular dilatation<br>cardiac ejection fraction (result: reduced)<br>pericardial effusion<br>serum cardiac troponin level, elevated<br>dilated cardiomyopathy<br>renal insufficiency<br>glomerular filtration rate, reduced<br>myocarditis<br>hypertrophic cardiomyopathy		stenosis of the coronary artery<br>antinuclear antibody level, elevated<br>antineutrophil cytoplasmic antibodies present
**36**							
**Visit**	Sex	Age	Confirmed Diagnoses	Present Risk Factors	Pathological Symptoms/Findings (with attributes)	Absent Risk Factors	Non-pathological Symptoms/Findings
**Top Five Fit**	Female	56			hip pain (laterality: bilateral intensity: severe time since onset: more than one year)<br>gait impairment<br>paresthesia of the lower extremity (distribution: symmetrical time since onset: one month to one year)<br>lower leg pain (laterality: bilateral time since onset: one month to one year)<br>chills (time since onset: one month to one year)<br>hyperhidrosis, generalized (night sweats: yes paroxysmal sweats: yes time since onset: more than one year)<br>chest pain (time since onset: one month to one year)<br>flushing (time since onset: one month to one year)<br>alopecia (time since onset: one month to one year)<br>loose teeth<br>headache (time since onset: one month to one year)<br>palpitation (time since onset: one month to one year)<br>chest tightness (time since onset: one month to one year)<br>high blood pressure (time since onset: one month to one year)<br>agitation<br>swelling of the lower leg (laterality: bilateral time since onset: one month to one year)<br>lower extremity pain (laterality: bilateral time since onset: one month to one year)<br>fever (time since onset: one month to one year)<br>erythema of the trunk<br>erythema of the cheeks<br>erythema of the thigh<br>vaginal wall bleeding<br>Insulin-like growth factor 1 level (result: elevated)<br>lowered skin temperature of the leg<br>lowered skin temperature of the foot<br>increased urinary frequency<br>constipation<br>MCP I joint pain (laterality: bilateral)<br>limited range of motion of the MCP I joint<br>dyspnea (severity: on exertion)<br>painful dysesthesia of the lower extremity (distribution: symmetrical)<br>groin pain<br>vertigo<br>facial pain<br>sinus pain<br>swelling of the nasal and sinus mucosa<br>gastroesophageal reflux<br>swelling of the eyelid<br>polydipsia<br>swelling, ankle joint<br>abdominal pain (position: epigastric)<br>feeling of lump in the throat<br>temporomandibular joint pain<br>crepitus, temporomandibular joint<br>disc displacement of the temporomandibular joint<br>bone degeneration of the temporomandibular joint<br>fatigue<br>finger joint pain (laterality: bilateral)<br>tenderness, finger joint (laterality: bilateral)<br>tenderness, hip (laterality: bilateral)<br>sensory deficit, lower extremity (distribution: bilateral, symmetrical)<br>antinuclear antibody level, elevated<br>anti-PmScl antibody titer<br>anti-PL 12 antibodies<br>elevated rheumatoid factor		diminished lower extremity pulse<br>deep vein thrombosis in the lower extremity<br>pulmonary embolism<br>intracerebral mass<br>aneurysm of the intracranial arteries<br>increased intracranial pressure<br>air-fluid levels in the sinus<br>serum cardiac troponin level, elevated<br>sinusitis<br>thyroid stimulating hormone level<br>enlarged thyroid gland<br>hearing deficit<br>anti-dsDNA antibodies<br>anti-Sm antibodies<br>anti-U1-RNP antibodies<br>anti-La (SS-B)-autoantibodies<br>anti-SSA autoantibodies<br>anti-Scl-70<br>anti-Jo 1 antibodies<br>MI-2 antibody titer<br>anti-EJ antibodies<br>anticardiolipin antibodies, elevated<br>anti-alpha-fodrin antibodies<br>complement C3 level<br>complement C4 level

**Diagnosis**	Female	57	Polymyositis/scleroderma overlap		hip pain (laterality: bilateral intensity: severe time since onset: more than one year)<br>gait impairment<br>paresthesia of the lower extremity (distribution: symmetrical time since onset: one month to one year)<br>lower leg pain (laterality: bilateral time since onset: one month to one year)<br>chills (time since onset: one month to one year)<br>hyperhidrosis, generalized (night sweats: yes paroxysmal sweats: yes time since onset: more than one year)<br>flushing (time since onset: one month to one year)<br>alopecia (time since onset: one month to one year)<br>loose teeth<br>headache (time since onset: one month to one year)<br>palpitation (time since onset: one month to one year)<br>high blood pressure (time since onset: one month to one year)<br>agitation<br>lower extremity pain (laterality: bilateral time since onset: one month to one year)<br>erythema of the trunk<br>erythema of the cheeks<br>erythema of the thigh<br>vaginal wall bleeding<br>Insulin-like growth factor 1 level (result: elevated)<br>lowered skin temperature of the leg<br>lowered skin temperature of the foot<br>MCP I joint pain (laterality: bilateral)<br>limited range of motion of the MCP I joint<br>painful dysesthesia of the lower extremity (distribution: symmetrical)<br>groin pain<br>vertigo<br>facial pain<br>sinus pain<br>swelling of the nasal and sinus mucosa<br>gastroesophageal reflux<br>polydipsia<br>feeling of lump in the throat<br>temporomandibular joint pain<br>crepitus, temporomandibular joint<br>disc displacement of the temporomandibular joint<br>bone degeneration of the temporomandibular joint<br>finger joint pain (laterality: bilateral)<br>tenderness, finger joint (laterality: bilateral)<br>tenderness, hip (laterality: bilateral)<br>lower back pain<br>lowered skin temperature of the hand<br>morning stiffness<br>swelling, PIP joints (finger)<br>anti-PmScl antibody titer<br>antinuclear antibody level, elevated<br>periorbital pain<br>anti-PL 12 antibodies<br>proteinuria<br>glomerular filtration rate, reduced<br>flank pain<br>leukocyturia<br>brain white matter lesion<br>pruritus, trunk<br>pruritus, palms<br>erythema nodosum		fever<br>diminished lower extremity pulse<br>deep vein thrombosis in the lower extremity<br>pulmonary embolism<br>intracerebral mass<br>aneurysm of the intracranial arteries<br>increased intracranial pressure<br>air-fluid levels in the sinus<br>serum cardiac troponin level, elevated<br>sinusitis<br>thyroid stimulating hormone level<br>enlarged thyroid gland<br>hearing deficit<br>leukocyte glucocerebrosidase activity<br>limited range of motion of hip joint<br>EMG abnormalities<br>slowed nerve conduction velocity<br>creatine kinase level, elevated<br>anti-dsDNA antibodies<br>elevated rheumatoid factor<br>anti-Sm antibodies<br>anti-SSA autoantibodies<br>anti-La (SS-B)-autoantibodies<br>anti-U1-RNP antibodies<br>anti-Scl-70<br>anti-Jo 1 antibodies<br>MI-2 antibody titer<br>anti-EJ antibodies<br>anticardiolipin antibodies, elevated<br>anti-alpha-fodrin antibodies<br>complement C3 level<br>complement C4 level
**37**							
**Visit**	Sex	Age	Confirmed Diagnoses	Present Risk Factors	Pathological Symptoms/Findings (with attributes)	Absent Risk Factors	Non-pathological Symptoms/Findings
**Top Five Fit (<= 1 month early)**	Female	28		Recent travel to tropic	cheek pain (time since onset: one month to one year)<br>otalgia (time since onset: one month to one year)<br>facial pain (time since onset: one month to one year)<br>anterior neck pain (position: lateral time since onset: one month to one year)<br>dizziness (time since onset: one month to one year)<br>headache (laterality: unilateral)<br>weight loss<br>erythrocyte sedimentation rate (result: elevated)<br>stenosis of the carotid artery		
**Diagnosis**	Female	28	Takayasu's arteritis	Recent travel to tropic	cheek pain (time since onset: one month to one year)<br>otalgia (time since onset: one month to one year)<br>facial pain (time since onset: one month to one year)<br>anterior neck pain (position: lateral time since onset: one month to one year)<br>dizziness (time since onset: one month to one year)<br>headache (laterality: unilateral time since onset: one month to one year)<br>erythrocyte sedimentation rate (result: elevated)<br>stenosis of the carotid artery<br>vasculitis<br>reduced exercise tolerance<br>epistaxis		weight loss<br>hyperhidrosis, generalized<br>chest pain<br>syncope<br>dyspnea<br>tricolor pattern of discoloration of the fingers and toes<br>impaired vision<br>arthralgia<br>skin rash<br>paresthesia<br>muscle cramps<br>dysuria<br>stenosis of the renal artery
**38**							
**Visit**	Sex	Age	Confirmed Diagnoses	Present Risk Factors	Pathological Symptoms/Findings (with attributes)	Absent Risk Factors	Non-pathological Symptoms/Findings
**Diagnosis**	Male	45	Thrombangiitis obliterans<br>Panarteritis nodosa		thrombosis, portal vein<br>deep vein thrombosis in the lower extremity<br>generalized lymphadenopathy<br>splenic artery thrombosis<br>malaise<br>fever<br>foot pain<br>lower back pain<br>cachexia<br>peripheral edema (pitting) (laterality: bilateral)<br>oropharyngeal candidiasis<br>submandibular lymphadenopathy (consistency: soft mobility: mobile tenderness: non-tender)<br>erythema of the palm<br>caput medusae<br>hepatomegaly<br>necrosis of the toes<br>blue skin, foot<br>vasculitis		antiphospholipid antibodies, elevated<br>plasma cell infiltration<br>cough<br>sputum<br>disorientation<br>partial loss of consciousness<br>distended jugular veins<br>scleral icterus<br>change in pupil diameter<br>anisocoria<br>conjunctival injection<br>oculomotor nerve palsy<br>dry mouth<br>cranial nerve disorder<br>enlarged thyroid gland<br>axillary lymphadenopathy<br>inguinal lymphadenopathy<br>facial pallor<br>jaundice<br>cyanosis<br>prolonged capillary refill<br>spider angiomata<br>nail clubbing<br>abdominal mass on examination<br>pallor of upper extremity<br>pallor of lower extremity<br>increased range of joint motion<br>purpura<br>petechiae
**39**							
**Visit**	Sex	Age	Confirmed Diagnoses	Present Risk Factors	Pathological Symptoms/Findings (with attributes)	Absent Risk Factors	Non-pathological Symptoms/Findings
**Top Five Fit**	Male	12			abdominal pain (time since onset: more than one year)<br>fever (time since onset: more than one year high grade: yes)<br>diarrhea (time since onset: more than one year)<br>swelling, knee (time since onset: more than one year laterality: unilateral)<br>hip pain (time since onset: more than one year laterality: unilateral)<br>conjunctivitis (time since onset: more than one year)<br>periorbital swelling (time since onset: more than one year)<br>fatigue<br>CRP, elevated<br>erythrocyte sedimentation rate (result: elevated)<br>leukocytosis<br>erythema of the skin, localized (time since onset: more than one year)		
**Top Fit**	Male	13			abdominal pain (time since onset: more than one year)<br>fever (time since onset: more than one year high grade: yes)<br>diarrhea (time since onset: more than one year)<br>swelling, knee (time since onset: more than one year laterality: unilateral)<br>hip pain (time since onset: more than one year laterality: unilateral)<br>conjunctivitis (time since onset: more than one year)<br>periorbital swelling (time since onset: more than one year)<br>fatigue<br>CRP, elevated<br>erythrocyte sedimentation rate (result: elevated)<br>leukocytosis<br>erythema of the skin, localized (time since onset: more than one year)<br>hyperhidrosis, generalized (night sweats: yes time since onset: one month to one year)		
**Diagnosis**	Male	40	TNF receptor associated periodic syndrome (TRAPS)		TNFRSF1A gene mutation<br>serum creatinine level (result: elevated)<br>proteinuria (intensity: moderate to severe)<br>amyloid protein deposition<br>splenomegaly<br>mesenteric lymphadenopathy<br>weight loss (more than 10% of body weight within six months: no)<br>pericarditis<br>epididymitis<br>abdominal pain (time since onset: more than one year)<br>fever (time since onset: more than one year high grade: yes)<br>diarrhea (time since onset: more than one year)<br>swelling, knee (time since onset: more than one year laterality: unilateral)<br>hip pain (time since onset: more than one year laterality: unilateral)<br>conjunctivitis (time since onset: more than one year)<br>periorbital swelling (time since onset: more than one year)<br>fatigue<br>CRP, elevated<br>erythrocyte sedimentation rate (result: elevated)<br>leukocytosis<br>hypoalbuminemia<br>erythema of the skin, localized (time since onset: more than one year)<br>hyperhidrosis, generalized (night sweats: yes time since onset: one month to one year)		antinuclear antibody level, elevated<br>antimitochondrial antibody<br>cytoplasmic antineutrophil cytoplasmic antibodies present<br>anti-smooth-muscle antibodies<br>anti-Saccharomyces cerevisiae antibodies<br>gliadin antibodies
**40**							
**Visit**	Sex	Age	Confirmed Diagnoses	Present Risk Factors	Pathological Symptoms/Findings (with attributes)	Absent Risk Factors	Non-pathological Symptoms/Findings

**Diagnosis**	Female	36	Familial Mediterranean Fever (FMF)<br>Behcet's disease		fever (time since onset: more than one year high grade: no)<br>malaise<br>reduced exercise tolerance (time since onset: more than one year)<br>AV block second degree<br>lower back pain (Intensity: severe time since onset: more than one year)<br>double vision (time since onset: more than one year)<br>arthritis, shoulder (time since onset: more than one year)<br>myalgia (time since onset: more than one year)<br>proteinuria (intensity: trace to mild)<br>headache (time since onset: one month to one year)<br>perioral paresthesia (time since onset: one month to one year)<br>sensory deficit, face (distribution: unilateral time since onset: one month to one year)<br>finger pain (laterality: unilateral time since onset: one month to one year)<br>CRP, elevated<br>leukocytosis<br>erythrocyte sedimentation rate (result: elevated)<br>procalcitonin level, elevated<br>alpha 2 globulin level (result: elevated)<br>temporary loss of consciousness<br>aphthous ulcerations of the oral cavity (time since onset: more than one year)<br>mesangial cell proliferation<br>microhematuria<br>inflammatory back pain<br>MEFV gene mutation<br>aphthous ulcerations of the genital area		eosinophilia in blood<br>antinuclear antibody level, elevated<br>cytoplasmic antineutrophil cytoplasmic antibodies present<br>perinuclear anti-neutrophil cytoplasmic antibodies present<br>anticardiolipin antibodies, elevated<br>anti-dsDNA antibodies<br>thyroid peroxidase antibodies<br>thyrotropin receptor antibodies<br>thyroglobulin antibodies
**41**							
**Visit**	Sex	Age	Confirmed Diagnoses	Present Risk Factors	Pathological Symptoms/Findings (with attributes)	Absent Risk Factors	Non-pathological Symptoms/Findings
**Top Five Fit, Top Fit**	Male	8			fever (high grade: yes time since onset: more than one year)<br>urticaria (time since onset: more than one year)<br>arthritis, knee (time since onset: more than one year)		
**Diagnosis**	Male	37	TNF receptor associated periodic syndrome (TRAPS)<br>Cryopyrin-associated periodic syndrome (CAPS)		fever (high grade: yes time since onset: more than one year)<br>urticaria (time since onset: more than one year)<br>arthritis, knee (time since onset: more than one year)<br>sensorineural hearing deficit (laterality: bilateral)<br>panic attacks<br>supraventricular arrhythmias in ECG<br>CRP, elevated<br>arthritis (time since onset: more than one year)<br>TNFRSF1A gene mutation<br>NLRP3 gene mutation		
**42**							
**Visit**	Sex	Age	Confirmed Diagnoses	Present Risk Factors	Pathological Symptoms/Findings (with attributes)	Absent Risk Factors	Non-pathological Symptoms/Findings
**Top Five Fit, Top Fit**	Female	15			lower back pain (time since onset: one month to one year Intensity: severe)<br>sinusitis<br>CRP, elevated<br>papule on the scalp<br>papule on the upper extremity<br>papule on the lower extremity<br>reduced exercise tolerance (time since onset: one month to one year)<br>osteomyelitis<br>antinuclear antibody level, elevated<br>erythrocyte sedimentation rate (result: elevated)<br>IgG level (result: elevated)<br>platelet count (result: elevated)<br>anemia<br>aspartate transaminase level, elevated<br>alanine transaminase level, elevated<br>gamma gt level, elevated<br>fever (time since onset: one month to one year high grade: no)<br>dry skin on other location<br>leukocytosis		dyspnea<br>tachypnea<br>anti-dsDNA antibodies<br>anti-Sm antibodies<br>anti-U1-RNP antibodies<br>anti-La (SS-B)-autoantibodies<br>anti-SSA autoantibodies<br>anti-Scl-70<br>anti-Jo 1 antibodies<br>lactate dehydrogenase level, elevated<br>alkaline phosphatase level<br>serum creatinine level<br>proteinuria<br>pitted nails<br>oil drop nail<br>endocarditis<br>swelling, joint<br>abdominal tenderness<br>hepatomegaly<br>splenomegaly<br>abdominal distension<br>abdominal pain<br>abdominal mass on examination
**Diagnosis**	Female	15	Takayasu's arteritis		lower back pain (time since onset: one month to one year Intensity: severe)<br>sinusitis<br>CRP, elevated<br>papule on the scalp<br>papule on the upper extremity<br>papule on the lower extremity<br>reduced exercise tolerance (time since onset: one month to one year)<br>dyspnea<br>osteomyelitis<br>antinuclear antibody level, elevated<br>erythrocyte sedimentation rate (result: elevated)<br>IgG level (result: elevated)<br>anemia<br>gamma gt level, elevated<br>serum creatinine level (result: elevated)<br>fever (time since onset: one month to one year high grade: no)<br>dry skin on other location<br>abdominal pain (position: epigastric eating: no effect)<br>flank pain (side: bilateral)<br>vomiting (time since onset: one week to one month)<br>loss of appetite<br>high blood pressure<br>facial pallor<br>serum CA-125 level, elevated<br>blood urea nitrogen level (result: elevated)<br>convulsion<br>fecal calprotectin, elevated<br>stenosis of the renal artery<br>stenosis of the mesenteric artery		tachypnea<br>anti-dsDNA antibodies<br>anti-Sm antibodies<br>anti-U1-RNP antibodies<br>anti-La (SS-B)-autoantibodies<br>anti-SSA autoantibodies<br>anti-Scl-70<br>anti-Jo 1 antibodies<br>platelet count<br>aspartate transaminase level, elevated<br>alanine transaminase level, elevated<br>lactate dehydrogenase level, elevated<br>alkaline phosphatase level<br>proteinuria<br>pitted nails<br>oil drop nail<br>endocarditis<br>swelling, joint<br>abdominal tenderness<br>hepatomegaly<br>splenomegaly<br>abdominal distension<br>abdominal mass on examination<br>leukocyte level<br>cervical lymphadenopathy<br>glomerulonephritis<br>gliadin antibodies
**43**							
**Visit**	Sex	Age	Confirmed Diagnoses	Present Risk Factors	Pathological Symptoms/Findings (with attributes)	Absent Risk Factors	Non-pathological Symptoms/Findings
**Top Five Fit**	Female	56			cough (bloody: yes time since onset: one month to one year)<br>weight loss<br>pulmonary nodules<br>cytoplasmic antineutrophil cytoplasmic antibodies present<br>epistaxis<br>CRP, elevated		glomerulonephritis<br>glomerular filtration rate, reduced
**Diagnosis**	Female	59	Sarcoidosis		pulmonary nodules<br>epistaxis (time since onset: more than one year)<br>hyperhidrosis, generalized (time since onset: one month to one year)<br>proteinase 3 antibody titer, elevated<br>fatigue (time since onset: more than one year)<br>hemoptysis (time since onset: one month to one year)<br>reduced exercise tolerance (time since onset: one month to one year)<br>dyspnea (severity: on exertion time since onset: one month to one year)<br>pulmonary radiopacity<br>adrenal mass seen on imaging<br>nasal discharge (appearance: crusty)<br>urinary tract infection<br>thyroid stimulating hormone level (result: reduced)<br>knee pain<br>elbow pain<br>cervical lymphadenopathy<br>sore throat<br>gingivitis<br>facial swelling<br>peripheral edema (pitting)<br>submandibular lymphadenopathy<br>skin abscess<br>facial nerve palsy<br>lagophthalmos<br>microcytic anemia<br>hyperkalemia<br>eosinophils in bronchoalveolar lavage<br>Melkersson-Rosenthal syndrome		cough<br>CRP, elevated<br>glomerulonephritis<br>glomerular filtration rate, reduced<br>arthralgia<br>procalcitonin level, elevated<br>proteinuria<br>fever<br>dysuria
**44**							
**Visit**	Sex	Age	Confirmed Diagnoses	Present Risk Factors	Pathological Symptoms/Findings (with attributes)	Absent Risk Factors	Non-pathological Symptoms/Findings
**Top Five Fit**	Female	16			leukopenia<br>neutropenia<br>splenomegaly<br>submandibular lymphadenopathy<br>microcytic anemia<br>erythrocyte sedimentation rate (result: elevated)		
**Top Fit**	Female	23			leukopenia<br>neutropenia<br>splenomegaly<br>submandibular lymphadenopathy<br>microcytic anemia<br>erythrocyte sedimentation rate (result: elevated)<br>lower back pain (time since onset: more than one year radiating down the leg: yes)<br>reduced exercise tolerance (time since onset: more than one year)<br>imbalance (time since onset: more than one year)<br>renal cysts<br>disc herniation<br>arthritis, PIP joints (finger)<br>wrist pain (laterality: bilateral)<br>knee pain (laterality: unilateral)<br>ankle pain (laterality: bilateral)<br>hip pain (laterality: unilateral)<br>limited range of motion of the finger joints		swelling, joint

**Diagnosis**	Female	27	Felty syndrome		leukopenia<br>neutropenia<br>splenomegaly<br>submandibular lymphadenopathy<br>microcytic anemia<br>erythrocyte sedimentation rate (result: elevated)<br>lower back pain (time since onset: more than one year radiating down the leg: yes)<br>reduced exercise tolerance (time since onset: more than one year)<br>imbalance (time since onset: more than one year)<br>renal cysts<br>disc herniation<br>arthritis, PIP joints (finger) (time since onset: more than one year)<br>wrist pain (laterality: bilateral time since onset: more than one year)<br>knee pain (laterality: unilateral time since onset: one month to one year)<br>ankle pain (laterality: bilateral time since onset: one month to one year)<br>hip pain (laterality: unilateral time since onset: more than one year)<br>limited range of motion of the finger joints (time since onset: more than one year)<br>proof of miscarriage<br>thrombophlebitis<br>neck pain (time since onset: more than one year)<br>gait impairment (time since onset: more than one year)<br>dizziness (time since onset: more than one year)<br>headache (time since onset: one month to one year)<br>dyspnea (severity: on exertion time since onset: one month to one year)<br>calf pain (laterality: bilateral time since onset: one month to one year)<br>muscular atrophy of the lower limb (time since onset: one month to one year)<br>paresthesia of the hand (time since onset: one month to one year)<br>paresthesia of the lower extremity (time since onset: one month to one year)<br>foot pain (laterality: bilateral time since onset: one month to one year)<br>painful dysesthesia of the lower extremity (time since onset: one month to one year)<br>painful dysesthesia of the upper extremity (time since onset: one month to one year)<br>hepatomegaly<br>nephrocalcinosis<br>hypophosphatemia<br>granulocytopenia		swelling, joint<br>peripheral vascular disease<br>decreased venous flow in the lower extremity<br>hyperreflexia, upper limb<br>hyperreflexia, lower limb<br>diminished reflexes, upper limb<br>diminished reflexes, lower limb<br>pulmonary embolism<br>myositis<br>peripheral polyneuropathy<br>pulmonary infiltrates<br>pulmonary mass
**45**							
**Visit**	Sex	Age	Confirmed Diagnoses	Present Risk Factors	Pathological Symptoms/Findings (with attributes)	Absent Risk Factors	Non-pathological Symptoms/Findings
**Top Five Fit**	Female	32			neck pain (time since onset: one month to one year)<br>leukocytosis<br>thrombocytosis<br>swelling, wrist (time since onset: one month to one year)<br>wrist pain (time since onset: one month to one year)<br>increased range of joint motion<br>shoulder pain (laterality: unilateral)<br>elbow pain (laterality: unilateral)<br>knee pain (laterality: unilateral)<br>heel pain (laterality: bilateral)<br>lower back pain<br>gluteal pain		HLA-B27 positive
**Diagnosis**	Female	32	Ankylosing spondylitis (Spondyloarthritis)		neck pain (time since onset: one month to one year)<br>leukocytosis<br>thrombocytosis<br>swelling, wrist (time since onset: one month to one year)<br>wrist pain (time since onset: one month to one year)<br>increased range of joint motion<br>shoulder pain (laterality: unilateral)<br>elbow pain (laterality: unilateral)<br>knee pain (laterality: unilateral)<br>heel pain (laterality: bilateral)<br>lower back pain<br>gluteal pain<br>HLA-B51 positive		HLA-B27 positive
**46**							
**Visit**	Sex	Age	Confirmed Diagnoses	Present Risk Factors	Pathological Symptoms/Findings (with attributes)	Absent Risk Factors	Non-pathological Symptoms/Findings
**Diagnosis**	Female	54	Psoriatic arthritis (Spondyloarthritis)		antinuclear antibody level, elevated<br>creatine kinase level, elevated<br>weight gain (rapid: no)<br>erythema of the trunk (scaly surface: yes time since onset: more than one year)<br>cholesterol level (result: elevated)<br>thyroid stimulating hormone level (result: elevated)<br>scaling skin of the extensor surfaces of the extremities<br>arthralgia (time since onset: more than one year)<br>morning stiffness (time since onset: one month to one year)<br>enthesitis<br>fatigue<br>shoulder pain (laterality: unilateral)<br>exocrine dysfunction of the pancreas<br>tenderness, MCP joints (laterality: bilateral)<br>tenderness, MTP joints (laterality: bilateral)<br>general muscle weakness (time since onset: more than one year)<br>myalgia (time since onset: more than one year)<br>skin rash of the extensor surfaces of the extremities (elevated: yes time since onset: more than one year)<br>heel pain<br>dry eye<br>effusion, knee<br>tenderness, knee<br>tenderness, hip		CRP, elevated
**47**							
**Visit**	Sex	Age	Confirmed Diagnoses	Present Risk Factors	Pathological Symptoms/Findings (with attributes)	Absent Risk Factors	Non-pathological Symptoms/Findings
**Diagnosis**	Male	48	Thrombotic thrombocytopenic purpura (TTP)		vertigo<br>imbalance<br>thrombocytopenia<br>alanine transaminase level, elevated<br>impaired concentration<br>reduced exercise tolerance (time since onset: one day to one week)<br>lactate dehydrogenase level, elevated<br>serum creatinine level (result: elevated)<br>glomerular filtration rate, reduced<br>blood urea nitrogen level (result: elevated)<br>aspartate transaminase level, elevated<br>lipase level, elevated<br>procalcitonin level, elevated<br>hemoglobin level (result: reduced)<br>d-dimer level in blood, elevated<br>ADAMTS-13 activity, decreased<br>trigeminal nerve disorder<br>schistocytes<br>partial loss of consciousness		
**48**							
**Visit**	Sex	Age	Confirmed Diagnoses	Present Risk Factors	Pathological Symptoms/Findings (with attributes)	Absent Risk Factors	Non-pathological Symptoms/Findings
**Diagnosis**	Female	66	Cryoglobulinemia		extremity pain (time since onset: one month to one year)<br>painful dysesthesia of the hand (distribution: asymmetrical time since onset: one month to one year)<br>neck pain (time since onset: one month to one year)<br>limited range of motion of the wrist (time since onset: one month to one year)<br>weight loss<br>complement C3 level (result: elevated)<br>CRP, elevated<br>erythrocyte sedimentation rate (result: elevated)<br>alpha 2 globulin level (result: elevated)<br>ferritin level (result: elevated)<br>alanine transaminase level, elevated<br>stenosis of the subclavian artery<br>pathologically increased cryoglobulin level<br>erosion and ulceration of the lower extremity<br>elevated rheumatoid factor		proteinase 3 antibody titer, elevated<br>myeloperoxidase antibodies present<br>complement C4 level<br>antinuclear antibody level, elevated
**49**							
**Visit**	Sex	Age	Confirmed Diagnoses	Present Risk Factors	Pathological Symptoms/Findings (with attributes)	Absent Risk Factors	Non-pathological Symptoms/Findings
**Top Five Fit, Top Fit**	Male	38			proof of acute pancreatitis<br>hypothyroidism<br>antinuclear antibody level, elevated		anti-dsDNA antibodies
**Diagnosis**	Male	47	IgG4-related disease	smoker	proof of acute pancreatitis<br>hypothyroidism<br>antinuclear antibody level, elevated<br>obstructive sleep apnea<br>thyroid stimulating hormone level (result: elevated)<br>thyroid peroxidase antibodies<br>free T4 (result: reduced)<br>leukocytosis<br>lipase level, elevated<br>CRP, elevated<br>gallstone<br>gamma gt level, elevated<br>pruritus<br>speech difficulties<br>stenosis of the carotid artery<br>vasculitis<br>dilated cardiomyopathy<br>bradycardia on ECG<br>atrioventricular dissociation<br>erythrocyte sedimentation rate (result: elevated)<br>perinuclear anti-neutrophil cytoplasmic antibodies present<br>parathyroid hormone level (result: elevated)<br>macrocytic anemia<br>depressed mood<br>nausea<br>weight loss<br>IgG4 level (result: elevated)		pancreatic pseudocyst/ cyst<br>transaminase levels, elevated<br>cardiac ejection fraction<br>calcium level<br>anti-dsDNA antibodies
**50**							
**Visit**	Sex	Age	Confirmed Diagnoses	Present Risk Factors	Pathological Symptoms/Findings (with attributes)	Absent Risk Factors	Non-pathological Symptoms/Findings
**Diagnosis**	Male	70	IgG4-related disease<br>Retroperitoneal fibrosis		dilated renal calyces<br>ureteric stenosis<br>serum creatinine level (result: elevated)<br>CRP, elevated<br>periaortic fibrotic mass<br>splenic mass seen on imaging<br>hydroureter<br>retroperitoneal fibrosis<br>predominance of IgG4 positive plasma cells in histopathology<br>plasma cell infiltration		flank pain<br>renal mass seen on imaging<br>Lymphadenopathy on investigation
**51**							

Visit	Sex	Age	Confirmed Diagnoses	Present Risk Factors	Pathological Symptoms/Findings (with attributes)	Absent Risk Factors	Non-pathological Symptoms/Findings
**Top Five Fit, Top Fit**	Female	23		pregnancy, confirmed	chest pain (position: bilateral sharp: yes)<br>pleuritis<br>pleural effusion<br>fever<br>splenomegaly<br>CRP, elevated<br>d-dimer level in blood, elevated<br>loss of appetite<br>nausea<br>malaise<br>sinus tachycardia<br>microcytic anemia		serum creatinine level<br>antinuclear antibody level, elevated<br>antineutrophil cytoplasmic antibodies present<br>HLA-B27 positive<br>anticardiolipin antibodies, elevated<br>anti-beta 2 glycoprotein antibodies, elevated<br>anti-SSA autoantibodies<br>anti-La (SS-B)-autoantibodies<br>anti-Sm antibodies<br>pleural empyema
**Diagnosis**	Female	25	Familial Mediterranean Fever (FMF)		chest pain (position: bilateral sharp: yes time since onset: one month to one year)<br>pleuritis<br>pleural effusion<br>fever (time since onset: one month to one year)<br>splenomegaly<br>CRP, elevated<br>d-dimer level in blood, elevated<br>loss of appetite (time since onset: one month to one year)<br>malaise (time since onset: one month to one year)<br>sinus tachycardia<br>microcytic anemia<br>hypothyroidism<br>lactate dehydrogenase level, elevated<br>lymphopenia<br>sodium level (result: reduced)<br>calcium level (result: reduced)<br>cervical inflammation<br>dyspnea<br>chills<br>MEFV gene mutation		serum creatinine level<br>antinuclear antibody level, elevated<br>antineutrophil cytoplasmic antibodies present<br>HLA-B27 positive<br>anticardiolipin antibodies, elevated<br>anti-beta 2 glycoprotein antibodies, elevated<br>anti-SSA autoantibodies<br>anti-La (SS-B)-autoantibodies<br>anti-Sm antibodies<br>right ventricular pressure, elevated<br>right ventricular enlargement<br>anti-dsDNA antibodies<br>pleural empyema
**52**							
**Visit**	Sex	Age	Confirmed Diagnoses	Present Risk Factors	Pathological Symptoms/Findings (with attributes)	Absent Risk Factors	Non-pathological Symptoms/Findings
**Diagnosis**	Male	55	Systemic sclerosis (renal crisis)		fatigue<br>reduced exercise tolerance (time since onset: one month to one year)<br>malaise (time since onset: one month to one year)<br>tricolor pattern of discoloration of the fingers and toes (time since onset: one month to one year)<br>weight loss<br>arthralgia (time since onset: one month to one year)<br>serum creatinine level (result: elevated)<br>anti-Scl-70<br>restless legs		
**53**							
**Visit**	Sex	Age	Confirmed Diagnoses	Present Risk Factors	Pathological Symptoms/Findings (with attributes)	Absent Risk Factors	Non-pathological Symptoms/Findings
**Top Five Fit**	Female	37			swelling, MCP joints (time since onset: one month to one year)<br>swelling of the fingers (time since onset: one month to one year)<br>myalgia (time since onset: one month to one year)<br>lower extremity pain (time since onset: one month to one year)<br>tricolor pattern of discoloration of the fingers and toes (time since onset: one month to one year)<br>erythema of the forearm<br>CRP, elevated<br>erythrocyte sedimentation rate (result: elevated)<br>leukocytosis<br>anemia<br>neutrophilia<br>lymphopenia<br>creatine kinase level, elevated<br>cardiac CK-MB level, elevated<br>hypoalbuminemia<br>alpha 2 globulin level (result: elevated)<br>antinuclear antibody level, elevated<br>antimitochondrial antibody		anti-dsDNA antibodies
**Top Fit**	Female	42			swelling, MCP joints (time since onset: one month to one year)<br>swelling of the fingers (time since onset: one month to one year)<br>myalgia (time since onset: one month to one year)<br>lower extremity pain (time since onset: one month to one year)<br>tricolor pattern of discoloration of the fingers and toes (time since onset: one month to one year)<br>erythema of the forearm<br>CRP, elevated<br>erythrocyte sedimentation rate (result: elevated)<br>leukocytosis<br>anemia<br>neutrophilia<br>lymphopenia<br>creatine kinase level, elevated<br>cardiac CK-MB level, elevated<br>hypoalbuminemia<br>alpha 2 globulin level (result: elevated)<br>antinuclear antibody level, elevated<br>antimitochondrial antibody<br>myositis<br>myopathic pattern on EMG<br>anti-alpha-fodrin antibodies<br>cough<br>fever<br>pruritus<br>weight loss<br>morning stiffness<br>swelling, knee<br>swelling, ankle joint<br>reduced exercise tolerance<br>cardiac troponin T level, elevated<br>tachycardia on ECG		anti-dsDNA antibodies
**Diagnosis**	Female	44	Antisynthetase Syndrome		swelling, MCP joints (time since onset: one month to one year)<br>swelling of the fingers (time since onset: one month to one year)<br>myalgia (time since onset: one month to one year)<br>lower extremity pain (time since onset: one month to one year)<br>tricolor pattern of discoloration of the fingers and toes (time since onset: one month to one year)<br>erythema of the forearm<br>CRP, elevated<br>erythrocyte sedimentation rate (result: elevated)<br>leukocytosis<br>anemia<br>neutrophilia<br>lymphopenia<br>creatine kinase level, elevated<br>cardiac CK-MB level, elevated<br>hypoalbuminemia<br>alpha 2 globulin level (result: elevated)<br>antinuclear antibody level, elevated<br>antimitochondrial antibody<br>myositis<br>myopathic pattern on EMG<br>anti-alpha-fodrin antibodies<br>cough<br>fever<br>pruritus<br>weight loss<br>morning stiffness<br>swelling, knee<br>swelling, ankle joint<br>reduced exercise tolerance<br>cardiac troponin T level, elevated<br>tachycardia on ECG<br>anti-PL 7 antibodies		anti-dsDNA antibodies<br>anti-Jo 1 antibodies
**54**							
**Visit**	Sex	Age	Confirmed Diagnoses	Present Risk Factors	Pathological Symptoms/Findings (with attributes)	Absent Risk Factors	Non-pathological Symptoms/Findings
**Top Five Fit**	Male	46			anterior ischemic optic neuropathy<br>reduced visual acuity (time since onset: one week to one month)<br>leukocytosis in CSF<br>gamma gt level, elevated<br>LDL level (result: elevated)<br>alanine transaminase level, elevated<br>brain white matter lesion		cardiomegaly<br>pericardial effusion<br>heart valve defect<br>aneurysm of the intracranial arteries
**Diagnosis**	Male	46	Antiphospholipid Syndrome (APS)		anterior ischemic optic neuropathy<br>reduced visual acuity (time since onset: one week to one month)<br>leukocytosis in CSF<br>gamma gt level, elevated<br>LDL level (result: elevated)<br>alanine transaminase level, elevated<br>brain white matter lesion<br>cholesterol level (result: elevated)<br>feeling of heavy legs<br>neck pain<br>lupus anticoagulant antibodies, elevated		cardiomegaly<br>pericardial effusion<br>heart valve defect<br>aneurysm of the intracranial arteries
**55**							
**Visit**	Sex	Age	Confirmed Diagnoses	Present Risk Factors	Pathological Symptoms/Findings (with attributes)	Absent Risk Factors	Non-pathological Symptoms/Findings
**Top Five Fit**	Female	26			microhematuria<br>proteinuria (intensity: trace to mild)<br>parotid gland enlargement<br>fever (high grade: no)<br>erythrocyte sedimentation rate (result: elevated)<br>CRP, elevated<br>transaminase levels, elevated<br>dry mouth<br>reduced exercise tolerance<br>loss of appetite<br>hyperhidrosis, generalized (night sweats: yes)<br>erythema<br>cervical lymphadenopathy<br>axillary lymphadenopathy<br>hyposalivation<br>thinning of the glomerular basement membrane		arthralgia<br>morning stiffness<br>inflammatory back pain<br>aphthous ulcerations of the oral cavity<br>tricolor pattern of discoloration of the fingers and toes<br>venous thrombosis<br>proof of miscarriage<br>dry eye<br>hepatomegaly<br>splenomegaly

**Diagnosis**	Female	28	Sarcoidosis		microhematuria<br>proteinuria (intensity: trace to mild)<br>parotid gland enlargement<br>fever (high grade: no)<br>erythrocyte sedimentation rate (result: elevated)<br>CRP, elevated<br>transaminase levels, elevated<br>dry mouth<br>reduced exercise tolerance<br>loss of appetite<br>hyperhidrosis, generalized (night sweats: yes)<br>erythema<br>axillary lymphadenopathy<br>cervical lymphadenopathy<br>weight loss<br>impaired vision<br>back pain<br>eye pain (intensity: severe laterality: unilateral)<br>anterior uveitis<br>hyposalivation<br>thinning of the glomerular basement membrane		arthralgia<br>morning stiffness<br>inflammatory back pain<br>aphthous ulcerations of the oral cavity<br>tricolor pattern of discoloration of the fingers and toes<br>venous thrombosis<br>proof of miscarriage<br>dry eye<br>hepatomegaly<br>anti-SSA autoantibodies<br>anti-La (SS-B)-autoantibodies<br>mumps virus<br>confirmed human immunodeficiency virus infection<br>Epstein-Barr virus<br>splenomegaly<br>ACE level<br>hilar lymphadenopathy<br>pulmonary nodules<br>Interleukin 2 receptor, elevated<br>granuloma
**56**							
**Visit**	Sex	Age	Confirmed Diagnoses	Present Risk Factors	Pathological Symptoms/Findings (with attributes)	Absent Risk Factors	Non-pathological Symptoms/Findings
**Top Five Fit, Top Fit**	Female	58		smoker	high blood pressure (time since onset: one month to one year)<br>cholesterol level (result: elevated)<br>triglyceride level (result: elevated)<br>double vision (time since onset: one month to one year)<br>oculomotor nerve palsy (time since onset: one month to one year)<br>sinusitis<br>exophthalmos (laterality: unilateral)<br>swelling of the eyelid<br>glomerular filtration rate, reduced<br>orbital mass		CRP, elevated<br>orbital fracture<br>thrombosis, venous sinus
**Diagnosis**	Female	58	IgG4-related disease	smoker	high blood pressure (time since onset: one month to one year)<br>cholesterol level (result: elevated)<br>triglyceride level (result: elevated)<br>double vision (time since onset: one month to one year)<br>oculomotor nerve palsy (time since onset: one month to one year)<br>sinusitis<br>exophthalmos (laterality: unilateral time since onset: one month to one year)<br>swelling of the eyelid (time since onset: one month to one year)<br>glomerular filtration rate, reduced<br>orbital mass<br>hepatic mass seen on imaging<br>left ventricular hypertrophy<br>mitral valve regurgitation on imaging<br>aortic valve regurgitation on imaging<br>tricuspid valve regurgitation on imaging<br>predominance of IgG4 positive plasma cells in histopathology	Hx: eye trauma	CRP, elevated<br>orbital fracture<br>vitamin D level, decreased<br>thyroid peroxidase antibodies<br>thyrotropin receptor antibodies<br>thyroglobulin antibodies<br>ACTH level<br>antineutrophil cytoplasmic antibodies present<br>anti-GBM antibodies present<br>IgG4 level<br>thrombosis, venous sinus<br>free T4<br>thyroid stimulating hormone level<br>elevated rheumatoid factor<br>cardiac ejection fraction<br>positive interferon gamma release assay for tuberculosis
**57**							
**Visit**	Sex	Age	Confirmed Diagnoses	Present Risk Factors	Pathological Symptoms/Findings (with attributes)	Absent Risk Factors	Non-pathological Symptoms/Findings
**Top Five Fit, Top Fit**	Female	58			lipoprotein a level, elevated<br>cholesterol level (result: elevated)<br>antinuclear antibody level, elevated<br>myalgia (time since onset: one month to one year)<br>upper back pain (time since onset: one month to one year)<br>wedging of vertebral body<br>osteoporosis<br>lower back pain (time since onset: one month to one year)<br>rib fracture<br>arthralgia	Hx: spinal trauma	creatine kinase level, elevated
**Diagnosis**	Female	62	Hypophosphatasia		lipoprotein a level, elevated<br>cholesterol level (result: elevated)<br>antinuclear antibody level, elevated<br>myalgia (time since onset: more than one year)<br>upper back pain (time since onset: more than one year)<br>wedging of vertebral body<br>osteoporosis<br>alkaline phosphatase level (result: reduced)<br>lower back pain (time since onset: more than one year)<br>scoliosis<br>rib fracture<br>knee pain<br>ankle pain<br>muscle cramps in the lower extremity	Hx: spinal trauma	creatine kinase level, elevated<br>erythrocyte sedimentation rate<br>CRP, elevated<br>elevated rheumatoid factor<br>anti-cyclic citrullinated protein antibodies<br>anti-dsDNA antibodies<br>complement C3 level<br>complement C4 level<br>proteinase 3 antibody titer, elevated<br>myeloperoxidase antibodies present<br>anti-Scl-70
**58**							
**Visit**	Sex	Age	Confirmed Diagnoses	Present Risk Factors	Pathological Symptoms/Findings (with attributes)	Absent Risk Factors	Non-pathological Symptoms/Findings
**Diagnosis**	Female	44	Cryopyrin-associated periodic syndrome (CAPS)		fever (time since onset: one month to one year high grade: yes)<br>chills (time since onset: one month to one year)<br>hyperhidrosis, generalized (night sweats: yes time since onset: one month to one year)<br>reduced exercise tolerance (time since onset: one month to one year)<br>arthralgia (time since onset: one month to one year)<br>knee pain (time since onset: one month to one year)<br>erythrocyte sedimentation rate (result: reduced)<br>anemia<br>thrombocytosis<br>leukocytosis<br>CRP, elevated<br>calcium level (result: elevated)<br>alpha 2 globulin level (result: elevated)<br>gamma gt level, elevated<br>alkaline phosphatase level (result: elevated)<br>alanine transaminase level, elevated<br>hepatomegaly<br>paraaortic lymphadenopathy<br>myalgia (time since onset: one month to one year)<br>sore throat<br>gallstone<br>enlarged thyroid gland<br>serum free light chain level, elevated<br>cervical lymphadenopathy<br>bowel wall thickening<br>arthritis<br>NLRP3 gene mutation		malignant cells in bone marrow<br>lymphoblastic hyperplasia<br>myeloid hyperplasia<br>ACE level<br>immune deposits<br>anti-dsDNA antibodies<br>liver abscess<br>increased hepatic echogenicity<br>hilar lymphadenopathy<br>pulmonary nodules<br>pulmonary infiltrates<br>pleural effusion<br>antinuclear antibody level, elevated<br>ferritin level<br>antistreptolysin O antibody titer, elevated<br>swelling, joint<br>tricolor pattern of discoloration of the fingers and toes<br>dry mouth<br>dry eye<br>uveitis<br>stenosis of the carotid artery<br>stenosis of the subclavian artery<br>MEFV gene mutation<br>TNFRSF1A gene mutation
**59**							
**Visit**	Sex	Age	Confirmed Diagnoses	Present Risk Factors	Pathological Symptoms/Findings (with attributes)	Absent Risk Factors	Non-pathological Symptoms/Findings
**Top Five Fit, Top Fit (<= 1 month early)**	Male	28			lower back pain<br>PIP joint pain (finger) (laterality: unilateral)<br>sacroiliitis<br>fever (high grade: no)<br>CRP, elevated<br>hyperuricemia<br>renal insufficiency<br>anemia		malaise
**Diagnosis**	Male	28	Gout arthritis		lower back pain (time since onset: one month to one year)<br>PIP joint pain (finger) (laterality: unilateral time since onset: one month to one year)<br>weight loss<br>sacroiliitis<br>swelling, elbow<br>urate crystals in synovial fluid<br>arthritis, MTP I joint<br>creatine kinase level, elevated<br>alkaline phosphatase level (result: elevated)<br>lactate dehydrogenase level, elevated<br>mean corpuscular volume (result: reduced)<br>parathyroid hormone level (result: elevated)<br>CRP, elevated<br>iron level (result: reduced)<br>ferritin level (result: reduced)<br>triglyceride level (result: elevated)<br>anemia<br>renal insufficiency		dyspnea<br>chest pain<br>fever<br>hyperuricemia<br>elevated rheumatoid factor<br>anti-cyclic citrullinated protein antibodies<br>antinuclear antibody level, elevated<br>antineutrophil cytoplasmic antibodies present<br>serum complement level<br>anti-Sm antibodies<br>anti-U1-RNP antibodies<br>anti-SSA autoantibodies<br>anti-La (SS-B)-autoantibodies<br>anti-Scl-70<br>anti-Jo 1 antibodies<br>abdominal tenderness
**60**							
**Visit**	Sex	Age	Confirmed Diagnoses	Present Risk Factors	Pathological Symptoms/Findings (with attributes)	Absent Risk Factors	Non-pathological Symptoms/Findings
**Top Five Fit**	Female	43			CRP, elevated<br>erythrocyte sedimentation rate (result: elevated)<br>hearing deficit (laterality: unilateral time since onset: more than one year)<br>vertigo (time since onset: more than one year)<br>fever (time since onset: more than one year high grade: no)<br>splenomegaly<br>elbow pain (time since onset: more than one year laterality: unilateral)<br>wrist pain (time since onset: more than one year)<br>hip pain (time since onset: more than one year laterality: unilateral)<br>foot pain (laterality: bilateral time since onset: more than one year)<br>knee pain (time since onset: more than one year)<br>free T3 (result: elevated)<br>complement C4 level (result: elevated)<br>calcium level (result: elevated)		myalgia<br>antinuclear antibody level, elevated<br>serum complement level<br>anti-Sm antibodies<br>anti-U1-RNP antibodies<br>anti-SSA autoantibodies<br>anti-La (SS-B)-autoantibodies<br>pathologically increased cryoglobulin level<br>anti-Scl-70<br>anti-Jo 1 antibodies<br>thyroid stimulating hormone level<br>parathyroid hormone level<br>IgG level<br>IgA level<br>IgM level<br>elevated rheumatoid factor<br>anti-cyclic citrullinated protein antibodies<br>antineutrophil cytoplasmic antibodies present<br>anticardiolipin antibodies, elevated<br>cardiac ejection fraction<br>heart valve defect<br>alkaline phosphatase level<br>facial pain<br>facial swelling<br>chest pain

**Diagnosis**	Female	44	Cryopyrin-associated periodic syndrome (CAPS)		CRP, elevated<br>elbow pain (time since onset: more than one year laterality: unilateral)<br>vertigo (time since onset: more than one year)<br>wrist pain (time since onset: more than one year)<br>hip pain (time since onset: more than one year laterality: unilateral)<br>foot pain (laterality: bilateral time since onset: more than one year)<br>knee pain (time since onset: more than one year)<br>hearing deficit (laterality: unilateral time since onset: more than one year)<br>free T3 (result: elevated)<br>IgM level (result: reduced)<br>complement C4 level (result: elevated)<br>calcium level (result: elevated)<br>fever (time since onset: more than one year high grade: no)<br>tachycardia on ECG<br>potassium level (result: elevated)<br>splenomegaly<br>complement C3 level (result: elevated)<br>NLRP3 gene mutation		erythrocyte sedimentation rate<br>myalgia<br>antinuclear antibody level, elevated<br>serum complement level<br>anti-Sm antibodies<br>anti-U1-RNP antibodies<br>anti-SSA autoantibodies<br>anti-La (SS-B)-autoantibodies<br>pathologically increased cryoglobulin level<br>anti-Scl-70<br>anti-Jo 1 antibodies<br>thyroid stimulating hormone level<br>parathyroid hormone level<br>IgG level<br>IgA level<br>elevated rheumatoid factor<br>anti-cyclic citrullinated protein antibodies<br>antineutrophil cytoplasmic antibodies present<br>anticardiolipin antibodies, elevated<br>cardiac ejection fraction<br>heart valve defect<br>anti-dsDNA antibodies<br>glomerular filtration rate, reduced<br>creatine kinase level, elevated<br>serum amyloid A, elevated<br>IgG4 level<br>facial pain<br>facial swelling<br>chest pain<br>alkaline phosphatase level
**61**							
**Visit**	Sex	Age	Confirmed Diagnoses	Present Risk Factors	Pathological Symptoms/Findings (with attributes)	Absent Risk Factors	Non-pathological Symptoms/Findings
**Top Five Fit, Top Fit**	Male	38		elevated BMI	dysuria (time since onset: more than one year)<br>abdominal pain (position: epigastric time since onset: one month to one year)<br>back pain (time since onset: one month to one year)<br>brain white matter lesion<br>sensory deficit, lower leg<br>pain of the oral cavity (time since onset: one month to one year)<br>painful dysesthesia of the trunk<br>impaired vision<br>muscle fasciculation<br>cerebrospinal fluid protein level (result: elevated)<br>creatine kinase level, elevated<br>serum creatinine level (result: elevated)<br>lactate dehydrogenase level, elevated		urethritis<br>testicular enlargement<br>scrotal mass<br>Treponema pallidum antigen<br>Lymphadenopathy on palpation<br>antinuclear antibody level, elevated<br>antimitochondrial antibody<br>antineutrophil cytoplasmic antibodies present<br>proteinase 3 antibody titer, elevated<br>myeloperoxidase antibodies present<br>meningism<br>cranial nerve disorder<br>lower extremity paresis<br>upper extremity paresis<br>muscle atrophy<br>muscular hypertonicity<br>hyperreflexic deep tendon reflexes
**Diagnosis**	Male	39	Behcet's disease	elevated BMI	dysuria (time since onset: more than one year)<br>abdominal pain (position: epigastric time since onset: one month to one year)<br>back pain (time since onset: one month to one year)<br>brain white matter lesion<br>pain of the oral cavity (time since onset: one month to one year)<br>impaired vision (time since onset: one month to one year)<br>muscle fasciculation (time since onset: one month to one year)<br>cerebrospinal fluid protein level (result: elevated)<br>creatine kinase level, elevated<br>lactate dehydrogenase level, elevated<br>spinal stenosis seen on imaging<br>arthritis<br>uveitis<br>fatigue<br>aphthous ulcerations of the oral cavity<br>balanitis<br>swelling, ankle joint (laterality: unilateral)<br>gamma gt level, elevated<br>alanine transaminase level, elevated<br>high blood pressure<br>hematuria<br>epistaxis (time since onset: one month to one year)<br>foot dorsiflexor paresis<br>myalgia (time since onset: one month to one year)<br>tenderness, MTP joints (laterality: unilateral)		urethritis<br>testicular enlargement<br>scrotal mass<br>Treponema pallidum antigen<br>Lymphadenopathy on palpation<br>antinuclear antibody level, elevated<br>antimitochondrial antibody<br>antineutrophil cytoplasmic antibodies present<br>proteinase 3 antibody titer, elevated<br>myeloperoxidase antibodies present<br>meningism<br>cranial nerve disorder<br>lower extremity paresis<br>upper extremity paresis<br>muscle atrophy<br>muscular hypertonicity<br>hyperreflexic deep tendon reflexes<br>serum creatinine level<br>CRP, elevated<br>HLA-B27 positive<br>HLA-B51 positive<br>anti-dsDNA antibodies<br>leukocyte level<br>erythema<br>Enterocolitis<br>sensory deficit<br>painful dysesthesia<br>elevated skin temperature of the ankle<br>skin rash<br>hepatomegaly<br>splenomegaly<br>borrelia burgdorferi antibodies in CSF
**62**							
**Visit**	Sex	Age	Confirmed Diagnoses	Present Risk Factors	Pathological Symptoms/Findings (with attributes)	Absent Risk Factors	Non-pathological Symptoms/Findings
**Top Five Fit, Top Fit**	Female	27			erosion and ulceration of the lower extremity (pain: yes crusted surface: yes time since onset: more than one year)<br>paresthesia of the lower extremity<br>macule on the lower extremity (purple: yes uneven borders: yes pain: yes time since onset: more than one year)<br>livedo (time since onset: more than one year)		
**Diagnosis**	Female	30	Panarteritis nodosa		erosion and ulceration of the lower extremity (pain: yes crusted surface: yes time since onset: more than one year)<br>paresthesia of the lower extremity<br>macule on the lower extremity (purple: yes uneven borders: yes pain: yes time since onset: more than one year)<br>livedo (time since onset: more than one year)<br>CRP, elevated<br>erythrocyte sedimentation rate (result: elevated)<br>creatine kinase level, elevated<br>INR level (result: elevated)<br>microhematuria<br>complement C4 level (result: reduced)		antinuclear antibody level, elevated<br>anti-SSA autoantibodies<br>anti-La (SS-B)-autoantibodies<br>complement C3 level<br>pathologically increased cryoglobulin level<br>anti-dsDNA antibodies<br>antineutrophil cytoplasmic antibodies present
**63**							
**Visit**	Sex	Age	Confirmed Diagnoses	Present Risk Factors	Pathological Symptoms/Findings (with attributes)	Absent Risk Factors	Non-pathological Symptoms/Findings
**Top Five Fit, Top Fit**	Female	37			swelling, wrist (time since onset: one month to one year)<br>wrist pain (time since onset: one month to one year)<br>shoulder pain (time since onset: one month to one year)<br>gluteal pain (time since onset: one month to one year laterality: unilateral)<br>nightly awakening (adults)<br>urinary tract infection<br>sacroiliitis<br>erythrocyte sedimentation rate (result: elevated)<br>CRP, elevated<br>HLA-B27 positive<br>microcytic anemia<br>arthritis, wrist<br>enthesitis		diarrhea<br>uveitis<br>serum complement level<br>elevated rheumatoid factor<br>antinuclear antibody level, elevated<br>anticardiolipin antibodies, elevated<br>plaque of the skin<br>erythema
**Diagnosis**	Female	53	Spondyloarthritis		swelling, wrist (time since onset: more than one year)<br>wrist pain (time since onset: more than one year)<br>shoulder pain (time since onset: more than one year)<br>gluteal pain (time since onset: more than one year laterality: unilateral)<br>nightly awakening (adults)<br>lower back pain (nocturnal or early morning: exacerbates time since onset: more than one year)<br>urinary tract infection<br>sacroiliitis<br>HLA-B27 positive<br>microcytic anemia<br>arthritis, wrist<br>enthesitis<br>leukopenia<br>knee pain (time since onset: more than one year)<br>morning stiffness (time since onset: one month to one year)<br>hand pain<br>paresthesia of the hand<br>headache (time since onset: more than one year)<br>dizziness<br>sensory deficit, lower extremity<br>spinal stenosis seen on imaging<br>tendinitis<br>serum glucose level (result: reduced)<br>BNP in serum, elevated<br>complement C3 level (result: reduced)		diarrhea<br>uveitis<br>erythrocyte sedimentation rate<br>CRP, elevated<br>serum complement level<br>elevated rheumatoid factor<br>antinuclear antibody level, elevated<br>anticardiolipin antibodies, elevated<br>plaque of the skin<br>erythema<br>glomerular filtration rate, reduced<br>anti-dsDNA antibodies<br>anti-GBM antibodies present<br>anti-Ku antibodies<br>anti-PL 7 antibodies<br>anti-PL 12 antibodies<br>anti-SRP antibodies<br>anti-Jo 1 antibodies<br>anti-PmScl antibody titer<br>anti-OJ antibodies<br>anti-EJ antibodies<br>anti-alpha-fodrin antibodies<br>serum amyloid A, elevated<br>aphthous ulcerations of the oral cavity<br>venous thrombosis<br>chest pain<br>peripheral edema (pitting)
**64**							
**Visit**	Sex	Age	Confirmed Diagnoses	Present Risk Factors	Pathological Symptoms/Findings (with attributes)	Absent Risk Factors	Non-pathological Symptoms/Findings
**Top Five Fit**	Male	21			palpitation (time since onset: one month to one year)<br>rapid pulse (time since onset: one month to one year)<br>tachycardia on ECG<br>swelling of the hand (time since onset: one month to one year laterality: bilateral)<br>acne<br>ankle pain (time since onset: more than one year alcohol: exacerbates)<br>knee pain (laterality: bilateral alcohol: exacerbates time since onset: one month to one year)<br>hand pain (time since onset: one month to one year laterality: bilateral)<br>tenderness, finger joint (time since onset: one month to one year laterality: bilateral)<br>left ventricular hypertrophy<br>alpha-galactosidase A enzyme activity, decreased		heart failure<br>syncope<br>heart valve defect<br>elevated rheumatoid factor<br>anti-cyclic citrullinated protein antibodies<br>antinuclear antibody level, elevated<br>erythrocyte sedimentation rate<br>anti-dsDNA antibodies<br>anti-SSA autoantibodies<br>anti-La (SS-B)-autoantibodies<br>anti-Scl-70<br>skin rash<br>erythema<br>plaque of the skin<br>bone erosion of the finger joint<br>anemia<br>leukocyte level<br>platelet count
**Top Fit**	Male	21			palpitation (time since onset: one month to one year)<br>rapid pulse (time since onset: one month to one year)<br>tachycardia on ECG<br>swelling of the hand (time since onset: one month to one year laterality: bilateral)<br>acne<br>ankle pain (time since onset: more than one year alcohol: exacerbates)<br>knee pain (laterality: bilateral alcohol: exacerbates time since onset: one month to one year)<br>hand pain (time since onset: one month to one year laterality: bilateral)<br>tenderness, finger joint (time since onset: one month to one year laterality: bilateral)<br>left ventricular hypertrophy<br>alpha-galactosidase A enzyme activity, decreased<br>GLA gene mutation		heart failure<br>syncope<br>heart valve defect<br>elevated rheumatoid factor<br>anti-cyclic citrullinated protein antibodies<br>antinuclear antibody level, elevated<br>erythrocyte sedimentation rate<br>anti-dsDNA antibodies<br>anti-SSA autoantibodies<br>anti-La (SS-B)-autoantibodies<br>anti-Scl-70<br>skin rash<br>erythema<br>plaque of the skin<br>bone erosion of the finger joint<br>anemia<br>leukocyte level<br>platelet count

**Diagnosis**	Male	21	Fabry disease		palpitation (time since onset: one month to one year)<br>rapid pulse (time since onset: one month to one year)<br>tachycardia on ECG<br>swelling of the hand (time since onset: one month to one year laterality: bilateral)<br>acne<br>ankle pain (time since onset: more than one year alcohol: exacerbates)<br>knee pain (laterality: bilateral alcohol: exacerbates time since onset: one month to one year)<br>hand pain (time since onset: one month to one year laterality: bilateral)<br>tenderness, finger joint (time since onset: one month to one year laterality: bilateral)<br>left ventricular hypertrophy<br>alpha-galactosidase A enzyme activity, decreased<br>papule in the periumbilical region<br>GLA gene mutation		heart failure<br>syncope<br>heart valve defect<br>elevated rheumatoid factor<br>anti-cyclic citrullinated protein antibodies<br>antinuclear antibody level, elevated<br>erythrocyte sedimentation rate<br>anti-dsDNA antibodies<br>anti-SSA autoantibodies<br>anti-La (SS-B)-autoantibodies<br>anti-Scl-70<br>skin rash<br>erythema<br>plaque of the skin<br>bone erosion of the finger joint<br>anemia<br>leukocyte level<br>platelet count
**65**							
**Visit**	Sex	Age	Confirmed Diagnoses	Present Risk Factors	Pathological Symptoms/Findings (with attributes)	Absent Risk Factors	Non-pathological Symptoms/Findings
**Top Five Fit**	Male	20		Hx: recent infection<br>smoker	diarrhea<br>hypothyroidism<br>proteinuria (intensity: trace to mild)<br>serum creatinine level (result: elevated)<br>glomerular filtration rate, reduced<br>small kidneys (laterality: bilateral)<br>vitamin D level, decreased<br>hyperkalemia<br>blood urea nitrogen level (result: elevated)<br>serum albumin level (result: reduced)<br>serum protein level (result: reduced)<br>focal segmental glomerulosclerosis<br>renal insufficiency	diabetes mellitus<br>Hx: hypertension	fever<br>vomiting<br>hematochezia<br>antinuclear antibody level, elevated<br>anti-dsDNA antibodies<br>serum complement level<br>antineutrophil cytoplasmic antibodies present<br>schistocytes<br>amyloid protein deposition<br>immune deposits<br>leukocyte level<br>platelet count<br>anemia
**Diagnosis**	Male	21	Fabry disease	Hx: recent infection<br>smoker	diarrhea<br>hypothyroidism<br>proteinuria (intensity: trace to mild)<br>serum creatinine level (result: elevated)<br>glomerular filtration rate, reduced<br>small kidneys (laterality: bilateral)<br>hyperkalemia<br>blood urea nitrogen level (result: elevated)<br>serum albumin level (result: reduced)<br>serum protein level (result: reduced)<br>focal segmental glomerulosclerosis<br>renal insufficiency<br>anemia<br>parathyroid hormone level (result: elevated)<br>papule on the trunk<br>GLA gene mutation	diabetes mellitus<br>Hx: hypertension	fever<br>vomiting<br>hematochezia<br>antinuclear antibody level, elevated<br>anti-dsDNA antibodies<br>serum complement level<br>antineutrophil cytoplasmic antibodies present<br>schistocytes<br>amyloid protein deposition<br>immune deposits<br>vitamin D level, decreased<br>leukocyte level<br>platelet count<br>hepatitis C virus<br>confirmed human immunodeficiency virus infection
**66**							
**Visit**	Sex	Age	Confirmed Diagnoses	Present Risk Factors	Pathological Symptoms/Findings (with attributes)	Absent Risk Factors	Non-pathological Symptoms/Findings
**Top Five Fit**	Female	50			vomiting (time since onset: more than one year)<br>cholesterol level (result: elevated)<br>steatosis hepatis<br>lower back pain (time since onset: more than one year)<br>finger joint pain (time since onset: more than one year)<br>tricolor pattern of discoloration of the fingers and toes (time since onset: more than one year)<br>diarrhea (time since onset: more than one year)<br>groin pain (time since onset: more than one year)<br>swelling, finger joints (time since onset: more than one year)<br>morning stiffness (time since onset: more than one year)<br>shoulder pain (time since onset: more than one year)<br>neck pain (time since onset: more than one year)<br>insomnia<br>rapid pulse<br>hyperhidrosis, generalized<br>Hepatitis A virus<br>ferritin level (result: elevated)<br>triglyceride level (result: elevated)<br>cerebral atrophy<br>lipase level, elevated<br>perirenal fluid collections<br>bilirubin level, elevated<br>CRP, elevated<br>pleural effusion<br>pleuritis<br>ascites<br>renal cysts<br>nausea<br>proof of acute pancreatitis<br>ovarian cyst seen on imaging<br>IgM level (result: elevated)<br>gamma gt level, elevated	alcohol abuse	antinuclear antibody level, elevated<br>antimitochondrial antibody<br>ceruloplasmin level<br>Hepatitis B virus<br>hepatitis C virus<br>Lyme-specific IgM level, elevated<br>Lyme-specific IgG level, elevated<br>vitamin B12 level<br>iron level<br>leukocytosis<br>IgG level<br>anti-smooth-muscle antibodies<br>antineutrophil cytoplasmic antibodies present
**Diagnosis**	Female	53	Antisynthetase Syndrome		vomiting (time since onset: more than one year)<br>cholesterol level (result: elevated)<br>steatosis hepatis<br>lower back pain (time since onset: more than one year)<br>finger joint pain (time since onset: more than one year)<br>tricolor pattern of discoloration of the fingers and toes (time since onset: more than one year)<br>diarrhea (time since onset: more than one year)<br>groin pain (time since onset: more than one year)<br>swelling, finger joints (time since onset: more than one year)<br>morning stiffness (time since onset: more than one year)<br>shoulder pain (time since onset: more than one year)<br>neck pain (time since onset: more than one year)<br>insomnia<br>rapid pulse (time since onset: more than one year)<br>hyperhidrosis, generalized<br>Hepatitis A virus<br>ferritin level (result: elevated)<br>iron level (result: reduced)<br>triglyceride level (result: elevated)<br>cerebral atrophy<br>lipase level, elevated<br>perirenal fluid collections<br>bilirubin level, elevated<br>CRP, elevated<br>pleural effusion<br>pleuritis<br>ascites<br>renal cysts<br>nausea<br>proof of acute pancreatitis<br>ovarian cyst seen on imaging<br>IgM level (result: elevated)<br>gamma gt level, elevated<br>reduced exercise tolerance (time since onset: one month to one year)<br>elevated rheumatoid factor<br>anti-Jo 1 antibodies<br>complement C3 level (result: elevated)<br>uric acid level (result: elevated)<br>nocturia<br>erythema of the cheeks<br>tenderness, finger joint<br>swelling, MCP joints<br>elevated skin temperature of the MTP joint<br>myalgia (time since onset: one month to one year)<br>dyspnea (time since onset: one month to one year)<br>IgG4 level (result: elevated)<br>anticardiolipin antibodies, elevated<br>beta 2 microglobulin level, elevated<br>vitamin D level, decreased<br>peripheral polyneuropathy<br>hepatic mass seen on imaging<br>renal mass seen on imaging	alcohol abuse	antinuclear antibody level, elevated<br>antimitochondrial antibody<br>ceruloplasmin level<br>Hepatitis B virus<br>hepatitis C virus<br>Lyme-specific IgM level, elevated<br>Lyme-specific IgG level, elevated<br>vitamin B12 level<br>leukocytosis<br>IgG level<br>anti-smooth-muscle antibodies<br>antineutrophil cytoplasmic antibodies present<br>anti-dsDNA antibodies<br>anti-cyclic citrullinated protein antibodies<br>thrombocytopenia<br>heart valve defect<br>anti-SSA autoantibodies<br>anti-La (SS-B)-autoantibodies<br>anti-U1-RNP antibodies<br>anti-Sm antibodies<br>anti-Scl-70<br>alpha-1 antitrypsin level, decreased<br>positive interferon gamma release assay for tuberculosis<br>lupus erythematosus
**67**							
**Visit**	Sex	Age	Confirmed Diagnoses	Present Risk Factors	Pathological Symptoms/Findings (with attributes)	Absent Risk Factors	Non-pathological Symptoms/Findings
**Diagnosis**	Male	50	CREST syndrome	Hx: hypertension<br>immunocompromised	focal segmental glomerulosclerosis<br>uric acid level (result: elevated)<br>carpal tunnel syndrome<br>pain in the upper arm (laterality: bilateral time since onset: one month to one year)<br>thigh pain (laterality: bilateral time since onset: one month to one year)<br>neck pain (time since onset: one month to one year)<br>diarrhea<br>shoulder pain (time since onset: one month to one year)<br>swelling, shoulder (time since onset: one month to one year)<br>antinuclear antibody level, elevated<br>anti-centromere antibodies<br>erythrocyte sedimentation rate (result: elevated)<br>hemoglobin level (result: reduced)<br>thrombocytopenia<br>potassium level (result: elevated)<br>phosphate level (result: elevated)<br>serum creatinine level (result: elevated)<br>blood urea nitrogen level (result: elevated)<br>glomerular filtration rate, reduced<br>PSA level, elevated<br>ferritin level (result: elevated)<br>serum complement level (result: elevated)<br>parathyroid hormone level (result: elevated)<br>steatosis hepatis<br>liver cyst<br>small kidneys<br>splenomegaly<br>nephrocalcinosis<br>Interleukin 2 receptor, elevated		anti-dsDNA antibodies<br>anti-Sm antibodies<br>anti-SSA autoantibodies<br>anti-La (SS-B)-autoantibodies<br>anti-U1-RNP antibodies<br>anti-Scl-70<br>anti-Jo 1 antibodies
**68**							
**Visit**	Sex	Age	Confirmed Diagnoses	Present Risk Factors	Pathological Symptoms/Findings (with attributes)	Absent Risk Factors	Non-pathological Symptoms/Findings
**Top Five Fit, Top Fit**	Female	45		immunocompromised<br>Hx: hypertension	wrist pain (time since onset: more than one year laterality: unilateral)<br>swelling, wrist (time since onset: more than one year)<br>finger joint pain (time since onset: more than one year laterality: bilateral)<br>swelling, finger joints (time since onset: more than one year)<br>ankle pain (time since onset: more than one year laterality: unilateral)<br>swelling, ankle joint (time since onset: more than one year laterality: unilateral)<br>uric acid level (result: elevated)<br>headache<br>joint space narrowing of the fingers		

**Diagnosis**	Female	45	Gout arthritis	immunocompromised<br>Hx: hypertension	wrist pain (time since onset: more than one year laterality: unilateral)<br>swelling, wrist (time since onset: more than one year)<br>ankle pain (time since onset: more than one year laterality: unilateral)<br>swelling, ankle joint (time since onset: more than one year laterality: unilateral)<br>uric acid level (result: elevated)<br>headache (time since onset: one month to one year)<br>joint space narrowing of the fingers<br>lower back pain (time since onset: one month to one year)<br>MCP joint pain (time since onset: one month to one year)<br>swelling, MCP joints (time since onset: one month to one year)	diabetes mellitus	sensory deficit, hand<br>antinuclear antibody level, elevated<br>elevated rheumatoid factor
**69**							
**Visit**	Sex	Age	Confirmed Diagnoses	Present Risk Factors	Pathological Symptoms/Findings (with attributes)	Absent Risk Factors	Non-pathological Symptoms/Findings
**Top Five Fit**	Male	57		elevated BMI	nausea (time since onset: more than one year)<br>rapid pulse (time since onset: more than one year)<br>chest pain (position: unilateral time since onset: more than one year sharp: yes)<br>aural fullness (time since onset: more than one year)<br>hyperhidrosis, generalized (cold perspiration: yes time since onset: more than one year)<br>headache (time since onset: more than one year)<br>vertigo (time since onset: more than one year)<br>upper extremity pain (laterality: unilateral time since onset: more than one year)<br>neck pain (time since onset: more than one year)<br>high blood pressure (time since onset: more than one year)<br>lower back pain (time since onset: more than one year activity: exacerbates nocturnal or early morning: exacerbates)<br>sensory deficit, hand (distribution: bilateral, symmetrical time since onset: more than one year)<br>painful dysesthesia of the upper extremity (distribution: symmetrical time since onset: more than one year)<br>painful dysesthesia of the lower extremity (distribution: symmetrical time since onset: more than one year)<br>paresthesia of the hand (distribution: symmetrical time since onset: more than one year)<br>paresthesia of the lower extremity (distribution: symmetrical time since onset: more than one year)<br>sensory deficit, foot (distribution: bilateral, symmetrical reduced vibration sense: yes impaired proprioception: yes time since onset: more th<br>Premature ventricular contraction<br>ventricular tachycardia<br>aortic valve regurgitation on imaging<br>dyspnea (severity: on exertion time since onset: more than one year)<br>LDL level (result: elevated)<br>left axis deviation<br>right bundle branch block<br>gait impairment (time since onset: more than one year)<br>spinal tenderness<br>positive straight leg raise<br>lower extremity pain (time since onset: more than one year)<br>insomnia (time since onset: more than one year)<br>reduced exercise tolerance (time since onset: more than one year)<br>peripheral polyneuropathy<br>carpal tunnel syndrome<br>panic attacks (time since onset: more than one year)<br>depressed mood (time since onset: one month to one year)<br>impaired vision (time since onset: one month to one year)<br>heat intolerance (time since onset: one month to one year)<br>tenderness, MTP joints (laterality: bilateral time since onset: one month to one year)<br>limited range of motion of the spine (time since onset: one month to one year)<br>ferritin level (result: elevated)<br>vitamin D level, decreased<br>groin pain<br>hip pain	alcohol abuse	upper extremity paresis<br>lower extremity paresis<br>facial nerve palsy<br>impaired fine motor skills<br>hyperreflexia, upper limb<br>hyperreflexia, lower limb<br>diminished reflexes, upper limb<br>diminished reflexes, lower limb<br>spinal stenosis seen on imaging<br>disc herniation<br>urinary incontinence<br>fecal incontinence<br>cardiac ejection fraction<br>serum cardiac troponin level, elevated<br>shortened PR interval on ECG<br>prolonged PR interval on ECG<br>shortened QT interval on ECG<br>prolonged QT interval on ECG<br>CRP, elevated<br>heart wall motion abnormality<br>ST segment changes on ECG<br>thyroid stimulating hormone level<br>calcium level<br>phosphate level<br>normetanephrine level in urine, elevated<br>swelling, joint
**Diagnosis**	Male	58	Spondyloarthritis	elevated BMI	nausea (time since onset: more than one year)<br>rapid pulse (time since onset: more than one year)<br>chest pain (position: unilateral time since onset: more than one year sharp: yes)<br>aural fullness (time since onset: more than one year)<br>hyperhidrosis, generalized (cold perspiration: yes time since onset: more than one year)<br>headache (time since onset: more than one year)<br>vertigo (time since onset: more than one year)<br>upper extremity pain (laterality: unilateral time since onset: more than one year)<br>neck pain (time since onset: more than one year)<br>high blood pressure (time since onset: more than one year)<br>lower back pain (time since onset: more than one year activity: exacerbates nocturnal or early morning: exacerbates)<br>sensory deficit, hand (distribution: bilateral, symmetrical time since onset: more than one year)<br>painful dysesthesia of the upper extremity (distribution: symmetrical time since onset: more than one year)<br>painful dysesthesia of the lower extremity (distribution: symmetrical time since onset: more than one year)<br>paresthesia of the hand (distribution: symmetrical time since onset: more than one year)<br>paresthesia of the lower extremity (distribution: symmetrical time since onset: more than one year)<br>sensory deficit, foot (distribution: bilateral, symmetrical reduced vibration sense: yes impaired proprioception: yes time since onset: more th<br>Premature ventricular contraction<br>ventricular tachycardia<br>aortic valve regurgitation on imaging<br>dyspnea (severity: on exertion time since onset: more than one year)<br>LDL level (result: elevated)<br>left axis deviation<br>right bundle branch block<br>gait impairment (time since onset: more than one year)<br>spinal tenderness<br>positive straight leg raise<br>CRP, elevated<br>lower extremity pain (time since onset: more than one year)<br>insomnia (time since onset: more than one year)<br>reduced exercise tolerance (time since onset: more than one year)<br>peripheral polyneuropathy<br>carpal tunnel syndrome<br>panic attacks (time since onset: more than one year)<br>depressed mood (time since onset: one month to one year)<br>impaired vision (time since onset: one month to one year)<br>heat intolerance (time since onset: one month to one year)<br>tenderness, MTP joints (laterality: bilateral time since onset: one month to one year)<br>limited range of motion of the spine (time since onset: one month to one year)<br>ferritin level (result: elevated)<br>vitamin D level, decreased<br>groin pain (time since onset: one month to one year)<br>hip pain (time since onset: one month to one year)<br>diarrhea (time since onset: one month to one year)<br>constipation (time since onset: one month to one year)<br>intestinal meteorism<br>reduced nerve fiber density<br>transaminase levels, elevated<br>creatine kinase level, elevated<br>splenomegaly<br>steatosis hepatis<br>intestinal inflammation<br>gastric mucosal inflammation<br>sacroiliitis<br>inflammatory back pain	alcohol abuse	upper extremity paresis<br>lower extremity paresis<br>facial nerve palsy<br>impaired fine motor skills<br>hyperreflexia, upper limb<br>hyperreflexia, lower limb<br>diminished reflexes, upper limb<br>diminished reflexes, lower limb<br>spinal stenosis seen on imaging<br>disc herniation<br>urinary incontinence<br>fecal incontinence<br>cardiac ejection fraction<br>serum cardiac troponin level, elevated<br>shortened PR interval on ECG<br>prolonged PR interval on ECG<br>shortened QT interval on ECG<br>prolonged QT interval on ECG<br>heart wall motion abnormality<br>ST segment changes on ECG<br>thyroid stimulating hormone level<br>calcium level<br>phosphate level<br>normetanephrine level in urine, elevated<br>swelling, joint<br>transferrin level<br>hepatitis C virus<br>Hepatitis B virus<br>Hepatitis A virus<br>monoclonal gammopathy<br>amyloid protein deposition<br>serum amyloid A, elevated<br>HLA-B27 positive<br>GLA gene mutation<br>Tropheryma whipplei
**70**							
**Visit**	Sex	Age	Confirmed Diagnoses	Present Risk Factors	Pathological Symptoms/Findings (with attributes)	Absent Risk Factors	Non-pathological Symptoms/Findings
**Top Five Fit, Top Fit (<= 1 month early)**	Female	50			renal insufficiency<br>osteopenia<br>microcytic anemia<br>swelling of the nose (time since onset: more than one year)<br>erythema of the nose (time since onset: more than one year)<br>erythema of the ear (time since onset: more than one year)<br>ear swelling<br>episcleritis<br>necrosis of the fingers (time since onset: more than one year)<br>necrosis of the toes (time since onset: more than one year)<br>impaired vision (time since onset: one month to one year laterality: unilateral)<br>shoulder pain (time since onset: one month to one year laterality: bilateral)<br>foot pain (time since onset: more than one year laterality: bilateral)		
**Diagnosis**	Female	50	Relapsing Polychondritis		renal insufficiency<br>osteopenia<br>microcytic anemia<br>swelling of the nose (time since onset: more than one year)<br>erythema of the nose (time since onset: more than one year)<br>erythema of the ear (time since onset: more than one year)<br>ear swelling<br>episcleritis<br>necrosis of the fingers (time since onset: more than one year)<br>necrosis of the toes (time since onset: more than one year)<br>impaired vision (time since onset: one month to one year laterality: unilateral)<br>shoulder pain (time since onset: one month to one year laterality: bilateral)<br>foot pain (time since onset: more than one year laterality: bilateral)<br>swelling, wrist<br>swelling of the foot (laterality: unilateral)<br>swelling, MCP I joint<br>muscular atrophy of the upper limb<br>reduced exercise tolerance<br>eosinophilia in blood<br>leukocytosis		
**71**							
**Visit**	Sex	Age	Confirmed Diagnoses	Present Risk Factors	Pathological Symptoms/Findings (with attributes)	Absent Risk Factors	Non-pathological Symptoms/Findings
**Diagnosis**	Female	49	Relapsing Polychondritis	Hx: hypertension	renal insufficiency<br>heart failure<br>Lymphadenopathy on examination<br>elbow pain<br>sternal tenderness<br>heel pain<br>arthritis, MCP joints (time since onset: more than one year)<br>left bundle branch block<br>aortic valve regurgitation on imaging<br>sacroiliitis<br>microhematuria<br>CRP, elevated<br>HLA-B27 positive<br>reduced exercise tolerance<br>rapid pulse<br>palpitation (time since onset: one month to one year)<br>pleural effusion<br>lower back pain (time since onset: more than one year)<br>foot pain (time since onset: more than one year)<br>ankle pain (time since onset: more than one year)<br>myalgia<br>erythema of the ear (time since onset: one month to one year pain: yes bilateral distribution: no)<br>nasal pain (time since onset: one month to one year)<br>erythema of the nose<br>aphthous ulcerations of the oral cavity<br>fatigue<br>ear swelling (bilateral distribution: no)<br>uric acid level (result: elevated)<br>erythrocyte sedimentation rate (result: elevated)<br>enthesitis<br>malaise		syncope<br>chest pain
**72**							
**Visit**	Sex	Age	Confirmed Diagnoses	Present Risk Factors	Pathological Symptoms/Findings (with attributes)	Absent Risk Factors	Non-pathological Symptoms/Findings

**Top Five Fit**	Female	44			palmoplantar pustulosis		
**Top Fit**	Female	54			palmoplantar pustulosis (time since onset: more than one year)<br>jaw swelling<br>pain in lower jaw<br>erythrocyte sedimentation rate (result: elevated)<br>bone lesion<br>Hyperostosis		antinuclear antibody level, elevated<br>anti-dsDNA antibodies<br>antineutrophil cytoplasmic antibodies present<br>anti-U1-RNP antibodies<br>anti-SSA autoantibodies<br>anti-La (SS-B)-autoantibodies<br>anti-Sm antibodies<br>anti-Jo 1 antibodies<br>anti-Scl-70
**Diagnosis**	Female	57	SAPHO Syndrome		palmoplantar pustulosis (time since onset: more than one year)<br>jaw swelling (time since onset: one month to one year)<br>pain in lower jaw (time since onset: one month to one year)<br>erythrocyte sedimentation rate (result: elevated)<br>bone lesion<br>osteomyelitis<br>CRP, elevated<br>leukocytosis<br>Hyperostosis		antinuclear antibody level, elevated<br>anti-dsDNA antibodies<br>antineutrophil cytoplasmic antibodies present<br>anti-U1-RNP antibodies<br>anti-SSA autoantibodies<br>anti-La (SS-B)-autoantibodies<br>anti-Sm antibodies<br>anti-Jo 1 antibodies<br>anti-Scl-70
**73**							
**Visit**	Sex	Age	Confirmed Diagnoses	Present Risk Factors	Pathological Symptoms/Findings (with attributes)	Absent Risk Factors	Non-pathological Symptoms/Findings
**Diagnosis**	Male	35	Behcet's disease	smoker	lower back pain (time since onset: more than one year radiating down the leg: yes)<br>neck pain (time since onset: more than one year)<br>extremity pain (at motion: exacerbates time since onset: more than one year laterality: bilateral)<br>leukocyte level (result: elevated)<br>lymphocytosis<br>creatine kinase level, elevated<br>nasal septum deviation<br>sinusitis<br>eosinophilia in blood<br>chest pain (respiration: exacerbates position: unilateral time since onset: more than one year)<br>headache (laterality: bilateral time since onset: more than one year)<br>bone pain (time since onset: more than one year)<br>fatigue (time since onset: more than one year)<br>kidney stone<br>myalgia (time since onset: more than one year)<br>nausea (time since onset: one month to one year)<br>vertigo (time since onset: more than one year)<br>double vision (time since onset: more than one year)<br>sensory deficit, face (distribution: unilateral time since onset: more than one year)<br>Sensory deficit of the trunk (distribution: unilateral time since onset: more than one year)<br>impaired vision (laterality: unilateral time since onset: more than one year)<br>dry eye (time since onset: more than one year)<br>ear noise (time since onset: more than one year)<br>renal tubular acidosis type 1<br>paresthesia of the foot (time since onset: more than one year)<br>paresthesia of the hand (time since onset: more than one year)<br>sensory deficit, arm (distribution: unilateral time since onset: more than one year)<br>sensory deficit, lower extremity (distribution: unilateral time since onset: more than one year)<br>hypercalciuria<br>hematuria<br>morning stiffness (time since onset: more than one year)<br>wrist pain (time since onset: more than one year)<br>ankle pain (time since onset: more than one year)<br>abdominal pain (position: epigastric time since onset: more than one year)<br>proteinuria (intensity: trace to mild)<br>uric acid level (result: reduced)<br>dyspnea (severity: on exertion time since onset: one month to one year)<br>syncope<br>hearing deficit (laterality: unilateral)<br>papule on the trunk<br>pustule on the trunk<br>oral mucosal bleeding<br>inflammatory back pain<br>dermatitis<br>aphthous ulcerations of the oral cavity (time since onset: one month to one year)<br>arthritis (time since onset: more than one year)<br>Interleukin 2 receptor, elevated		CRP, elevated<br>lactate dehydrogenase level, elevated<br>serum creatinine level<br>spinal stenosis seen on imaging<br>cervical spine degeneration<br>disc herniation<br>pulmonary infiltrates<br>vomiting<br>complete loss of consciousness<br>antinuclear antibody level, elevated<br>anti-dsDNA antibodies<br>antineutrophil cytoplasmic antibodies present<br>antiphospholipid antibodies, elevated<br>ACE level<br>thyroid stimulating hormone level<br>intracranial hemorrhage<br>increased intracranial pressure<br>cerebral ischemia<br>peripheral edema (pitting)<br>foamy urine<br>GLA gene mutation<br>protein S level<br>protein C level<br>lupus anticoagulant antibodies, elevated<br>heart valve defect<br>myopathy<br>cerebrospinal fluid protein level<br>CSF lymphocytosis<br>leukocytosis in CSF<br>RBC count in CSF, elevated<br>bacteria in CSF<br>arrhythmias on ECG<br>AV block<br>elevated rheumatoid factor<br>anti-U1-RNP antibodies<br>anti-Sm antibodies<br>anti-SSA autoantibodies<br>anti-La (SS-B)-autoantibodies<br>anti-Jo 1 antibodies<br>anti-Scl-70<br>anti-PL 7 antibodies<br>anti-PL 12 antibodies<br>anti-alpha-fodrin antibodies<br>serum amyloid A, elevated<br>MEFV gene mutation<br>NLRP3 gene mutation<br>TNFRSF1A gene mutation
**74**							
**Visit**	Sex	Age	Confirmed Diagnoses	Present Risk Factors	Pathological Symptoms/Findings (with attributes)	Absent Risk Factors	Non-pathological Symptoms/Findings
**Top Five Fit, Top Fit**	Female	41			sternal tenderness<br>dyspnea<br>chest pain (respiration: exacerbates)<br>arthro-osteitis		
**Diagnosis**	Female	43	SAPHO Syndrom		sternal tenderness (time since onset: more than one year)<br>dyspnea (time since onset: more than one year)<br>chest pain (respiration: exacerbates time since onset: more than one year)<br>erythrocyte sedimentation rate (result: elevated)<br>CRP, elevated<br>osteomyelitis<br>leukocyte level (result: elevated)<br>Hyperostosis		complement C4 level<br>antinuclear antibody level, elevated<br>anti-dsDNA antibodies<br>anti-U1-RNP antibodies<br>anti-SSA autoantibodies<br>anti-La (SS-B)-autoantibodies<br>anti-Sm antibodies<br>anti-Scl-70<br>anti-Jo 1 antibodies<br>antineutrophil cytoplasmic antibodies present
**75**							
**Visit**	Sex	Age	Confirmed Diagnoses	Present Risk Factors	Pathological Symptoms/Findings (with attributes)	Absent Risk Factors	Non-pathological Symptoms/Findings
**Top Five Fit**	Female	48		alcohol abuse	peripheral edema (pitting)<br>localized arthralgia<br>gingival bleeding<br>thrombocytopenia<br>epistaxis<br>Factor VIII level (result: reduced)<br>von Willebrand factor level, decreased<br>Hepatitis B virus<br>hepatitis C virus<br>gamma gt level, elevated		tenderness, MCP joints<br>tenderness, PIP joints (finger)<br>tenderness, DIP joints (finger)<br>distended jugular veins<br>collateral vessels
**Diagnosis**	Female	48	Chronic Hepatitis C	alcohol abuse	peripheral edema (pitting) (time since onset: one month to one year)<br>gingival bleeding (time since onset: one month to one year)<br>tenderness, MCP joints<br>epistaxis (time since onset: one month to one year)<br>depressed mood<br>hopelessness<br>Factor VIII level (result: reduced)<br>von Willebrand factor level, decreased<br>Hepatitis B virus<br>hepatitis C virus<br>sensory deficit, hand (time since onset: one month to one year distribution: bilateral, symmetrical)<br>erythema of the finger joint (time since onset: one day to one week)<br>swelling, finger joints (time since onset: one day to one week)<br>erythema of the hand<br>swelling of the hand (laterality: bilateral)<br>MCP joint pain (laterality: bilateral)<br>ankle pain (laterality: bilateral)<br>knee pain (laterality: bilateral)<br>headache<br>nausea<br>constipation<br>limited range of motion of the ankle joint<br>crepitus in knee<br>tenderness, wrist<br>paresthesia of the first three fingers (distribution: symmetrical time since onset: one day to one week)<br>transaminase levels, elevated<br>complement C4 level (result: reduced)<br>anti-smooth-muscle antibodies<br>transferrin saturation (result: reduced)<br>iron level (result: reduced)<br>osteoarthritis of the spine<br>carpal tunnel syndrome		distended jugular veins<br>collateral vessels<br>thrombocytopenia<br>vomiting<br>diarrhea<br>elevated rheumatoid factor<br>anti-cyclic citrullinated protein antibodies<br>anti-U1-RNP antibodies<br>anti-SSA autoantibodies<br>anti-La (SS-B)-autoantibodies<br>anti-Sm antibodies<br>anti-Jo 1 antibodies<br>anti-Scl-70<br>anti-dsDNA antibodies<br>antineutrophil cytoplasmic antibodies present<br>anticardiolipin antibodies, elevated<br>complement C3 level<br>CRP, elevated<br>hemoglobin level<br>leukocyte level<br>bone erosion of a joint<br>peripheral polyneuropathy
**76**							
**Visit**	Sex	Age	Confirmed Diagnoses	Present Risk Factors	Pathological Symptoms/Findings (with attributes)	Absent Risk Factors	Non-pathological Symptoms/Findings
**Top Five Fit, Top Fit**	Male	54			sinusitis<br>diverticula<br>colitis<br>eosinophilic infiltrate		
**Diagnosis**	Male	58	Eosinophilic Granulomatosis with Polyangiitis (EGPA)		sinusitis<br>pulmonary nodules<br>diverticula<br>dyspnea (severity: daily activities time since onset: more than one year)<br>FEV1, decreased<br>colitis<br>CRP, elevated<br>nasal polyp<br>eosinophilia in blood<br>neutrophilia<br>bronchitis<br>oropharyngeal candidiasis<br>Total IgE level, elevated<br>anti-cyclic citrullinated protein antibodies<br>mean corpuscular volume (result: reduced)<br>glomerular filtration rate, reduced<br>creatine kinase level, elevated<br>alanine transaminase level, elevated<br>IgG4 level (result: elevated)<br>vitamin D level, decreased<br>eosinophilic infiltrate		hypoxemia<br>antinuclear antibody level, elevated<br>antineutrophil cytoplasmic antibodies present<br>anti-dsDNA antibodies<br>interleukin 6, elevated<br>mycobacterium tuberculosis in lower respiratory tract<br>granuloma<br>elevated rheumatoid factor<br>anti-U1-RNP antibodies<br>anti-Sm antibodies<br>anti-SSA autoantibodies<br>anti-Jo 1 antibodies<br>anti-Scl-70<br>MI-2 antibody titer<br>anti-EJ antibodies<br>anti-OJ antibodies<br>anti-Ku antibodies<br>anti-PL 7 antibodies<br>anti-PL 12 antibodies<br>anticardiolipin antibodies, elevated<br>anti-GBM antibodies present
**77**							
**Visit**	Sex	Age	Confirmed Diagnoses	Present Risk Factors	Pathological Symptoms/Findings (with attributes)	Absent Risk Factors	Non-pathological Symptoms/Findings

**Diagnosis**	Female	51	Sjogren's syndrome		dry eye (time since onset: more than one year)<br>dry mouth (time since onset: more than one year)<br>weight loss (more than 10% of body weight within six months: yes)<br>hyperhidrosis, generalized (night sweats: yes time since onset: one month to one year)<br>erosions and ulcerations of the oral cavity (time since onset: one month to one year)<br>oral mucosal bleeding (time since onset: one month to one year)<br>abdominal pain (position: lower right quadrant time since onset: one month to one year eating: no effect)<br>diarrhea (time since onset: one month to one year)<br>foul smelling stool<br>thigh pain (laterality: bilateral time since onset: one month to one year)<br>headache (time since onset: one month to one year)<br>uterine mass seen on imaging<br>hepatic mass seen on imaging<br>esophagitis<br>gastric mucosal inflammation<br>sensory deficit, upper leg (time since onset: one month to one year)<br>reduced exercise tolerance (time since onset: one month to one year)<br>brain white matter lesion<br>finger joint pain (time since onset: one month to one year)<br>livedo<br>cachexia<br>alkaline phosphatase level (result: reduced)<br>cholesterol level (result: elevated)<br>serum protein level (result: elevated)<br>abdominal tenderness (position: pelvic)<br>anti-alpha-fodrin antibodies<br>painful dysesthesia of the lower extremity<br>tricolor pattern of discoloration of the fingers and toes<br>vaginal wall bleeding<br>hyposalivation		CRP, elevated<br>erythrocyte sedimentation rate<br>proteinase 3 antibody titer, elevated<br>myeloperoxidase antibodies present<br>anti-GBM antibodies present<br>anemia<br>leukocyte level<br>platelet count<br>edema (pitting)<br>serum complement level<br>elevated rheumatoid factor<br>anti-cyclic citrullinated protein antibodies<br>anti-dsDNA antibodies<br>anticardiolipin antibodies, elevated<br>anti-U1-RNP antibodies<br>anti-SSA autoantibodies<br>anti-La (SS-B)-autoantibodies<br>anti-Sm antibodies<br>anti-Jo 1 antibodies<br>anti-Scl-70<br>fever<br>ferritin level
**78**							
**Visit**	Sex	Age	Confirmed Diagnoses	Present Risk Factors	Pathological Symptoms/Findings (with attributes)	Absent Risk Factors	Non-pathological Symptoms/Findings
**Diagnosis**	Female	78	Sjogren's syndrome	Hx: hypertension	obstructive ventilation pattern<br>pulmonary nodules<br>bronchitis<br>oropharyngeal candidiasis<br>sliding hiatal hernia<br>ground-glass opacities<br>bronchiectasis<br>neutrophilia<br>erythrocyte sedimentation rate (result: elevated)<br>CRP, elevated<br>serum creatinine level (result: elevated)<br>back pain<br>liver cyst<br>abdominal pain (position: epigastric)<br>nausea<br>dysphagia<br>sputum<br>heartburn<br>glomerular filtration rate, reduced<br>parathyroid hormone level (result: elevated)<br>swelling, ankle joint<br>fatigue<br>crackles<br>mycobacterium avium-intracellulare in blood<br>renal cysts<br>small kidneys<br>blood urea nitrogen level (result: elevated)<br>anti-alpha-fodrin antibodies<br>dizziness<br>palpitation<br>thigh pain<br>FEV1/FVC (result: reduced)<br>Premature ventricular contraction<br>hyposalivation<br>dry mouth<br>creatine kinase level, elevated<br>tricolor pattern of discoloration of the fingers and toes<br>diffusion capacity (result: reduced)		cough<br>malignant cells in pulmonary tissue<br>positive tuberculin skin test<br>leukocytosis<br>pulmonary infiltrates<br>pleural effusion<br>proteinuria<br>hematuria<br>anti-U1-RNP antibodies<br>anti-La (SS-B)-autoantibodies<br>antineutrophil cytoplasmic antibodies present<br>anti-SSA autoantibodies<br>anemia<br>weight loss<br>peripheral edema (pitting)<br>axillary lymphadenopathy<br>hilar lymphadenopathy<br>stenosis of the renal artery<br>renal perfusion, decreased<br>thrombocytosis<br>antinuclear antibody level, elevated<br>complement C3 level<br>complement C4 level<br>anti-PL 7 antibodies<br>AV block<br>hypolacrimation<br>anti-Jo 1 antibodies<br>partial thromboplastin time<br>anti-PL 12 antibodies<br>anti-OJ antibodies<br>anti-EJ antibodies<br>positive interferon gamma release assay for tuberculosis
**79**							
**Visit**	Sex	Age	Confirmed Diagnoses	Present Risk Factors	Pathological Symptoms/Findings (with attributes)	Absent Risk Factors	Non-pathological Symptoms/Findings
**Top Five Fit, Top Fit**	Female	30			hip pain (time since onset: one month to one year laterality: unilateral activity: exacerbates)<br>groin pain (time since onset: one month to one year)<br>osteoarthritis of the knee<br>meniscal tear<br>knee pain (laterality: unilateral)<br>chondrodysplasia		
**Diagnosis**	Female	38	Stickler Syndrom		hip pain (time since onset: more than one year laterality: unilateral activity: exacerbates)<br>groin pain (time since onset: more than one year)<br>osteoarthritis of the knee<br>meniscal tear<br>knee pain (laterality: unilateral time since onset: more than one year)<br>gastric mucosal inflammation<br>ovarian cyst seen on imaging<br>varicose veins<br>swelling, knee (time since onset: more than one year)<br>genu valgum<br>pes cavus<br>arthritis, knee (time since onset: one month to one year)<br>renal cysts<br>low blood pressure<br>arthritis, elbow<br>glaucoma<br>swelling of the lower leg<br>dyspnea (severity: on exertion)<br>weight loss<br>tall stature<br>myopia<br>microhematuria<br>disc herniation<br>reduced visual acuity<br>finger joint pain<br>chondrodysplasia		dysuria<br>flank pain<br>macrohematuria<br>foamy urine<br>renal insufficiency<br>complement C3 level<br>complement C4 level<br>erythrocyte sedimentation rate<br>eosinophilia in blood
**80**							
**Visit**	Sex	Age	Confirmed Diagnoses	Present Risk Factors	Pathological Symptoms/Findings (with attributes)	Absent Risk Factors	Non-pathological Symptoms/Findings
**Top Five Fit, Top Fit**	Female	23			uveitis<br>hyperopia<br>inflammatory back pain<br>lower back pain (nocturnal or early morning: exacerbates activity: relieves time since onset: one month to one year)<br>gluteal pain<br>morning stiffness<br>dry eye		plaque of the skin<br>scaling of the skin, localized<br>urethritis<br>tricolor pattern of discoloration of the fingers and toes<br>aphthous ulcerations of the oral cavity<br>Lymphadenopathy on examination<br>photosensitivity of the skin<br>recurrent blood clots<br>swelling, joint<br>HLA-B27 positive<br>sacroiliitis<br>erosions and ulcerations of the large intestine<br>lactose intolerance<br>spinal deformity
**Diagnosis**	Female	23	Spondyloarthritis		uveitis<br>hyperopia<br>inflammatory back pain<br>lower back pain (nocturnal or early morning: exacerbates activity: relieves time since onset: one month to one year)<br>gluteal pain (time since onset: one month to one year)<br>dry eye<br>limited range of motion of the spine<br>microhematuria<br>vitamin D level, decreased<br>kyphosis		morning stiffness<br>plaque of the skin<br>scaling of the skin, localized<br>urethritis<br>tricolor pattern of discoloration of the fingers and toes<br>aphthous ulcerations of the oral cavity<br>Lymphadenopathy on examination<br>photosensitivity of the skin<br>recurrent blood clots<br>swelling, joint<br>HLA-B27 positive<br>sacroiliitis<br>erosions and ulcerations of the large intestine<br>lactose intolerance<br>spinal deformity<br>spinal tenderness<br>antinuclear antibody level, elevated<br>elevated rheumatoid factor<br>anti-cyclic citrullinated protein antibodies<br>serum complement level<br>antineutrophil cytoplasmic antibodies present<br>thyroid stimulating hormone level<br>parathyroid hormone level
**81**							
**Visit**	Sex	Age	Confirmed Diagnoses	Present Risk Factors	Pathological Symptoms/Findings (with attributes)	Absent Risk Factors	Non-pathological Symptoms/Findings
**Top Five Fit**	Female	20		smoker<br>elevated BMI	proteinuria (intensity: trace to mild)<br>striae<br>CRP, elevated<br>erythrocyte sedimentation rate (result: elevated)<br>glomerular filtration rate, reduced<br>alpha 2 globulin level (result: elevated)<br>urinary pH level (pH value: pH <5.5)<br>microhematuria<br>cholesterol level (result: elevated)<br>triglyceride level (result: elevated)<br>uric acid level (result: elevated)	diabetes mellitus<br>Hx: hypertension	peripheral edema (pitting)<br>diarrhea<br>loss of appetite<br>serum glucose level<br>malaise<br>potassium level
**Diagnosis**	Female	21	Focal segmental glomerulosclerosis (FSGS)	smoker<br>elevated BMI	proteinuria (intensity: trace to mild)<br>striae<br>erythrocyte sedimentation rate (result: elevated)<br>glomerular filtration rate, reduced<br>alpha 2 globulin level (result: elevated)<br>triglyceride level (result: elevated)<br>serum protein level (result: reduced)<br>increased urinary frequency<br>erythema of the skin, localized (scaly surface: yes)<br>serum albumin level (result: reduced)<br>sodium level (result: reduced)<br>blood urea nitrogen level (result: elevated)<br>transaminase levels, elevated<br>focal segmental glomerulosclerosis<br>nephrosclerosis<br>uric acid level (result: elevated)	Hx: hypertension<br>diabetes mellitus	peripheral edema (pitting)<br>diarrhea<br>CRP, elevated<br>loss of appetite<br>serum glucose level<br>microhematuria<br>malaise<br>cholesterol level<br>potassium level<br>cough<br>dysuria<br>fever<br>reduced exercise tolerance
**82**							
**Visit**	Sex	Age	Confirmed Diagnoses	Present Risk Factors	Pathological Symptoms/Findings (with attributes)	Absent Risk Factors	Non-pathological Symptoms/Findings

**Top Five Fit, Top Fit**	Female	14			ventricular septal defect<br>dyspnea<br>splenomegaly<br>systolic heart murmur<br>aortic valve regurgitation on imaging<br>right bundle branch block<br>reduced exercise tolerance<br>fever (time since onset: one day to one week)<br>increased urinary frequency<br>hand pain (laterality: unilateral)<br>posterior neck pain<br>underweight<br>cough (clear or yellow phlegm: no)<br>erythrocyte sedimentation rate (result: elevated)<br>generalized arthralgia<br>platelet count (result: elevated)<br>glomerular filtration rate, reduced<br>proteinuria (intensity: trace to mild)<br>microcytic anemia<br>weight loss (more than 10% of body weight within six months: no)<br>secondary amenorrhea<br>shoulder asymmetry<br>flank pain (side: right)<br>interstitial nephritis<br>blood urea nitrogen level (result: elevated)<br>endocarditis		cyanosis<br>edema (pitting)<br>hepatomegaly<br>arrhythmic pulse on palpation<br>heart valve defect<br>hypoxemia<br>arrhythmias on ECG<br>supraventricular premature beats<br>Premature ventricular contraction<br>cardiomegaly<br>chest pain<br>antistreptolysin O antibody titer, elevated<br>antineutrophil cytoplasmic antibodies present<br>antinuclear antibody level, elevated<br>anti-dsDNA antibodies<br>serum complement level<br>spinal tenderness<br>uveitis<br>hyperhidrosis, generalized
**Diagnosis**	Female	16	Tubulointerstitial nephritis and uveitis syndrome (TINU)		ventricular septal defect<br>dyspnea<br>splenomegaly<br>systolic heart murmur<br>aortic valve regurgitation on imaging<br>right bundle branch block<br>reduced exercise tolerance<br>fever (high grade: yes time since onset: one day to one week)<br>increased urinary frequency<br>hand pain (laterality: unilateral)<br>posterior neck pain<br>underweight<br>cough (clear or yellow phlegm: no)<br>erythrocyte sedimentation rate (result: elevated)<br>generalized arthralgia<br>platelet count (result: elevated)<br>glomerular filtration rate, reduced<br>microcytic anemia<br>weight loss (more than 10% of body weight within six months: no)<br>secondary amenorrhea<br>shoulder asymmetry<br>flank pain (side: right time since onset: one month to one year)<br>interstitial nephritis<br>blood urea nitrogen level (result: elevated)<br>endocarditis<br>anterior uveitis		cyanosis<br>edema (pitting)<br>hepatomegaly<br>arrhythmic pulse on palpation<br>heart valve defect<br>hypoxemia<br>arrhythmias on ECG<br>supraventricular premature beats<br>Premature ventricular contraction<br>cardiomegaly<br>chest pain<br>antistreptolysin O antibody titer, elevated<br>proteinuria<br>antineutrophil cytoplasmic antibodies present<br>antinuclear antibody level, elevated<br>anti-dsDNA antibodies<br>serum complement level<br>spinal tenderness<br>hyperhidrosis, generalized<br>gastroesophageal reflux<br>exocrine dysfunction of the pancreas<br>restrictive ventilation pattern<br>obstructive ventilation pattern<br>Lymphadenopathy on palpation<br>crackles<br>abdominal tenderness
**83**							
**Visit**	Sex	Age	Confirmed Diagnoses	Present Risk Factors	Pathological Symptoms/Findings (with attributes)	Absent Risk Factors	Non-pathological Symptoms/Findings
**Top Five Fit**	Female	36			generalized arthralgia (time since onset: more than one year)<br>abdominal pain (time since onset: more than one year)<br>crepitus in knee<br>feeling of lump in the throat<br>palpitation (time since onset: more than one year)<br>enlarged thyroid gland<br>rapid pulse (time since onset: more than one year)<br>atrioventricular re-entrant tachycardia<br>right bundle branch block<br>myalgia (time since onset: more than one year)<br>sensory deficit<br>upper extremity paresis<br>headache<br>gastroesophageal reflux<br>neuropathic pattern on EMG<br>antinuclear antibody level, elevated<br>tricolor pattern of discoloration of the fingers and toes<br>complement C3 level (result: reduced)<br>knee pain<br>PIP joint pain (finger)<br>elbow pain<br>painful dysesthesia of the upper extremity<br>painful dysesthesia of the trunk<br>posterior neck pain<br>dysphagia (time since onset: one month to one year)<br>increased tearing<br>arthritis<br>proximal lower extremity paresis		swelling, joint<br>fever<br>urinary tract infection<br>skin rash<br>diarrhea<br>spinal tenderness<br>thyroglobulin antibodies<br>thyrotropin receptor antibodies<br>thyroid nodule on imaging<br>dry mouth<br>disorientation<br>muscle atrophy<br>muscular hypertonicity<br>muscular hypotonicity<br>clonus<br>cranial nerve disorder<br>complement C4 level<br>CRP, elevated<br>creatine kinase level, elevated<br>serum creatinine level<br>anemia<br>lupus anticoagulant antibodies, elevated<br>elevated rheumatoid factor<br>immune deposits<br>antineutrophil cytoplasmic antibodies present<br>ACE level<br>anti-dsDNA antibodies<br>anti-U1-RNP antibodies<br>anti-Sm antibodies<br>anti-SSA autoantibodies<br>anti-La (SS-B)-autoantibodies<br>anti-Scl-70<br>anti-Jo 1 antibodies<br>erythema of the joint<br>morning stiffness<br>dry eye<br>photophobia<br>weight loss<br>hyperhidrosis, generalized
**Top Fit**	Female	37			generalized arthralgia (time since onset: more than one year)<br>abdominal pain (time since onset: more than one year)<br>crepitus in knee<br>feeling of lump in the throat<br>palpitation (time since onset: more than one year)<br>enlarged thyroid gland<br>rapid pulse (time since onset: more than one year)<br>atrioventricular re-entrant tachycardia<br>right bundle branch block<br>myalgia (time since onset: more than one year)<br>sensory deficit<br>upper extremity paresis<br>headache<br>gastroesophageal reflux<br>neuropathic pattern on EMG<br>antinuclear antibody level, elevated<br>tricolor pattern of discoloration of the fingers and toes<br>complement C3 level (result: reduced)<br>anemia<br>knee pain<br>PIP joint pain (finger)<br>elbow pain<br>painful dysesthesia of the upper extremity<br>painful dysesthesia of the trunk<br>posterior neck pain<br>dysphagia (time since onset: one month to one year)<br>increased tearing<br>arthritis<br>muscle fasciculation<br>tenderness, MCP joints<br>tenderness, MTP joints<br>anti-PL 7 antibodies<br>proximal lower extremity paresis		swelling, joint<br>fever<br>urinary tract infection<br>skin rash<br>diarrhea<br>spinal tenderness<br>thyroglobulin antibodies<br>thyrotropin receptor antibodies<br>thyroid nodule on imaging<br>dry mouth<br>disorientation<br>muscle atrophy<br>muscular hypertonicity<br>muscular hypotonicity<br>clonus<br>cranial nerve disorder<br>complement C4 level<br>CRP, elevated<br>creatine kinase level, elevated<br>serum creatinine level<br>lupus anticoagulant antibodies, elevated<br>elevated rheumatoid factor<br>immune deposits<br>antineutrophil cytoplasmic antibodies present<br>ACE level<br>anti-dsDNA antibodies<br>anti-U1-RNP antibodies<br>anti-Sm antibodies<br>anti-SSA autoantibodies<br>anti-La (SS-B)-autoantibodies<br>anti-Scl-70<br>anti-Jo 1 antibodies<br>erythema of the joint<br>morning stiffness<br>dry eye<br>photophobia<br>weight loss<br>hyperhidrosis, generalized<br>leukopenia<br>neutropenia<br>anti-cyclic citrullinated protein antibodies<br>uveitis<br>conjunctivitis<br>erythema<br>anti-SRP antibodies<br>anti-Ku antibodies<br>anti-PL 12 antibodies<br>anticardiolipin antibodies, elevated<br>anti-EJ antibodies<br>anti-OJ antibodies<br>platelet count

**Diagnosis**	Female	37	Antisynthetase Syndrome		generalized arthralgia (time since onset: more than one year)<br>abdominal pain (time since onset: more than one year)<br>crepitus in knee<br>feeling of lump in the throat<br>palpitation (time since onset: more than one year)<br>enlarged thyroid gland<br>rapid pulse (time since onset: more than one year)<br>atrioventricular re-entrant tachycardia<br>right bundle branch block<br>myalgia (time since onset: more than one year)<br>sensory deficit<br>upper extremity paresis<br>headache<br>gastroesophageal reflux<br>neuropathic pattern on EMG<br>antinuclear antibody level, elevated<br>tricolor pattern of discoloration of the fingers and toes<br>complement C3 level (result: reduced)<br>knee pain<br>PIP joint pain (finger)<br>elbow pain<br>painful dysesthesia of the upper extremity<br>painful dysesthesia of the trunk<br>posterior neck pain<br>dysphagia (time since onset: one month to one year)<br>increased tearing<br>arthritis<br>muscle fasciculation<br>tenderness, MCP joints<br>tenderness, MTP joints<br>anti-PL 7 antibodies<br>aphthous ulcerations of the oral cavity (time since onset: more than one year)<br>serum chromogranin A/B, elevated<br>proximal lower extremity paresis		swelling, joint<br>fever<br>urinary tract infection<br>skin rash<br>diarrhea<br>spinal tenderness<br>thyroglobulin antibodies<br>thyrotropin receptor antibodies<br>thyroid nodule on imaging<br>dry mouth<br>disorientation<br>muscle atrophy<br>muscular hypertonicity<br>muscular hypotonicity<br>clonus<br>cranial nerve disorder<br>complement C4 level<br>CRP, elevated<br>creatine kinase level, elevated<br>serum creatinine level<br>anemia<br>lupus anticoagulant antibodies, elevated<br>elevated rheumatoid factor<br>immune deposits<br>antineutrophil cytoplasmic antibodies present<br>ACE level<br>anti-dsDNA antibodies<br>anti-U1-RNP antibodies<br>anti-Sm antibodies<br>anti-SSA autoantibodies<br>anti-La (SS-B)-autoantibodies<br>anti-Scl-70<br>anti-Jo 1 antibodies<br>erythema of the joint<br>morning stiffness<br>dry eye<br>photophobia<br>weight loss<br>hyperhidrosis, generalized<br>leukopenia<br>anti-cyclic citrullinated protein antibodies<br>uveitis<br>conjunctivitis<br>erythema<br>anti-SRP antibodies<br>anti-Ku antibodies<br>anti-PL 12 antibodies<br>anticardiolipin antibodies, elevated<br>anti-EJ antibodies<br>anti-OJ antibodies
**84**							
**Visit**	Sex	Age	Confirmed Diagnoses	Present Risk Factors	Pathological Symptoms/Findings (with attributes)	Absent Risk Factors	Non-pathological Symptoms/Findings
**Diagnosis**	Male	48	SAPHO-Syndrome	diabetes mellitus<br>elevated BMI	obstructive sleep apnea (time since onset: more than one year)<br>aphthous ulcerations of the oral cavity (time since onset: more than one year)<br>headache (time since onset: one month to one year)<br>effusion, ankle<br>osteoarthritis<br>kyphosis<br>memory difficulty (time since onset: more than one year)<br>ankle pain (time since onset: more than one year laterality: bilateral)<br>pustule on the foot<br>disc degeneration<br>osteomyelitis<br>back pain (time since onset: more than one year)		nasal discharge<br>ankle fracture
**85**							
**Visit**	Sex	Age	Confirmed Diagnoses	Present Risk Factors	Pathological Symptoms/Findings (with attributes)	Absent Risk Factors	Non-pathological Symptoms/Findings
**Top Five Fit**	Male	52			headache (time since onset: one week to one month)<br>sore throat (time since onset: one week to one month)<br>fever (high grade: no time since onset: one week to one month)<br>myalgia (time since onset: one week to one month)<br>groin pain (time since onset: one week to one month)<br>scrotal pain (time since onset: one week to one month)<br>lower extremity pain (time since onset: one week to one month)<br>hyperhidrosis, generalized (night sweats: yes time since onset: one week to one month)<br>weight loss (more than 10% of body weight within six months: no)<br>nocturia<br>CRP, elevated<br>vitamin D level, decreased<br>parathyroid hormone level (result: elevated)<br>elevated rheumatoid factor<br>myositis		venous thrombosis<br>IgA level<br>IgG level<br>antinuclear antibody level, elevated<br>antineutrophil cytoplasmic antibodies present<br>anti-dsDNA antibodies<br>anti-SSA autoantibodies<br>anti-La (SS-B)-autoantibodies<br>anti-Sm antibodies<br>anti-Jo 1 antibodies<br>anti-U1-RNP antibodies<br>anti-Scl-70<br>Hepatitis B virus<br>hepatitis C virus<br>confirmed human immunodeficiency virus infection<br>Treponema pallidum antigen
**Top Fit**<br>**(<= 1 month early)**	Male	52			headache (time since onset: one month to one year)<br>sore throat (time since onset: one month to one year)<br>fever (high grade: no time since onset: one month to one year)<br>myalgia (time since onset: one month to one year)<br>groin pain (time since onset: one month to one year)<br>scrotal pain (time since onset: one month to one year)<br>lower extremity pain (time since onset: one month to one year)<br>hyperhidrosis, generalized (night sweats: yes time since onset: one month to one year)<br>weight loss (more than 10% of body weight within six months: no)<br>nocturia (time since onset: one month to one year)<br>CRP, elevated<br>vitamin D level, decreased<br>parathyroid hormone level (result: elevated)<br>elevated rheumatoid factor<br>myositis<br>anemia<br>leukocytosis<br>thrombocytopenia		venous thrombosis<br>IgA level<br>IgG level<br>antinuclear antibody level, elevated<br>antineutrophil cytoplasmic antibodies present<br>anti-dsDNA antibodies<br>anti-SSA autoantibodies<br>anti-La (SS-B)-autoantibodies<br>anti-Sm antibodies<br>anti-Jo 1 antibodies<br>anti-U1-RNP antibodies<br>anti-Scl-70<br>Hepatitis B virus<br>hepatitis C virus<br>confirmed human immunodeficiency virus infection<br>Treponema pallidum antigen
**Diagnosis**	Male	52	Polymyositis		headache (time since onset: one month to one year)<br>sore throat (time since onset: one month to one year)<br>fever (high grade: no time since onset: one month to one year)<br>myalgia (time since onset: one month to one year)<br>groin pain (time since onset: one month to one year)<br>scrotal pain (time since onset: one month to one year)<br>lower extremity pain (time since onset: one month to one year)<br>hyperhidrosis, generalized (night sweats: yes time since onset: one month to one year)<br>weight loss (more than 10% of body weight within six months: no)<br>nocturia (time since onset: one month to one year)<br>CRP, elevated<br>vitamin D level, decreased<br>parathyroid hormone level (result: elevated)<br>elevated rheumatoid factor<br>myositis<br>anemia<br>leukocytosis<br>thrombocytopenia<br>sodium level (result: elevated)<br>gamma gt level, elevated<br>IgG4 level (result: elevated)<br>complement C4 level (result: elevated)<br>proteinase 3 antibody titer, elevated<br>anti-smooth-muscle antibodies<br>glomerular filtration rate, reduced<br>cardiac ejection fraction (result: reduced)		venous thrombosis<br>IgA level<br>IgG level<br>antinuclear antibody level, elevated<br>antineutrophil cytoplasmic antibodies present<br>anti-dsDNA antibodies<br>anti-SSA autoantibodies<br>anti-La (SS-B)-autoantibodies<br>anti-Sm antibodies<br>anti-Jo 1 antibodies<br>anti-U1-RNP antibodies<br>anti-Scl-70<br>Hepatitis B virus<br>hepatitis C virus<br>confirmed human immunodeficiency virus infection<br>Treponema pallidum antigen<br>antimitochondrial antibody<br>anti-soluble liver antigens antibodies<br>parietal cell antibodies<br>heart valve defect<br>partial thromboplastin time
**86**							
**Visit**	Sex	Age	Confirmed Diagnoses	Present Risk Factors	Pathological Symptoms/Findings (with attributes)	Absent Risk Factors	Non-pathological Symptoms/Findings
**Top Five Fit, Top Fit**	Female	32			gastric mucosal inflammation<br>antinuclear antibody level, elevated<br>anti-SSA autoantibodies<br>IgG level (result: elevated)<br>intestinal inflammation<br>wrist pain (laterality: bilateral time since onset: one month to one year)<br>finger joint pain (laterality: bilateral time since onset: one month to one year)<br>tarsometatarsal joint pain (laterality: bilateral time since onset: one month to one year)<br>ankle pain (laterality: bilateral time since onset: one month to one year)<br>knee pain (laterality: bilateral time since onset: one month to one year)<br>myalgia (time since onset: one month to one year)<br>muscle cramps (time since onset: one month to one year)<br>morning stiffness<br>dry mouth<br>headache (laterality: unilateral)<br>swelling, wrist<br>swelling, knee (laterality: bilateral)<br>tenderness, MCP joints (laterality: bilateral)<br>tenderness, MTP joints (laterality: bilateral)<br>Total IgE level, elevated<br>elevated rheumatoid factor<br>microcytic anemia<br>procalcitonin level, elevated		anti-U1-RNP antibodies<br>anti-Sm antibodies<br>anti-La (SS-B)-autoantibodies<br>antineutrophil cytoplasmic antibodies present<br>C3 nephritic factor level, elevated<br>IgA level<br>complement C3 level<br>complement C4 level<br>tubular necrosis<br>focal segmental glomerulosclerosis<br>serum creatinine level<br>glomerular filtration rate, reduced<br>proteinuria<br>hematuria<br>anti-Jo 1 antibodies<br>anti-alpha-fodrin antibodies<br>anticardiolipin antibodies, elevated<br>anti-dsDNA antibodies<br>creatine kinase level, elevated<br>leukocyte level<br>platelet count<br>CRP, elevated<br>transaminase levels, elevated<br>lactate dehydrogenase level, elevated<br>diffusion capacity<br>neutropenia<br>splenomegaly<br>hepatomegaly<br>bone erosion of a joint

**Diagnosis**	Female	33	Chronic Polyarthritis<br>Sjogren's syndrome		gastric mucosal inflammation<br>antinuclear antibody level, elevated<br>anti-SSA autoantibodies<br>IgG level (result: elevated)<br>intestinal inflammation<br>wrist pain (laterality: bilateral time since onset: one month to one year)<br>finger joint pain (laterality: bilateral time since onset: one month to one year)<br>tarsometatarsal joint pain (laterality: bilateral time since onset: one month to one year)<br>ankle pain (laterality: bilateral time since onset: one month to one year)<br>knee pain (laterality: bilateral time since onset: one month to one year)<br>myalgia (time since onset: one month to one year)<br>muscle cramps (time since onset: one month to one year)<br>morning stiffness<br>dry mouth<br>headache (laterality: unilateral time since onset: more than one year)<br>swelling, wrist<br>swelling, knee (laterality: bilateral)<br>tenderness, MCP joints (laterality: bilateral)<br>tenderness, MTP joints (laterality: bilateral)<br>Total IgE level, elevated<br>elevated rheumatoid factor<br>microcytic anemia<br>procalcitonin level, elevated<br>hypolacrimation<br>anti-cyclic citrullinated protein antibodies		anti-U1-RNP antibodies<br>anti-Sm antibodies<br>anti-La (SS-B)-autoantibodies<br>antineutrophil cytoplasmic antibodies present<br>C3 nephritic factor level, elevated<br>IgA level<br>complement C3 level<br>complement C4 level<br>tubular necrosis<br>focal segmental glomerulosclerosis<br>serum creatinine level<br>glomerular filtration rate, reduced<br>proteinuria<br>hematuria<br>anti-Jo 1 antibodies<br>anti-alpha-fodrin antibodies<br>anticardiolipin antibodies, elevated<br>anti-dsDNA antibodies<br>creatine kinase level, elevated<br>leukocyte level<br>platelet count<br>CRP, elevated<br>transaminase levels, elevated<br>lactate dehydrogenase level, elevated<br>diffusion capacity<br>neutropenia<br>splenomegaly<br>hepatomegaly<br>bone erosion of a joint
**87**							
**Visit**	Sex	Age	Confirmed Diagnoses	Present Risk Factors	Pathological Symptoms/Findings (with attributes)	Absent Risk Factors	Non-pathological Symptoms/Findings
**Diagnosis**	Male	40	Spondyloarthritis	smoker	chest pain (position: retrosternal radiating: yes respiration: exacerbates time since onset: one month to one year)<br>heartburn<br>fever (time since onset: one month to one year)<br>reduced exercise tolerance (time since onset: more than one year)<br>CRP, elevated<br>leukocytosis<br>crackles<br>polyserositis<br>fatigue (time since onset: more than one year)<br>alanine transaminase level, elevated<br>gamma gt level, elevated<br>lower back pain (radiating down the leg: yes time since onset: one month to one year)<br>inflammatory back pain<br>morning stiffness<br>knee pain (time since onset: more than one year laterality: bilateral)<br>elbow pain (time since onset: more than one year laterality: bilateral)<br>erosion and ulceration of the back (time since onset: more than one year)<br>limited range of motion of the spine<br>chest tightness<br>shortened PR interval on ECG<br>steatosis hepatis<br>hepatic mass seen on imaging<br>prostate enlargement<br>diffusion capacity (result: reduced)<br>sacroiliitis<br>Interleukin 2 receptor, elevated<br>positive interferon gamma release assay for tuberculosis		pericardial effusion<br>pleural effusion<br>erythrocyte sedimentation rate<br>procalcitonin level, elevated<br>cardiac ejection fraction<br>anti-SSA autoantibodies<br>anti-OJ antibodies<br>anti-EJ antibodies<br>anti-PL 12 antibodies<br>anti-PL 7 antibodies<br>anti-SRP antibodies<br>anti-Jo 1 antibodies<br>anti-PmScl antibody titer<br>anti-Ku antibodies<br>MI-2 antibody titer<br>MEFV gene mutation<br>glomerular filtration rate, reduced<br>anemia<br>platelet count<br>TNFRSF1A gene mutation<br>NLRP3 gene mutation<br>swelling, joint<br>abdominal lymphadenopathy<br>pulmonary infiltrates<br>HLA-B27 positive
**88**							
**Visit**	Sex	Age	Confirmed Diagnoses	Present Risk Factors	Pathological Symptoms/Findings (with attributes)	Absent Risk Factors	Non-pathological Symptoms/Findings
**Diagnosis**	Male	45	Behcet's disease<br>Spondyloarthritis	smoker	headache (laterality: unilateral time since onset: more than one year)<br>sensory deficit, arm (distribution: unilateral time since onset: more than one year)<br>sensory deficit, lower extremity (distribution: unilateral time since onset: more than one year)<br>double vision (time since onset: more than one year)<br>impaired vision (time since onset: more than one year)<br>gait impairment<br>leukocytosis<br>vitamin D level, decreased<br>exophthalmos<br>hypolacrimation<br>kidney stone<br>renal tubular acidosis type 1<br>vesicle on the trunk (erythematous: yes pain: yes pruritus: yes)<br>reduced exercise tolerance (time since onset: one month to one year)<br>dyspnea (severity: on exertion)<br>aural fullness<br>mitral valve regurgitation on imaging<br>vertigo<br>ear noise (laterality: unilateral)<br>uric acid level (result: reduced)<br>thyroid stimulating hormone level (result: elevated)<br>hemiparesis<br>pustule on the trunk (time since onset: one month to one year)<br>dry mouth<br>neck pain<br>finger joint pain<br>aphthous ulcerations of the oral cavity (time since onset: one month to one year)<br>hearing deficit (laterality: unilateral time since onset: one month to one year)<br>arthritis, other location		ataxia<br>disorientation<br>meningism<br>Lhermitte's sign<br>cranial nerve disorder<br>lower extremity paresis<br>upper extremity paresis<br>extensor plantar reflex<br>clonus<br>ischemic infarction<br>intracerebral hemorrhage<br>intracranial hemorrhage<br>galactorrhea<br>chest pain<br>weight loss<br>blood pressure<br>cardiac ejection fraction<br>left ventricular hypertrophy<br>heart valve defect<br>positive pathergy test<br>elevated rheumatoid factor<br>ACE level<br>beta 2 microglobulin level, elevated<br>pathologically increased cryoglobulin level<br>uveitis
**89**							
**Visit**	Sex	Age	Confirmed Diagnoses	Present Risk Factors	Pathological Symptoms/Findings (with attributes)	Absent Risk Factors	Non-pathological Symptoms/Findings
**Top Five Fit, Top Fit**	Female	81		Hx: hypertension	headache (time since onset: one day to one week bending forward: exacerbates)<br>alanine transaminase level, elevated<br>aspartate transaminase level, elevated<br>hepatitis C virus<br>antinuclear antibody level, elevated<br>erythrocyte sedimentation rate (result: elevated)<br>platelet count (result: reduced)<br>CRP, elevated<br>anti-smooth-muscle antibodies<br>antimitochondrial antibody<br>leukocytoclastic vasculitis		antineutrophil cytoplasmic antibodies present<br>leukocyte level<br>hemoglobin level
**Diagnosis**	Female	81	Cryoglobulinemia	Hx: hypertension	headache (time since onset: one month to one year bending forward: exacerbates)<br>alanine transaminase level, elevated<br>aspartate transaminase level, elevated<br>hepatitis C virus<br>antinuclear antibody level, elevated<br>erythrocyte sedimentation rate (result: elevated)<br>CRP, elevated<br>anti-smooth-muscle antibodies<br>antimitochondrial antibody<br>leukocytoclastic vasculitis<br>lower extremity pain<br>complement C3 level (result: reduced)<br>complement C4 level (result: reduced)<br>pathologically increased cryoglobulin level<br>peripheral polyneuropathy<br>tricolor pattern of discoloration of the fingers and toes<br>feeling of heavy legs<br>swelling of the lower extremity<br>foot pain<br>fatigue<br>petechiae	alcohol abuse<br>smoker	antineutrophil cytoplasmic antibodies present<br>leukocyte level<br>hemoglobin level<br>platelet count<br>abdominal pain<br>serum creatinine level<br>alkaline phosphatase level<br>anti-U1-RNP antibodies<br>anti-Sm antibodies<br>anti-SSA autoantibodies<br>anti-La (SS-B)-autoantibodies<br>proteinase 3 antibody titer, elevated<br>myeloperoxidase antibodies present<br>anti-Scl-70<br>anti-dsDNA antibodies<br>cold agglutinin<br>erosions and ulcerations of the skin
**90**							
**Visit**	Sex	Age	Confirmed Diagnoses	Present Risk Factors	Pathological Symptoms/Findings (with attributes)	Absent Risk Factors	Non-pathological Symptoms/Findings
**Diagnosis**	Female	52	Mixed Amyloidosis		dyspnea (time since onset: more than one year)<br>bone pain (time since onset: more than one year)<br>CRP, elevated<br>chest pain (position: unilateral)<br>diminished sense of taste<br>intestinal meteorism<br>renal insufficiency<br>proteinuria (intensity: moderate to severe)<br>serum amyloid A, elevated<br>amyloid protein deposition<br>Total IgE level, elevated<br>positive interferon gamma release assay for tuberculosis		erythrocyte sedimentation rate<br>mycobacterium tuberculosis in lower respiratory tract<br>mycobacterium tuberculosis in upper respiratory tract<br>mycobacterium tuberculosis in urine<br>mycobacterium tuberculosis in blood<br>positive tuberculin skin test
**91**							
**Visit**	Sex	Age	Confirmed Diagnoses	Present Risk Factors	Pathological Symptoms/Findings (with attributes)	Absent Risk Factors	Non-pathological Symptoms/Findings
**Diagnosis**	Female	39	Systemic Lupus Erythematosus (SLE)<br>Renal Cell Carcinoma		knee pain (time since onset: one month to one year activity: no effect intensity: severe colchicine: no effect alcohol: no effect laterality: bilateral diet rich in purine<br>deep vein thrombosis<br>pulmonary embolism<br>Factor V level (result: reduced)<br>arthritis<br>uveitis<br>antinuclear antibody level, elevated<br>anti-beta 2 glycoprotein antibodies, elevated<br>lymphopenia<br>microhematuria<br>proteinuria<br>renal insufficiency<br>renal mass seen on imaging<br>glomerulonephritis<br>malignant cells in renal tissue		anti-dsDNA antibodies<br>elevated rheumatoid factor<br>complement C3 level<br>complement C4 level
**92**							
**Visit**	Sex	Age	Confirmed Diagnoses	Present Risk Factors	Pathological Symptoms/Findings (with attributes)	Absent Risk Factors	Non-pathological Symptoms/Findings
**Top Five Fit**	Female	9			short stature<br>lower extremity pain (laterality: bilateral)		
**Top Fit**	Female	15			short stature<br>lower extremity pain (laterality: bilateral)<br>dental abnormalities<br>alopecia		

**Diagnosis**	Female	40	Hypophosphatasia		short stature<br>lower extremity pain (laterality: bilateral)<br>dental abnormalities<br>alopecia<br>proof of miscarriage<br>evidence of ectopic pregnancy in ultrasound<br>fatigue<br>unexplained fracture<br>alkaline phosphatase level (result: reduced)		parathyroid hormone level<br>vitamin D level, decreased
**93**							
**Visit**	Sex	Age	Confirmed Diagnoses	Present Risk Factors	Pathological Symptoms/Findings (with attributes)	Absent Risk Factors	Non-pathological Symptoms/Findings
**Top Five Fit**	Male	42			soft tissue lump on the face (time since onset: one month to one year change in size: yes tenderness: tender consistency: firm)		sensory deficit, face
**Diagnosis**	Male	42	Kimura Disease		soft tissue lump on the face (time since onset: one month to one year change in size: yes tenderness: tender consistency: firm)<br>preauricular lymphadenopathy (time since onset: one month to one year)<br>Total IgE level, elevated<br>eosinophilic infiltrate<br>antistreptolysin O antibody titer, elevated<br>anti-alpha-fodrin antibodies<br>parathyroid hormone level (result: elevated)<br>steatosis hepatis<br>blue skin<br>transaminase levels, elevated<br>gamma gt level, elevated<br>cholesterol level (result: elevated)		sensory deficit, face<br>facial muscle weakness
